# Liver sinusoidal endothelial cells in liver diseases and hepatocellular carcinoma: molecular mechanisms, physiology, and therapies

**DOI:** 10.1186/s43556-026-00527-z

**Published:** 2026-08-17

**Authors:** Mengyao Yan, Yimin Cui, Qian Xiang

**Affiliations:** 1 Institute of Clinical Pharmacology, Peking University First Hospital, Beijing, China; 2 Department of Pharmacy Administration and Clinical Pharmacy, School of Pharmaceutical Sciences, Peking University Health Science Center, Beijing, China

**Keywords:** Liver sinusoidal endothelial cells, LSEC reprogramming, Liver diseases, Hepatic stellate cells, Therapeutic strategies

## Abstract

Liver sinusoidal endothelial cells (LSECs) form a specialized discontinuous microvascular interface between sinusoidal blood and the hepatic parenchyma. Functioning as a central regulator of hepatic homeostasis, LSECs integrate metabolic, inflammatory, and hemodynamic signals to maintain sinusoidal permeability, immune tolerance, vascular tone, and lipid exchange. During acute injury or sustained hepatic stress, LSECs may lose their differentiated sinusoidal phenotype and acquire features of capillarization, including fenestrae loss, subendothelial matrix accumulation, impaired scavenging activity, and attenuation of KLF2-eNOS-NO signaling. Capillarized LSECs are not merely a byproduct of hepatic damage; instead, they may function as early endothelial sentinels and important contributors to the amplification of parenchymal injury, inflammation, fibrogenesis, and tumor immune evasion. This review summarizes the physiological functions of LSECs and examines mechanisms through which LSEC dysfunction contributes to acute liver injury, metabolic dysfunction-associated steatotic liver disease, viral hepatitis, cirrhosis, and hepatocellular carcinoma. By comparing conserved mechanisms with disease-specific spatial and molecular triggers, this review highlights how LSEC phenotypic switching disrupts the angiocrine–immune–fibrotic axis and contributes to disease progression. Recent advances in single-cell and spatial omics, LSEC subpopulation heterogeneity, and capillarization-aware therapeutic delivery are further discussed. Finally, therapeutic reprogramming of dysfunctional LSECs toward a quiescent, fenestrated, and tolerogenic phenotype is proposed as a strategy to restore sinusoidal homeostasis and improve treatment of liver diseases.

## Introduction

**Fig. 1:**
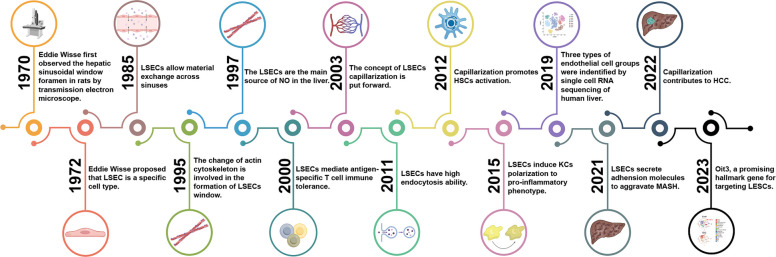
Timeline of the milestone events for LSECs. In 1970, Eddie Wisse used transmission electron microscopy to visualize fenestrae in rat hepatic sinusoids, helping distinguish LSECs from other liver cell types. In 1972, Wisse further proposed that LSECs constitute a distinct cell type. It was believed in 1985 that LSECs permitted cross-sinus material exchange. By the mid-1990s, changes in the actin cytoskeleton were recognized as important regulators of fenestra formation. Shah et al. demonstrated in 1997 that the primary source of hepatic NO was LSECs. Beginning in 2000, studies by Limmer and colleagues established that LSECs have immunomodulatory functions, including induction of tolerance to circulating soluble antigens and regulation of lymphocyte recruitment. After three years, the concept of capillarization was proposed. By 2011, the high endocytic capacity of LSECs was recognized as important for rapid clearance of blood-borne ligands and maintenance of lipid homeostasis. Between 2012 and 2015, it was progressively established that LSECs contribute to the activation of HSCs and cause Kupffer cells to polarize into pro-inflammatory phenotypes. Single-cell RNA sequencing of the human liver in 2019 revealed three distinct groups of endothelial cells, laying the groundwork for further subgroup studies and functional improvement of the hepatic endothelium. In 2021 and 2022, studies further linked LSEC adhesion programs to MASH progression and capillarization to HCC-associated vascular remodeling. In 2023, Oit3 was identified as a promising LSEC-enriched marker and genetic entry point for studying LSEC biology

Liver sinusoidal endothelial cells (LSECs) line hepatic sinusoidal vasculature, accounting for approximately 15%–20% of the total hepatic cell population but only about 3% of total liver volume. Their open sieve-plate fenestrae and poorly organized basement membrane facilitate efficient exchange of oxygen, nutrients, lipoprotein remnants, and circulating solutes between sinusoidal blood and hepatocytes, distinguishing LSECs from continuous vascular endothelia in other organs [1]. Functional breakthroughs in the late 1990s and early 2000s revealed LSECs as the principal source of hepatic nitric oxide (NO), thereby governing sinusoidal tone [2, 3]. Crucially, LSECs function as non-professional antigen-presenting cells that process blood-borne antigens and shape antigen-specific T-cell activation and tolerance [4–6]. Beyond their distinctive morphology, LSECs actively regulate the sinusoidal microenvironment by controlling vascular tone, immune quiescence, endocytic clearance, coagulation-related responses, and angiocrine crosstalk with hepatocytes, hepatic stellate cells (HSCs), Kupffer cells, and other neighboring cells [7–9]. Over the past several decades, the understanding of LSEC biology has evolved substantially—from their initial characterization as a passive fenestrated endothelium to the recognition of their active roles in immune modulation, vascular homeostasis, and disease pathogenesis (Fig. 1). Recognition of capillarization provided an early mechanistic link between LSEC dedifferentiation and chronic liver disease progression, as LSECs promote fibrogenesis by activating HSCs and polarizing Kupffer cells toward pro-inflammatory phenotypes [10, 11]. Recent advances in single-cell transcriptomics have uncovered unappreciated heterogeneity within LSECs. Oit3 has emerged as a promising LSEC-enriched genetic marker and lineage-tracing entry point, although its expression and labeling specificity may vary across physiological states, lobular zones, and disease contexts [12]. Importantly, these technologies do more than classify endothelial subsets. They reveal how spatial position determines LSEC vulnerability, angiocrine output, immune interactions, and disease-stage-specific plasticity. These advances, together with spatial transcriptomic analyses, have shifted the field from viewing LSECs as a homogeneous endothelial barrier to recognizing them as zonally specialized and disease-responsive regulators of hepatic homeostasis.

Despite these anatomical and transcriptomic advancements, contemporary evidence demands a fundamental reframing of LSEC biology. LSECs are not merely passive bystanders responding to parenchymal injury; rather, they function as important sensors and integrators of metabolic, inflammatory, and hemodynamic signals [13]. Positioned at the blood–parenchyma interface, they continuously sample circulating signals and coordinate angiocrine and immune outputs that regulate hepatocyte metabolism, HSC quiescence, and immune tolerance [13, 14]. Their scavenger function also contributes to hemoglobin clearance [15]. Under homeostatic conditions, this rheostat function maintains sinusoidal patency and zonal specialization [16]. However, when the cumulative burden of hepatic stress exceeds a threshold, LSECs undergo a canonical phenotypic switch characterized by defenestration, capillarization, loss of vasoprotective NO signaling, and gain of pro-inflammatory adhesion molecules [17]. Notably, such an endothelial phenotypic shift is not merely a passive correlate of hepatic disorders. Across diverse liver pathologies, it arises as an early, therapeutically targetable endothelial perturbation driving the onset, exacerbation, and persistence of parenchymal injury, immune dysregulation, and fibrogenesis [18, 19].

To comprehensively illustrate this unifying framework, this review is organized to progress from baseline physiology to clinical therapeutics. First, the physiological roles of LSECs in maintaining hepatic equilibrium are outlined, focusing on their scavenger functions, metabolic regulation, and angiocrine signaling. Subsequently, the specific molecular mechanisms—primarily the transcriptional and epigenetic networks—that govern the LSEC phenotype are dissected. Building upon this mechanistic foundation, the pathophysiological roles of LSECs are then critically analyzed across a broad spectrum of etiologies, including acute liver injury (such as ischemia–reperfusion injury and drug-induced liver injury [6]), chronic liver disease (including metabolic dysfunction-associated steatotic liver disease, alcoholic steatohepatitis, and chronic hepatotoxic injury [20]), bacterial and viral infections, and hepatocellular carcinoma (HCC) [21]. Finally, current therapeutic strategies affecting or targeting LSECs are critically reviewed, addressing existing technological barriers and positioning therapeutic reprogramming—restoring the fully differentiated, tolerogenic LSEC phenotype—as the next transformative frontier in liver disease management. Concurrently, the absence of truly LSEC-specific Cre-driver lines and surface markers is explicitly acknowledged to be a major technical limitation in the field. Given that commonly used endothelial or scavenger receptor markers are shared with other vascular, lymphatic, or myeloid populations, many causal claims regarding LSEC-specific functions require further validation using more precise genetic and spatial tools.

## Physiology of LSECs

The unique anatomical and structural attributes of LSECs—specifically the absence of an organized basement membrane and the presence of open fenestrae organized into sieve plates—endow them with an exceptional capacity to act as integrative hubs within the hepatic sinusoid. LSECs are exquisitely positioned to simultaneously monitor portal and systemic blood constituents, sense hemodynamic forces, and communicate bidirectionally with hepatocytes, HSCs, and immune cells. This strategic anatomical position enables LSECs to serve as central regulators of hepatic homeostasis. These cells convert shifts in metabolic burden, inflammatory signaling molecules, and mechanical tension into synchronized cellular reactions, maintaining hepatic homeostasis under physiological conditions but promoting disease progression when regulatory control fails. In this section, the multidimensional homeostatic functions of LSECs are systematically reviewed. Their robust scavenger capabilities and role in metabolic regulation are first explored, followed by their control of vascular tone and paracrine angiocrine signaling. Finally, the zonal partitioning and disease-specific specialization of these functions are examined.

### Scavenger function and endocytic receptors

LSECs display exceptionally high endocytic and scavenging capacity, enabling rapid clearance of gut-derived microbial products, circulating debris, modified lipoproteins, and macromolecular ligands [22]. This function is mediated by several receptors preferentially expressed by LSECs, including stabilin-1 (STAB1), stabilin-2 (STAB2), mannose receptor/CD206 (MR/CD206), Fc gamma receptor IIb/CD32b (FcγRIIb/CD32b), lymphatic vessel endothelial hyaluronan receptor 1 (LYVE1), scavenger receptor class B type 1 (SR-B1), and scavenger receptor class F member 1 (SCARF1) [23]. Notably, despite being highly enriched on LSECs, most of these molecules are not absolutely specific. For instance, CD206 is also expressed by macrophages and lymph node endothelial cells, and LYVE1 is present on lymphatic endothelium.

**Fig. 2:**
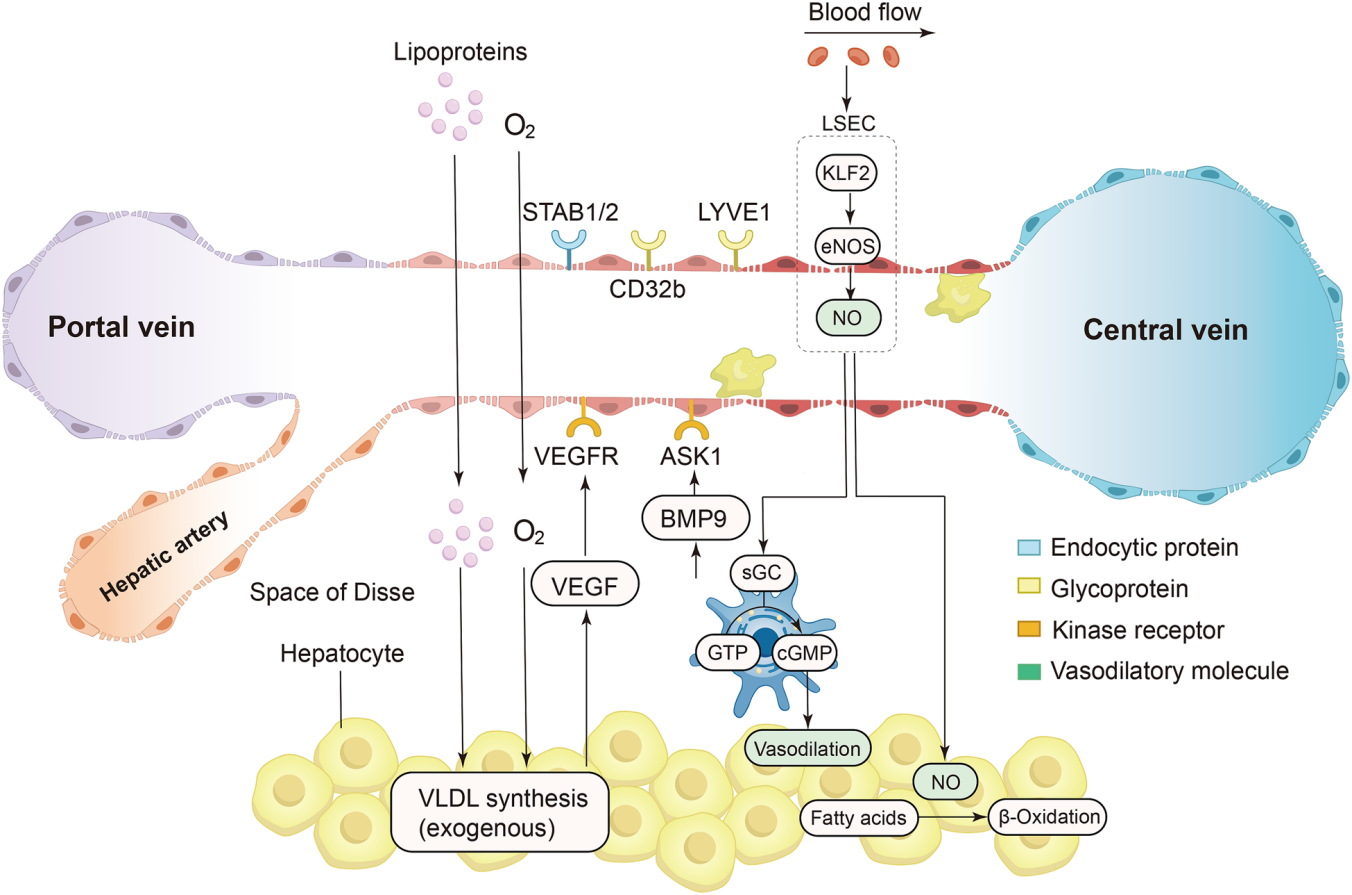
LSEC-mediated mechanisms preserving hepatic homeostasis. In the healthy sinusoid, LSECs display high scavenger-receptor activity, including STAB1/2, LYVE1, and FcγRIIb/CD32b, while maintaining fenestrae, NO production, and bidirectional exchange with hepatocytes. The absence of an organized basement membrane and the presence of fenestrae—transcellular pores arranged in sieve plates—permit direct bidirectional exchange between sinusoidal blood and hepatocytes. Through these structural openings, oxygen, micronutrients, and macronutrients (including lipoproteins in the form of chylomicrons) traverse the sinusoidal wall, thereby promoting hepatic VLDL synthesis and secretion. Hemodynamic shear stress activates the transcription factor KLF2, which in turn upregulates eNOS expression and drives NO synthesis. As a potent vasodilator, NO stimulates sGC to generate cGMP, thereby inducing sinusoidal vascular relaxation. Concurrently, NO maintains HSC quiescence and facilitates fatty acid β-oxidation within hepatocytes

These receptors are densely clustered on the luminal aspect of the sinusoidal endothelium, poised for efficient ligand capture. STAB1/2, LYVE1, and CD32b are prominently displayed on the luminal surface of LSECs and positioned to intercept blood-borne ligands (Fig. 2). STAB2 mediates the clearance of hyaluronan, chylomicron remnants, and advanced glycation end-products, while CD32b facilitates the uptake of IgG immune complexes. The mannose receptor mediates endocytic uptake of glycoproteins with terminal mannose residues, facilitating clearance of bacterial derivatives and foreign antigens [24]. Notably, these scavenger receptors exhibit relative zonal heterogeneity. While CD32b, LYVE1, and STAB2 are broadly expressed as canonical pan-LSEC markers across sinusoidal zones, Zone 2/3 (pericentral) LSECs show higher expression than Zone 1 (periportal) LSECs, which instead display enriched expression of factor VIII (F8) and platelet endothelial cell adhesion molecule-1 (PECAM1). This graded zonal distribution optimizes scavenger efficiency along the sinusoidal blood flow gradient [25].

### Maintenance of hepatocellular homeostasis and lipid metabolism

LSECs constitute the primary gateway governing bidirectional exchange between the sinusoidal blood and the hepatic parenchyma, exerting pivotal regulatory effects on hepatocyte homeostasis and lipid turnover [26]. The unique fenestrated architecture of LSECs—lacking a basement membrane and organized into sieve plates—enables the efficient transfer of lipoproteins, chylomicron remnants, oxygen, and nutrients across the Space of Disse to hepatocytes [1].

Lipoproteins and oxygen (O₂) traverse LSEC fenestrations to reach hepatocytes, where they are involved in very low-density lipoprotein (VLDL) synthesis and β-oxidation of fatty acids, respectively (Fig. 2). LSECs actively regulate lipid transfer via their high endocytic capacity and maintain hepatic lipid homeostasis by clearing acetylated low-density lipoprotein (acLDL), immune complexes, and cellular debris [27]. Accordingly, the integrity of LSEC fenestrae is essential for normal hepatocyte lipid metabolism [28]. As fenestrae provide the main route for lipid and nutrient exchange across the Space of Disse, their loss directly impairs hepatocyte lipid handling. In MASLD/MASH, such an impaired exchange can reduce VLDL secretion, compromise fatty acid oxidation, and promote steatosis prior to the onset of overt hepatocyte damage and HSC activation [29].

Beyond its impact on lipid metabolism, capillarization drives a wide range of pathological alterations. First, the loss of fenestrae and the deposition of a subendothelial basement membrane transform the discontinuous sinusoidal endothelium into a continuous capillary-like barrier, physically impeding the bidirectional exchange of oxygen, macromolecules, metabolites, and waste products between blood and hepatocytes across the Space of Disse [26]. Second, capillarized LSECs undergo a phenotypic switch from immune tolerance to pro-inflammation. This transition is characterized by upregulation of adhesion molecules, such as intercellular adhesion molecule 1 (ICAM1), vascular cell adhesion molecule 1 (VCAM1), and E-selectin, as well as secretion of chemokines, such as C-X-C motif ligand (CXCL)1, CXCL16, and CCL2, that recruit neutrophils, monocytes, and lymphocytes [30]. Concurrently, the downregulation of scavenger receptors (STAB1, STAB2, CD32b) diminishes the clearance of circulating pathogens and damage-associated molecular patterns (DAMPs), perpetuating intrahepatic inflammation. Third, by suppressing endothelial nitric oxide synthase (eNOS) and NO bioavailability, capillarized LSECs release the brake on HSC quiescence and actively secrete profibrogenic mediators, including transforming growth factor-β (TGF-β), platelet-derived growth factor (PDGF), and connective tissue growth factor (CTGF). These mediators directly stimulate HSC activation and extracellular matrix (ECM) deposition [31]. In turn, the newly deposited basement membrane components—such as laminin and collagen type IV—further increase sinusoidal stiffness, creating a mechanotransduction-mediated feedback loop that reinforces LSEC capillarization and sustains progressive fibrosis. Accordingly, LSEC capillarization represents a functional endothelial transition rather than merely a structural hallmark of chronic liver injury. This process impairs sinusoidal substance exchange, facilitates leukocyte recruitment, releases HSCs from NO-mediated restraint, and accelerates extracellular matrix deposition.

### Vascular regulation

LSECs are central regulators of hepatic microcirculatory tone and vascular homeostasis through the production of vasoactive mediators, particularly NO [32]. LSEC-derived NO activates soluble guanylate cyclase (sGC) in target cells, generating cyclic guanosine monophosphate (cGMP) and inducing vasodilation. Vascular endothelial growth factor (VEGF), primarily derived from hepatocytes and HSCs, reinforces this vasoprotective phenotype by maintaining LSEC fenestration and eNOS activity through VEGF receptor (VEGFR) signaling. Conversely, bone morphogenetic protein 9 (BMP9) secreted by HSCs preserves LSEC differentiation and prevents capillarization (Fig. 2) [33]. Dysregulation of the NO-cGMP signaling axis, induced by oxidative stress, diminished shear stress, or inflammatory cytokines, triggers LSEC defenestration, elevated vascular resistance, and subsequent portal hypertension [34].

### Paracrine signaling to hepatocytes and HSCs

LSECs communicate with neighboring cells through a sophisticated network of paracrine signals, collectively termed “angiocrine signaling,” which maintains sinusoidal homeostasis and orchestrates responses to injury [32]. LSECs regulate both hepatocyte function and HSC quiescence via these angiocrine signals.

LSEC-derived Wnt2 and hepatocyte growth factor (HGF) facilitate hepatocyte proliferation, metabolic zonation, and liver regeneration, while NO released from LSECs supports hepatocyte fatty acid β-oxidation (Fig. 2) [35, 36]. Under homeostatic conditions, healthy LSECs maintain HSC quiescence through NO-dependent signaling. The resulting cGMP production prevents HSC activation and can promote reversion of activated HSCs to a quiescent state. Conversely, HSC-derived BMP9 sustains LSEC differentiation, thereby maintaining a homeostatic feedback circuit that is disrupted during hepatic fibrosis. Dysfunctional LSECs also secrete profibrotic factors that further drive HSC activation and ECM deposition, including endothelin-1 (ET-1), PDGF, and TGF-β [37]. Consequently, therapeutic reprogramming of these angiocrine signals offers a paradigm shift. Restoring LSEC-derived NO may help re-establish HSC quiescence, whereas Wnt2 and HGF primarily support hepatocyte zonation and regeneration.

Instead of relying on discrete pairwise cellular interactions, LSECs coordinate a multicellular sinusoidal network, in which endothelial signals are integrated with HSC activation and Kupffer-cell polarization within an angiocrine–immune–fibrotic axis [38]. Under homeostatic conditions, LSEC-derived NO helps maintain HSC quiescence, whereas Wnt2 and HGF support hepatocyte metabolic zonation and regenerative capacity. LSECs also contribute to a tolerogenic sinusoidal immune environment, although the mediators governing Kupffer-cell polarization should be considered separately. During capillarization, myofibroblast activation, immune-cell recruitment, and inflammatory amplification are jointly promoted via loss of NO-mediated HSC restraint, increased secretion of PDGF, TGF-β, and CTGF, and upregulation of adhesion molecules such as ICAM1 and VCAM1 [39]. This axis forges the conceptual foundation for the disease sections below, where LSEC injury is interpreted as a disturbance coupling endothelial dysfunction, immune activation, and fibrogenic remodeling.

### Zonal heterogeneity and disease-specific functional reprogramming of LSEC subpopulations

The aforementioned zonal heterogeneity in receptor expression and scavenging capacity is dynamically regulated. Single-cell analysis of human cirrhosis demonstrates spatially organized scar-associated endothelial states, indicating that LSEC dysfunction is heterogeneous rather than uniform across diseased liver tissue [40]. In metabolic dysfunction-associated steatotic liver disease (MASLD/MASH) and metabolic dysfunction and alcohol-related liver disease (MetALD), pericentral Zone 2/3 LSECs presenting the highest scavenger activity under homeostatic conditions are preferentially subjected to lipotoxic injury and oxidative stress, rendering them the first subpopulation to undergo defenestration and capillarization [41]. This zonal susceptibility is mechanistically rooted in three converging gradients: First, pericentral LSECs reside at the lowest point of the hepatic oxygen gradient; the resulting relative hypoxia compromises mitochondrial oxidative phosphorylation and amplifies reactive oxygen species generation, accelerating endothelial dedifferentiation [42]. Second, because of their high scavenging capacity, Zone 2/3 LSECs are exposed to the greatest flux of oxidized low-density lipoproteins and free fatty acids transported across the sinusoid, directly inflicting lipotoxic membrane damage [39]. Third, pericentral hepatocytes are the primary site of lipogenic activity. Upon steatosis onset, these hepatocytes release lipotoxic extracellular vesicles and lipid peroxidation products into the space of Disse, creating a zone-restricted microenvironment that selectively overwhelms the antioxidant defenses of adjacent LSECs. Conversely, periportal Zone 1 LSECs, enriched in pro-coagulant factors (F8, vWF, PECAM1), exhibit exaggerated leukocyte adhesion and pro-thrombotic properties upon inflammatory stimulation, thereby driving periportal necro-inflammation and microvascular dysfunction (Fig. 2) [25]. Even within this progressively capillarized microenvironment, mid-zone LSEC-derived Wnt2 signaling continues to regulate hepatocyte metabolic zonation. This further underscores the heterogeneous, subset-specific nature of zonal functional impairment.

Beyond zonal vulnerability, single-cell studies have identified disease-associated LSEC states during MASH progression [41]. In this context, c-Kit should be interpreted primarily as a marker of a distinct LSEC subpopulation rather than as a validated pharmacological target. Separately, endothelial RAP1A restrains RAF1-mediated Notch activation and thereby limits LSEC capillarization [43]. c-Kit⁺ LSECs were reduced in experimental NASH and human NASH samples, and experimental restoration of c-Kit⁺ endothelial cells was associated with reduced steatosis, inflammation, and fibrosis and with enhanced PINK1-associated mitophagy [41, 44]. These findings suggest that loss of the c-Kit⁺ endothelial program may accompany impaired endothelial homeostasis; however, its lineage stability, causal contribution, and therapeutic tractability remain to be established. Neither imatinib nor masitinib should currently be regarded as LSEC-selective interventions. In experimental liver-fibrosis models, the best-supported antifibrotic mechanism of imatinib involves inhibition of PDGFR-β signaling in activated HSCs rather than selective pharmacological targeting of c-Kit⁺ LSECs [45, 46]. Direct in *vivo* evidence demonstrating selective suppression or depletion of the c-Kit⁺ LSEC subpopulation by either agent remains lacking.

These observations highlight a key interpretive caveat: LSEC-related biomarkers require context-based categorization instead of blanket classification as general injury indicators. In cirrhosis, single-cell analyses have identified a disease-specific scar-associated endothelial cell (SAEC) population marked by plasmalemma vesicle associated protein (PLVAP) and atypical chemokine receptor 1 (ACKR1) positivity [39]. These cells localize to fibrotic septa, lose classical LSEC-enriched markers (LYVE1, STAB2), and acquire a pro-fibrogenic angiocrine signature characterized by elevated secretion of TGF-β and CTGF, thereby promoting myofibroblast activation. The emergence of PLVAP/ACKR1⁺ SAECs has important translational implications. PLVAP⁺ and ACKR1⁺ endothelial populations are enriched within fibrotic septa and scar-associated niches and show reduced canonical LSEC programs together with signatures linked to leukocyte trafficking and fibrotic-niche remodeling; independently, RAP1A–RAF1–Notch signaling can reinforce endothelial capillarization during fibrosis [43]. These markers delineate a markedly dedifferentiated scar-associated endothelial state. The loss of scavenger receptors, together with increased profibrogenic secretion, supports the view that SAECs are active participants rather than passive bystanders in septal fibrogenesis. The restricted enrichment of PLVAP in scar-associated endothelial populations suggests potential value as a spatial biomarker and candidate delivery target; however, direct evidence supporting PLVAP-targeted cell depletion in liver fibrosis remains limited. ACKR1-associated chemokine trafficking may contribute to immune-cell recruitment within fibrotic septa, although its therapeutic inhibition requires direct validation. This suggests a dual-target approach against both the vascular and inflammatory components of cirrhosis. Thus, PLVAP/ACKR1 dual positivity links disease classification with mechanism and therapy. It characterizes advanced scar-related endothelial remodeling, clarifies the mechanism by which endothelial cells maintain septal fibrogenesis, and presents a spatially specific target for antifibrotic intervention.

Their preferential localization to fibrotic septa is consistent with active reprogramming of resident LSECs by the scar microenvironment [47]. Spatial analyses have indicated that the stiffened extracellular matrix within septa generates mechanical stress, downregulating the endothelial master regulator ETS-related gene (ERG) while upregulating Yes-associated protein/transcriptional coactivator with PDZ-binding motif (YAP/TAZ) signaling to prime a mesenchymal-like, pro-fibrogenic phenotype. Concurrently, disorganized neovasculature creates localized hypoxia that stabilizes hypoxia-inducible factor 1-alpha (HIF-1α), while adjacent activated HSC secrete TGF-β and Notch ligands. These microenvironmental cues may contribute to the loss of canonical LSEC identity and the acquisition of a scar-associated endothelial phenotype. In particular, the senescence-associated secretory phenotype has been shown to upregulate PLVAP in human hepatic endothelial cells and promote PLVAP-dependent monocyte transmigration [48]. Converging on resident sinusoidal endothelium, these distinct microenvironmental insults collectively suppress canonical LSEC identity and promote a scar-associated angiocrine program, thereby favoring the emergence of SAECs in regions with robust mechanical, hypoxic, and biochemical pathological stimuli.

Collectively, from pericentral and periportal zonal subsets in MASH to scar-reprogrammed SAECs in cirrhosis, these disease-specific LSEC subpopulations demonstrate that the functional impact of LSECs in liver disease is dictated by their spatial identity and disease-stage-specific plasticity. In HCC, this vulnerability is further reshaped by the tumor microenvironment, where chronic hypoxia, VEGF overload, TGF-β-rich stromal signaling, and immune checkpoint induction drive tumor-associated LSECs toward angiogenic, capillarized, and immunosuppressive states. Accordingly, subpopulation-aware therapeutic strategies and zone-specific reprogramming approaches that restore the scavenger and tolerogenic identity of diseased LSEC subsets are increasingly important. Notably, recently identified LSEC-associated markers should be interpreted according to their biological context rather than grouped uniformly as damage-associated markers. Oit3 appears to mark quiescent or homeostatic LSEC identity and may serve as a genetic entry point for studying LSEC biology. By contrast, PLVAP/ACKR1 and S100A16 are associated with disease-specific endothelial remodeling, whereas loss or reduction of the c-Kit⁺ endothelial program may accompany progressive steatohepatitis and impaired endothelial homeostasis. PLVAP/ACKR1⁺ scar-associated endothelial cells identify fibrotic niches in cirrhosis, and S100A16⁺ tumor-associated endothelial cells, identified in human HCC and functionally evaluated in murine models, reflect vascular–immune remodeling in HCC. Thus, these markers are not merely descriptive labels. Instead, they represent functional disease coordinates that explain the LSEC state, spatial niche, disease stage, pathogenic mechanism, and potential therapeutic accessibility. Understanding these regulatory networks is essential for defining the critical physiological roles and spatial heterogeneity of LSECs in maintaining hepatic equilibrium. Therefore, specific molecular mechanisms that govern these dynamic phenotypic states are subsequently explored.

## Molecular mechanisms governing LSEC phenotype and function

The phenotypic plasticity of LSECs is controlled by transcriptional, epigenetic, receptor-mediated, and mechanometabolic programs. Under homeostatic conditions, these programs maintain fenestration, scavenger capacity, NO production, and immune tolerance. Under persistent metabolic, inflammatory, or mechanical stress, the same regulatory circuits are rewired toward capillarization, eNOS suppression, inflammatory adhesion, and profibrotic angiocrine signaling. Accordingly, this section outlines the core molecular pathways sustaining LSEC identity and explains how their disruption promotes disease-specific endothelial remodeling.

### Transcriptional regulation

The specialized phenotype of LSECs is governed by a distinct transcriptional program that distinguishes them from vascular endothelial cells in other organs [49]. Indeed, comparative transcriptome profiling of liver, heart, and brain endothelial cells has established a 30-gene LSEC signature comprising 23 LSEC-enriched genes and 7 transcription factors (TFs), including musculoaponeurotic fibrosarcoma (C-MAF), GATA binding protein 4 (GATA4), MEIS homeobox 2 (MEIS2), zinc finger E-box-binding protein 2 (ZEB2), homeobox B5 (HOXB5), Cut-like homolog 2 (CUX2), and transcription factor EC (TCFEC) [49].

Among these TFs, C-MAF, GATA4, and MEIS2 have emerged as the strongest inducers of LSEC marker expression. When overexpressed individually in human umbilical vein endothelial cells (HUVECs), these TFs upregulate key LSEC-enriched markers including mannose receptor C-type 1 (MRC1), liver/lymph node-specific ICAM-3 grabbing non-integrin (L-SIGN/CLEC4M), and legumain (LGMN). Notably, the triple combination of C-MAF, GATA4, and MEIS2 produces synergistic effects, inducing 43% of LSEC signature genes by at least twofold. The combined transcription factor treatment increases the protein levels of L-SIGN, MRC1, pro-LGMN, GPR182, PLXNC1, and SLCO2A1 and enhances cellular binding affinity to the E2 protein of the hepatitis C virus. However, this combination of transcription factors does not induce endocytosis via scavenger receptors or the appearance of fenestrae, suggesting that further regulatory signals are necessary to fully equip LSECs with their functional properties [49].

Krüppel-like factor 2 (KLF2) represents another critical transcriptional regulator of LSEC phenotype. Flow-derived shear stress induces KLF2 expression, which subsequently upregulates eNOS to maintain the fenestrated, differentiated state. KLF2 also suppresses pro-inflammatory and pro-adhesive genes, conferring vasoprotective properties. In cirrhotic livers, KLF2 expression is reduced, contributing to LSEC capillarization. Conversely, KLF2 overexpression restores LSEC differentiation, ameliorates endothelial dysfunction, and promotes fibrosis regression [50].

As a zinc-finger E-box-binding homeobox transcription factor enriched in LSECs, ZEB2 preserves liver angioarchitecture and prevents fibrosis by maintaining LSEC identity. Endothelial-specific GATA4 deletion leads to spontaneous liver fibrosis through a GATA4-MYC-PDGFB axis. This finding further underscores the important role of this TF in LSEC-mediated hepatic homeostasis. During liver development, c-MAF induction is required for the specification of fetal liver endothelial progenitors into functional zonated adult sinusoids [51, 52]. These transcriptional networks therefore constitute druggable hubs for LSEC reprogramming. Therapeutic reactivation of KLF2 or GATA4 may help reverse capillarization and restore functional fenestration. Such transcription factor-targeted strategies may ultimately re-establish the sinusoidal gatekeeper phenotype in chronic liver disease.

### Epigenetic modifications in LSEC differentiation and maintenance

Epigenetic mechanisms, including DNA methylation, histone modifications, and non-coding RNA regulation, play fundamental roles in establishing and maintaining LSEC identity. Genome-wide methylome and transcriptome analyses of purified human liver cells have identified cell type-specific epigenetic signatures in hepatocytes, LSECs, and HSCs [53]. While most current evidence centers on HSC activation, emerging LSEC-targeted studies further demonstrate that Airn/EZH2-dependent regulation of the KLF2–eNOS–sGC pathway and mechanotransduction-induced glycolytic remodeling contribute directly to LSEC dedifferentiation, capillarization, and pro-fibrotic angiocrine reprogramming [30, 54].

Long non-coding RNAs (lncRNAs) represent a critical layer of epigenetic regulation in LSECs. The lncRNA Airn interacts with enhancer of zeste homolog 2 (EZH2), a core component of the polycomb repressive complex 2 (PRC2), to maintain LSEC differentiation via the KLF2-eNOS-sGC pathway. Airn knockdown in primary LSECs reduces KLF2 and eNOS expression, induces laminin upregulation, and promotes capillarization. Conversely, Airn overexpression preserves LSEC fenestration while preventing HSC activation. Mechanistically, Airn functions as a molecular scaffold that recruits EZH2 to specific genomic loci, thereby modulating histone H3 lysine 27 trimethylation patterns that govern LSEC-specific gene expression [54].

MicroRNAs also facilitate LSEC epigenetic regulation. miR-27b-3p modulates liver sinusoidal endothelium dedifferentiation in chronic liver disease, with dysregulated levels correlated with established capillarization biomarkers [55]. Additionally, miR-511-3p promotes Hedgehog signaling by targeting Patched-1, thereby shaping LSEC phenotype in hepatic sinusoidal obstruction syndrome [55, 56].

The mechanical microenvironment further affects the LSEC epigenetic landscape. Triggered by increased sinusoidal stiffness in fibrotic livers, mechanotransduction-induced glycolysis epigenetically reprograms LSECs toward a pro-angiogenic, pro-fibrotic phenotype through metabolic-epigenetic coupling and altered histone acetylation patterns [30]. Stiffness-induced modulation of the ERG transcription factor, a master regulator of endothelial identity, has also been implicated in chronic liver disease-associated LSEC dysfunction [57]. Taken together, these epigenetic insights reveal that LSEC dedifferentiation is not terminal but reversible, opening the door to epigenetic reprogramming strategies that reset endothelial identity. Targeting mechano-epigenetic coupling or lncRNA-mediated chromatin remodeling may thus be positioned as precise therapeutic modalities to durably restore the quiescent LSEC phenotype.

### VEGF-BMP9-Notch signaling and mechanometabolic regulation of LSEC phenotype

Beyond transcriptional and epigenetic mechanisms, multiple additional molecular pathways such as post-translational modifications, metabolic reprogramming, and intercellular signaling networks govern LSEC phenotype and function.

The VEGF-A/VEGFR2 axis is important for LSEC phenotype maintenance. LSECs express VEGFR2 and respond to VEGF-A derived primarily from hepatocytes and HSCs. This dual behavior constitutes a central VEGF paradox in LSEC biology. Under physiological conditions, transient VEGF-A-VEGFR2 signaling activates Akt/AMPK-dependent eNOS phosphorylation and NO production, thereby preserving LSEC fenestration [58, 59], where eNOS refers to the total endothelial nitric oxide synthase protein, whereas p-eNOS denotes its phosphorylated and enzymatically activated form. Phosphorylation of eNOS at Ser1177 generates p-eNOS, which directly catalyzes NO production. Accordingly, eNOS is used here to denote total enzyme expression or the KLF2–eNOS–NO signaling axis, whereas p-eNOS denotes the activated phosphorylated form. During chronic injury, altered ligand abundance, exposure duration, receptor balance, hypoxia, and inflammatory signaling may disrupt the physiological VEGF–eNOS response and contribute to capillarization. However, the relative contributions of VEGFR1, VEGFR2, ERK activation, and eNOS uncoupling in diseased LSECs remain incompletely defined. VEGF should therefore be interpreted as a context-dependent regulator rather than as an intrinsically protective or pathogenic factor [3]. Secreted by HSCs, BMP9 represents a circulating endothelial quiescence factor that maintains LSEC terminal differentiation markers (LYVE1, STAB1/2, Ehd3, Cd209b, eNOS, MAF, PLVAP). Reduced fenestration, increased basement membrane deposition, and spontaneous liver fibrosis are observed in BMP9 knockout mice [33, 60].

The Notch signaling pathway exerts context-dependent effects on LSEC phenotype. Mediated by Delta-like ligand 4 under hypoxic conditions during liver fibrosis, endothelial Notch activation promotes LSEC dedifferentiation and capillarization through eNOS-sGC signaling inhibition. Endothelial Notch activation reshapes sinusoidal angiocrine signaling, promotes capillarization and fibrosis, and blunts liver regeneration [61].

LSECs rely substantially on oxidative phosphorylation and display limited mitochondrial oxidation of long-chain fatty acids [62]. The contribution of glycolysis and short-chain fatty-acid metabolism to specific LSEC functions should be described only where supported by direct LSEC evidence. Triggered by increased sinusoidal stiffness in fibrotic livers, mechanotransduction-induced glycolysis epigenetically reprograms LSECs toward a pro-angiogenic, pro-fibrotic phenotype through altered histone acetylation patterns [30].

Collectively, these interactions illustrate the bidirectional communication between LSECs and neighboring cells. HSC-derived BMP9 and hepatocyte/HSC-derived VEGF-A maintain LSEC differentiation, while LSEC-derived NO preserves HSC quiescence via the sGC-cGMP pathway [63]. Concurrently, LSECs also regulate hepatocyte function through Wnt2 and HGF secretion, thereby promoting metabolic zonation and regeneration. Disruption of reciprocal VEGF-A/NO, profibrotic, and mechanotransduction signals contributes to coordinated failure of sinusoidal homeostasis in chronic liver disease [64]. Ultimately, disrupting these finely tuned molecular and epigenetic circuits drives the transition from homeostasis to pathology. Existing insights into liver disease-associated molecular abnormalities enable the identification of plausible therapeutic candidates for clinical translation.

## Pathophysiological roles of LSECs in liver diseases

**Fig. 3:**
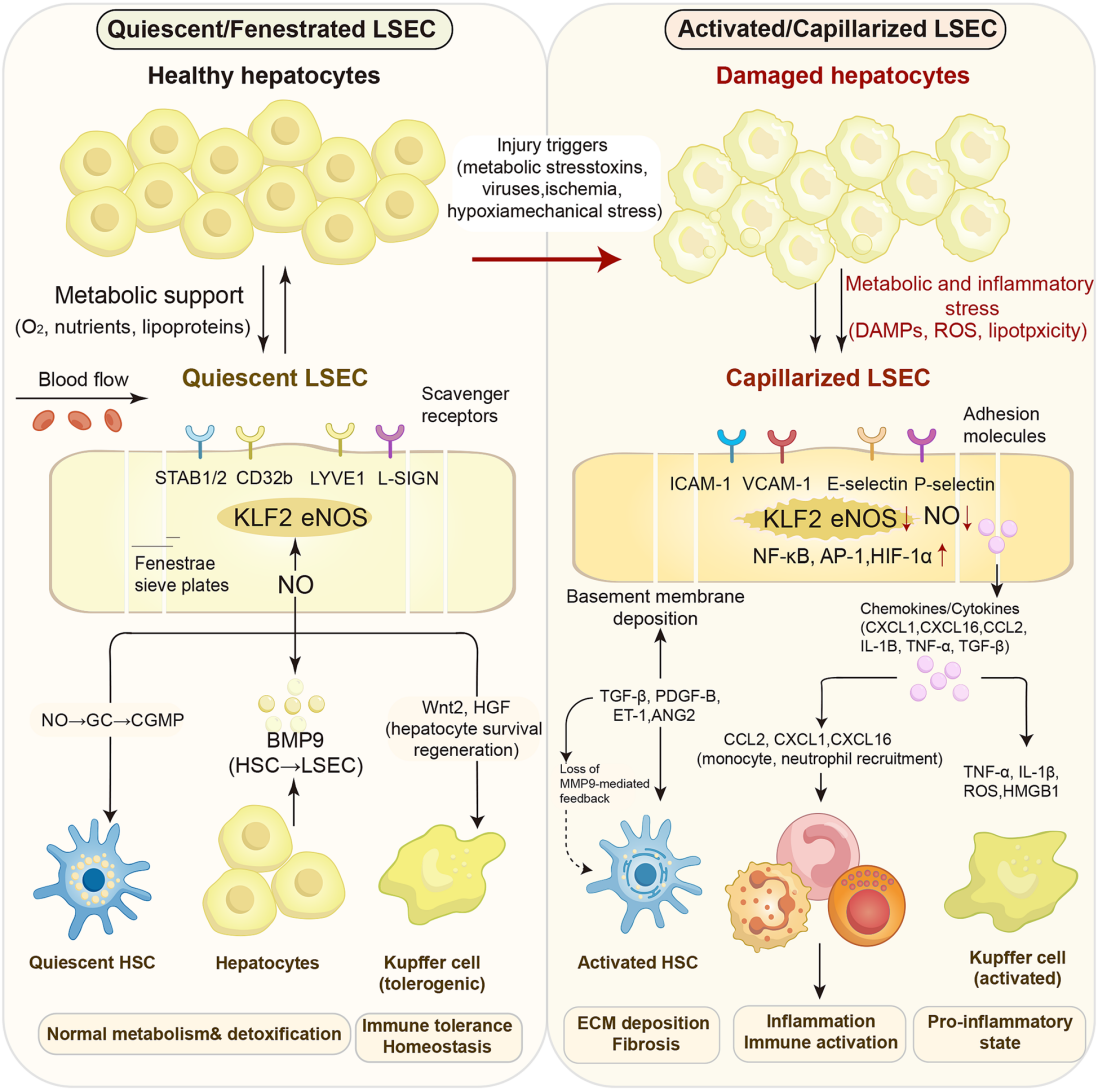
Transition from quiescent to capillarized LSECs during liver injury. LSECs undergo a phenotypic switch from a quiescent, fenestrated, NO-producing, and tolerogenic state to an activated, capillarized, pro-inflammatory, pro-fibrotic, and pro-thrombotic state. Diverse upstream insults, including metabolic stress, toxins, viral infection, ischemia, hypoxia, and mechanical stress, converge on LSECs and trigger loss of fenestration, basement membrane deposition, suppression of KLF2-eNOS-NO signaling, and induction of adhesion molecules and chemokines. These endothelial changes promote HSC activation, Kupffer cell inflammatory polarization, immune cell recruitment, and fibrosis

**Fig. 4:**
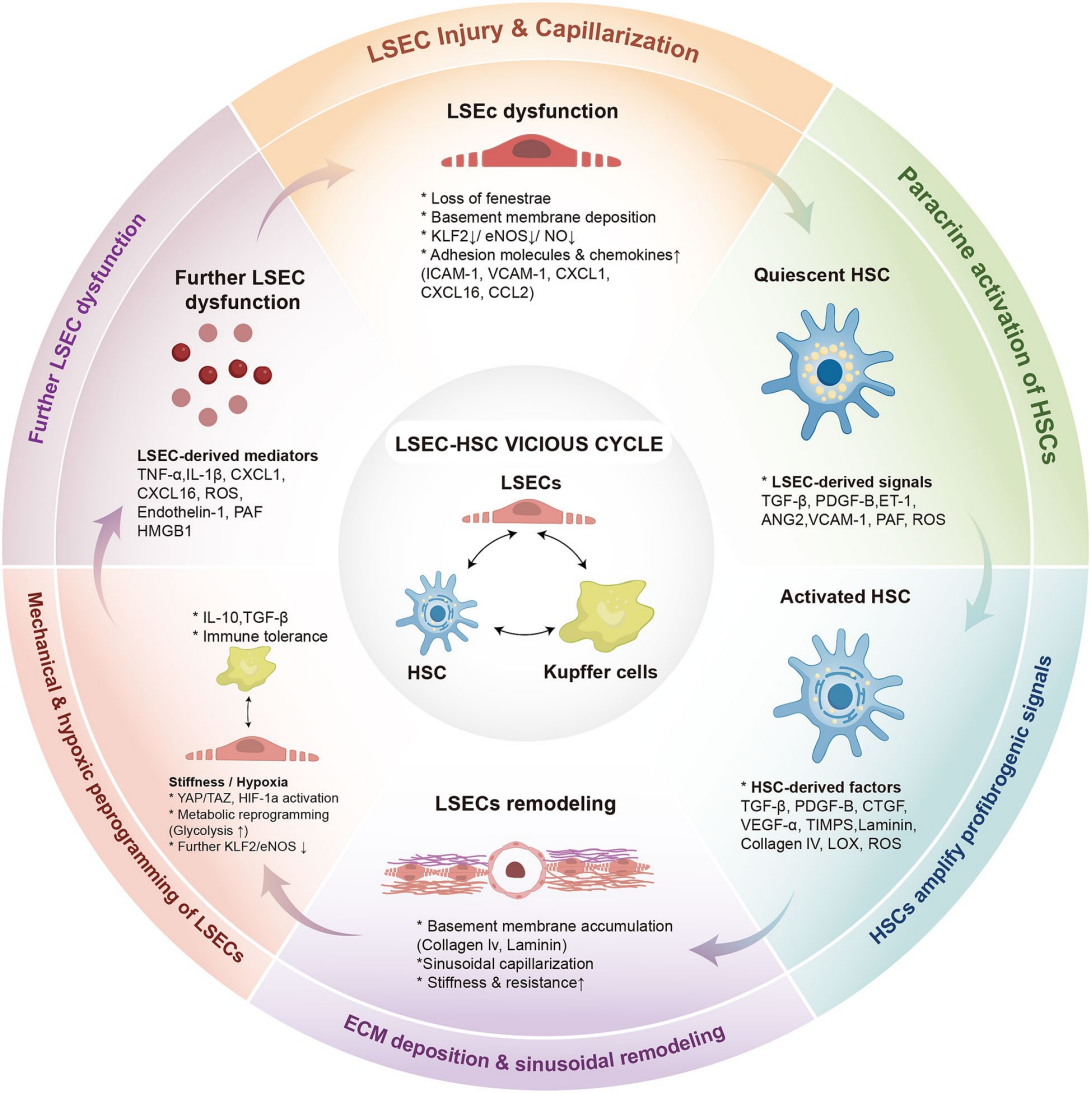
LSEC-centered angiocrine-immune-fibrotic vicious cycle. The self-amplifying pathological coupling among LSECs, HSCs, and Kupffer cells is a key factor in disease progression. LSEC injury and capillarization suppress NO signaling and promote profibrotic and inflammatory mediator release, leading to HSC activation and Kupffer cell inflammatory polarization. Activated HSCs deposit extracellular matrix and release profibrotic factors, increasing sinusoidal stiffness, hypoxia, and mechanical stress, which further suppress LSEC identity and reinforce capillarization. In parallel, activated Kupffer cells release TNF-α, IL-1β, ROS, and HMGB1, amplifying endothelial inflammation and completing the angiocrine-immune-fibrotic vicious cycle

LSEC dysfunction has long been interpreted as a downstream consequence of hepatocyte injury, HSC activation, or established fibrosis. However, accumulating evidence across acute liver injury, metabolic liver disease, viral hepatitis, cirrhosis, and HCC also suggests the active involvement of LSECs in disease amplification and progression. As schematically summarized in Figs. 3 and 4, LSEC dysfunction can be understood at two complementary levels: a local sinusoidal feed-forward circuit and a cross-disease endothelial injury program. At the local sinusoidal level, LSEC injury promotes HSC activation, Kupffer cell inflammatory polarization, and extracellular matrix deposition, while activated HSCs, inflammatory mediators, stiffness, and hypoxia further reinforce LSEC capillarization. This positive-feedback cascade helps explain how activation of the angiocrine–immune–fibrotic axis sustains endothelial dysfunction. Across different liver diseases, diverse upstream insults converge on several recurrent features of LSEC dysfunction, including loss of fenestration, basement membrane deposition, suppression of KLF2-eNOS-NO signaling, reduced scavenger and tolerogenic capacity, and acquisition of inflammatory, fibrogenic, pro-thrombotic, or immunosuppressive angiocrine outputs. These shared alterations impair blood-parenchyma exchange, reduce clearance of DAMPs and microbial products, remove NO-mediated restraint on HSC activation, and facilitate leukocyte recruitment, Kupffer cell activation, ECM deposition, and fibrosis.

Meanwhile, the initiating triggers differ among diseases. Ischemia–reperfusion injury is dominated by hypoxia, ATP depletion, ROS generation, and microvascular stress [65]. Drug-induced liver injury involves toxic metabolites, hepatocyte-derived DAMPs, and inflammatory mediators [66]. MASLD/MASH [67] and MetALD [68] are driven largely by lipotoxicity, lipid peroxidation products, and zonation-dependent metabolic stress. Viral hepatitis involves chronic antigen exposure, pattern-recognition receptor activation, interferon signaling, and tolerance failure [69, 70]. Cirrhosis is shaped by stiffness, hypoxia, and scar-associated TGF-β/Notch signaling, and HCC imposes tumor-derived hypoxia, persistent VEGF signaling, stromal TGF-β, and immune-checkpoint pressure on the sinusoidal endothelium [30, 48].

Accordingly, subsequent disease chapters are structured around a shared-core and disease-specific-trigger framework. Rather than representing a uniform process or a passive outcome of hepatic injury, LSEC dysfunction constitutes a context-dependent endothelial response that drives parenchymal injury, immune remodeling, fibrosis, or tumor progression in a microenvironment-specific manner.

### LSECs in acute liver diseases

#### Ischemia–reperfusion injury

**Fig. 5:**
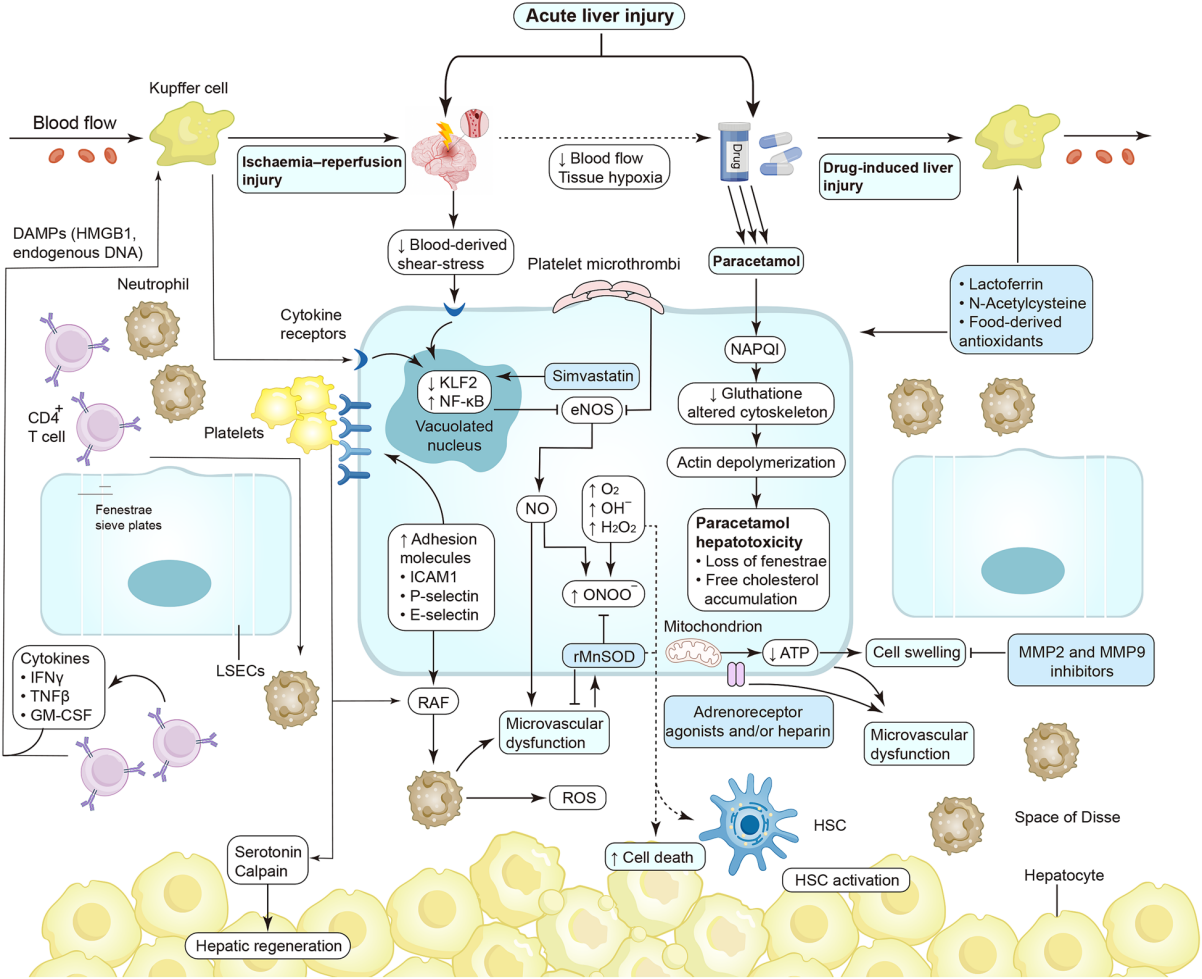
LSEC-mediated mechanisms in acute liver injury. (1) During ischemia–reperfusion injury, blood flow is interrupted, tissue hypoxia occurs, LSECs become rounded and undergo metabolic changes, and flow-derived shear stress is reduced. These changes reduce KLF2 expression in LSECs, which lowers eNOS expression. Simvastatin preserves endothelial homeostasis after ischemia–reperfusion damage by inducing KLF2 expression through the regulation of the small GTPase RAB7/RAC1. Furthermore, proinflammatory cytokines are secreted by Kupffer cells and ROS are produced by nearby neutrophils. These cells disrupt the microcirculation and recruit CD4^+^ T lymphocytes, which increase cytokine production and amplify inflammation. Additionally, hypoxia-induced DAMP release from hepatocytes provides feedback activation of Kupffer cells. The production of adhesion molecules in LSECs promotes platelet adherence and the development of vascular microthrombi in response to ischemia–reperfusion damage. LSEC-derived PAF promotes platelet activation, which can further stimulate neutrophils and increase ROS production. Platelets release regenerative mediators such as serotonin and growth factors. (2) In drug-induced liver injury, reactive metabolites such as paracetamol-derived N-acetyl-p-benzoquinone imine produce hepatotoxicity by reducing glutathione levels and depolymerizing actin in LSECs. Microcirculatory dysfunction is caused by these factors, as well as the buildup of free cholesterol in endolysosomes and the loss of fenestrae, which creates gaps and permits neutrophil and macrophage extravasation. In LSECs, mitochondrial injury contributes to cell swelling, whereas MMP inhibition may attenuate endothelial injury. α1-adrenoceptor antagonists and heparin can improve hepatic microcirculatory function during drug-induced liver injury. Potential downstream outcomes include loss of hepatocyte-specific functions, hepatocyte death, and HSC activation. Reproduced with permission from Ref. [35]. Copyright © 2021, Springer Nature Limited

Ischemia–reperfusion injury represents an acute example of LSEC rheostat failure, with abrupt hypoxia, ATP depletion, and ROS generation rapidly disrupting the KLF2-eNOS-NO axis and initiating inflammatory-endothelial coupling. Ischemia–reperfusion injury results from temporary interruption and subsequent restoration of hepatic blood flow during procedures such as liver transplantation or liver resection [71]. Multiple cellular and molecular pathways contribute to the resulting hepatic injury [72]. Along with hepatocytes, LSECs are key regulators during acute liver injury and are particularly vulnerable to ischemia–reperfusion injury. Both warm and cold ischemia–reperfusion injury can occur during organ procurement and transplantation, with distinct cell-type-specific effects. Hepatocytes sustain more profound damage upon warm ischemia compared with LSECs, whereas cold ischemia preferentially injures LSECs. Rodent experiments show roughly half of these endothelial cells become functionally compromised after 48 h of ischemic insult [73] (Fig. 5).

##### Hypoxia-induced LSEC injury

The first stage of ischemia–reperfusion damage arises from tissue hypoxia and insufficient blood supply to the organ, which rapidly disrupts the function of parenchymal and non-parenchymal liver cells [65]. During ischemia, rat LSECs become rounded, develop nuclear vacuolization, and display discontinuities in the plasma membrane. Concurrent with reduced tissue nitric oxide and elevated endothelin, thromboxane A_2_ (TXA_2_), and reactive oxygen species (ROS) generation, this condition is accompanied by metabolic perturbations and depleted adenosine triphosphate (ATP) availability, collectively altering LSEC phenotype and microvascular perfusion. The mechanosensing transcription factor KLF2, a main regulator of these processes in rat and human LSECs, is downregulated under no-flow conditions, and as a result, so are its derived transcriptional programs [74]. Indeed, KLF2 suppression during ischemia results in decreased expression and activity of endothelial eNOS, which in turn causes inadequate NO production, sinusoidal vasoconstriction, and increased liver vascular resistance during the reperfusion stage [75]. Moreover, hepatic reoxygenation during reperfusion markedly increases ROS generation, thereby aggravating LSEC dysfunction and sinusoidal microcirculatory injury [65, 76].

##### Immune cell–LSEC interactions

During ischemia–reperfusion injury, signals from activated Kupffer cells, infiltrating neutrophils and lymphocytes, injured hepatocytes, HSCs, and platelets compromise the homeostatic tolerogenic function of LSECs [76]. While hepatocytes and HSCs attempt to preserve the typical LSEC phenotype by producing VEGF [77], the prevailing inflammatory milieu overrides these homeostatic cues. Under liver injury, proinflammatory cytokines such as tumor necrosis factor (TNF) and interleukin (IL)−1, coupled with ROS released by activated Kupffer cells and neutrophils, trigger NF-κB activation in LSECs. This transcriptional response increases the expression of adhesion molecules, including P-selectin, intercellular adhesion molecule 1 (ICAM-1), and E-selectin, thereby promoting platelet adhesion and neutrophil recruitment and extravasation. Concurrently, by accelerating Kupffer cell activation and neutrophil migration via the production of interferon-γ, lymphotoxin-α, and granulocyte–macrophage colony-stimulating factor, recruited CD4⁺ T cells further amplify this inflammatory state [76].

Cellular damage also results in the release of DAMPs from injured liver cells, including high-mobility group box 1 (HMGB1) and endogenous DNA, facilitating positive feedback for Kupffer cell activation. From a translational standpoint, DAMPs, particularly HMGB1, fulfill a translational triad in acute liver injury. Elevated circulating HMGB1 levels correlate with the degree of hepatocellular necrosis and predict progression to acute and acute-on-chronic liver failure, enabling risk stratification beyond conventional aminotransferase profiles. Extracellular HMGB1 engages Toll-like receptor 4 (TLR4) and the receptor for advanced glycation end-products (RAGE) on LSECs and Kupffer cells, establishing a self-amplifying inflammatory loop that drives sinusoidal vasoconstriction, neutrophil recruitment, and coagulopathy. HMGB1-neutralizing antibodies, TLR4 antagonists, and pharmacological inhibitors of RAGE signaling have demonstrated preclinical efficacy in attenuating sterile inflammation and preserving LSEC fenestration, thereby positioning DAMP blockade as a disease-modifying strategy rather than merely supportive care.

Notably, the DAMP-driven LSEC–Kupffer-cell axis described here represents a shared pathogenic template recapitulated across acute and chronic liver diseases. Specifically, in MASH, IL-1R1-dependent inflammatory LSEC states can amplify chemokine-mediated immune-cell recruitment; in cirrhosis, PLVAP⁺/ACKR1⁺ scar-associated endothelial populations participate in fibrotic-niche remodeling; and in HCC, tumor-associated endothelial states can couple angiogenesis with immune suppression. Collectively, the acute DAMP axis centered on HMGB1 and the chronic maladaptive markers discussed below illustrate a unifying pathological template: LSEC phenotypic drift initiates self-propagating circuits that propel disease stage progression, and targeted disruption of these circuits enables stage-specific therapeutic intervention.

##### Platelet–LSEC interactions

In ischemia–reperfusion injury, platelets are a double-edged sword. On the one hand, hepatic platelet infiltration facilitates LSEC apoptosis by inducing hepatic microthrombosis and releasing platelet-activating factor. This factor, also secreted by LSECs, further augments neutrophil-derived local ROS production [78]. However, in murine models, platelets can also secrete multiple mediators—including calpain, NO, serotonin, and platelet-activating factor—to facilitate hepatic regeneration [10].

##### LSEC autophagy

Autophagy promotes the removal of damaged cellular material and participates in regulating ROS generation through lysosomal degradation [79]. Because agents that stimulate autophagy, such as the 3-hydroxy-3-methylglutaryl-coenzyme A (HMG-CoA) reductase inhibitor simvastatin, protect against ischemia–reperfusion injury in both non-steatotic and steatotic rat livers, it is proposed that autophagy may contribute to preventing liver dysfunction in ischemia–reperfusion injury. Simvastatin may preserve LSEC vasoprotective programs by preventing RAB7–KLF2 downregulation [75]. The therapeutic importance of LSECs in this context is further underscored by preclinical evidence from aged models, wherein statins confer vasoprotective effects on LSECs against ischemia–reperfusion injury [80]. Additionally, during ischemia–reperfusion injury, hypoxic episodes cause the injured liver to produce hypoxia-inducible factors, which in turn stimulate the transcription of genes that protect cells, including Hmox1, the gene encoding heme oxygenase-1 (HO-1) in mouse hepatocytes. HO-1 has been shown to protect LSECs in *vitro* by increasing their survival rate and decreasing proinflammatory cytokine levels [81].

##### LSEC-mediated liver regeneration

During liver damage, VEGF derived primarily from hepatocytes and HSCs stimulates the growth of bone marrow-derived sinusoidal progenitor cells. These cells can contribute to sinusoidal regeneration and differentiate into mature LSECs; they may also produce HGF, thereby supporting hepatocyte proliferation. Liver regeneration cannot be driven solely by functional mature LSECs [82].

Hepatocytes, Kupffer cells, and LSECs overproduce endocannabinoids, which activate the cannabinoid type 2 (CB2) receptor and may protect against liver ischemia–reperfusion injury while supporting regeneration. Both human and mouse LSECs express this receptor, and in *vitro* adherence of neutrophils to LSECs and TNF-induced ICAM1 expression are decreased by activation of this receptor with the agonist JWH133 [83]. Adipokines are cytokines mostly generated by adipose tissues. They have been linked to liver regeneration, particularly in human and animal models of ischemic steatotic livers [84]. While their exact biological roles remain incompletely defined, leptin, adiponectin, and especially IL-6 have been reported to facilitate hepatic regeneration following ischemia–reperfusion injury in mice [85].

Beyond the systemic metabolic and hormonal signals described above, LSECs influence liver regeneration through coordinated immune and angiocrine mechanisms. During the early phase of injury, increased expression of adhesion molecules such as VCAM1 and ICAM1 facilitates monocyte recruitment and inflammatory cell–endothelial interactions. Although these responses may support the initial clearance of damaged tissue, their persistence can amplify inflammation and fibrogenesis [86, 87]. In parallel, VEGF derived predominantly from hepatocytes and HSCs promotes sinusoidal endothelial survival and vascular reconstruction, whereas LSECs support hepatocyte and progenitor-cell responses through the release of angiocrine factors [87, 88]. This transient sinusoidal remodeling can establish a reparative vascular niche that facilitates tissue regeneration; however, persistent LSEC activation and pathological capillarization may instead impair parenchymal exchange and promote progressive fibrosis [89]. Therefore, therapeutic reprogramming of activated LSECs should aim to restore a differentiated, angiocrine-competent phenotype while limiting sustained inflammatory and profibrogenic signaling.

While arachidonic acid-derived TXA_2_ drives inflammation and platelet accumulation during acute hepatic injury, its receptor exerts beneficial effects on liver regeneration by facilitating macrophage recruitment [90]. These findings indicate that TXA2 signaling may have stage-dependent effects, warranting caution when considering pathway inhibition in the setting of liver surgery [91].

#### Drug-induced liver injury

Drug-induced liver injury refers to hepatic injury caused by medications, herbal products, or other xenobiotics [92]. The term “intrinsic drug-induced liver injury” refers to predictable and dose-related toxicity that occurs after sufficient exposure to a hepatotoxic agent [93]. This category includes predictable hepatotoxic reactions to agents such as paracetamol. In numerous countries, paracetamol intoxication is responsible for roughly half of acute liver failure incidents [94] (Fig. 5).

Drugs reach hepatocytes through sinusoidal blood and cross the basolateral membrane by passive diffusion or via transporters, including organic anion and cation transporters, prostaglandin transporters, and the Na^⁺^-taurocholate cotransporting polypeptide [95]. Therefore, even while drug-induced liver injury ultimately results in loss of liver function and hepatocyte death, other cell types, including LSECs, may also be directly exposed to toxic stress and contribute to overt tissue injury. Multiple lines of evidence from animal models have demonstrated LSEC injury as an early and critical event in paracetamol-induced hepatotoxicity, preceding hepatocyte death. In acetaminophen hepatotoxicity, formation of the reactive metabolite N-acetyl-p-benzoquinone imine and depletion of hepatic glutathione initiate cellular injury. Sinusoidal microcirculatory dysfunction and LSEC damage can occur early and may precede overt hepatocyte necrosis, while platelet accumulation and endothelial disruption further compromise sinusoidal perfusion [96]. Furthermore, the buildup of free cholesterol in LSEC endolysosomes exacerbates experimental paracetamol hepatotoxicity. Experimental studies identify sinusoidal microvascular dysfunction as a modifiable component of acetaminophen injury; matrix metalloproteinase (MMP) inhibition and NO-directed approaches can reduce endothelial injury, whereas evidence for α1-adrenoceptor antagonists and heparin is model-specific [97].

Aside from LSEC toxicity induced by dacarbazine, cyclophosphamide, or azathioprine, animal studies have further confirmed that pyrrolizidine alkaloids, lipopolysaccharide, and galactosamine also exert detrimental effects on LSECs [98, 99]. While the mechanisms of toxicity for paracetamol have been more thoroughly studied in mouse models, LSEC behavior in response to these toxicants is comparable. Shortly following paracetamol exposure, LSEC swelling occurs and compromises endogenous scavenging capacity. Severe endothelial disruption, including enlargement and coalescence of fenestrae into larger gaps, compromises sinusoidal integrity and reduces blood flow, resembling early changes induced by pyrrolizidine alkaloids in veno-occlusive disease [100]. When coupled with excessive ethanol, these paracetamol-induced processes are worsened. Though the earliest events of acute liver injury entail sinusoidal neutrophil accumulation and priming, and though neutrophil extravasation is believed to aggravate tissue damage, murine liver injury models that have compared combined galactosamine–endotoxin treatment with single endotoxin exposure have demonstrated that LSEC pore formation is independent of neutrophil priming and subsequent leukocyte migration [101]. Conversely, the formation of large LSEC pores facilitates neutrophil extravasation and enhances interactions between injured hepatocytes and sinusoid-accumulated neutrophils [102].

These toxicants may damage the LSEC cytoskeleton, whereas NO donors and MMP2/MMP9 inhibitors can attenuate endothelial injury in experimental models. Several of these toxicants als*o* reduce glutathione levels in LSECs in vitro. Inhibition of eNOS exacerbates LSEC injury, whereas suppression of inducible NO synthase attenuates such damage, supporting this hypothesis. Additionally, LSEC damage in animal models has been linked to oxidative stress that releases free radicals such as superoxide [6].

### LSECs in chronic liver diseases

#### LSECs in MASLD

**Fig. 6:**
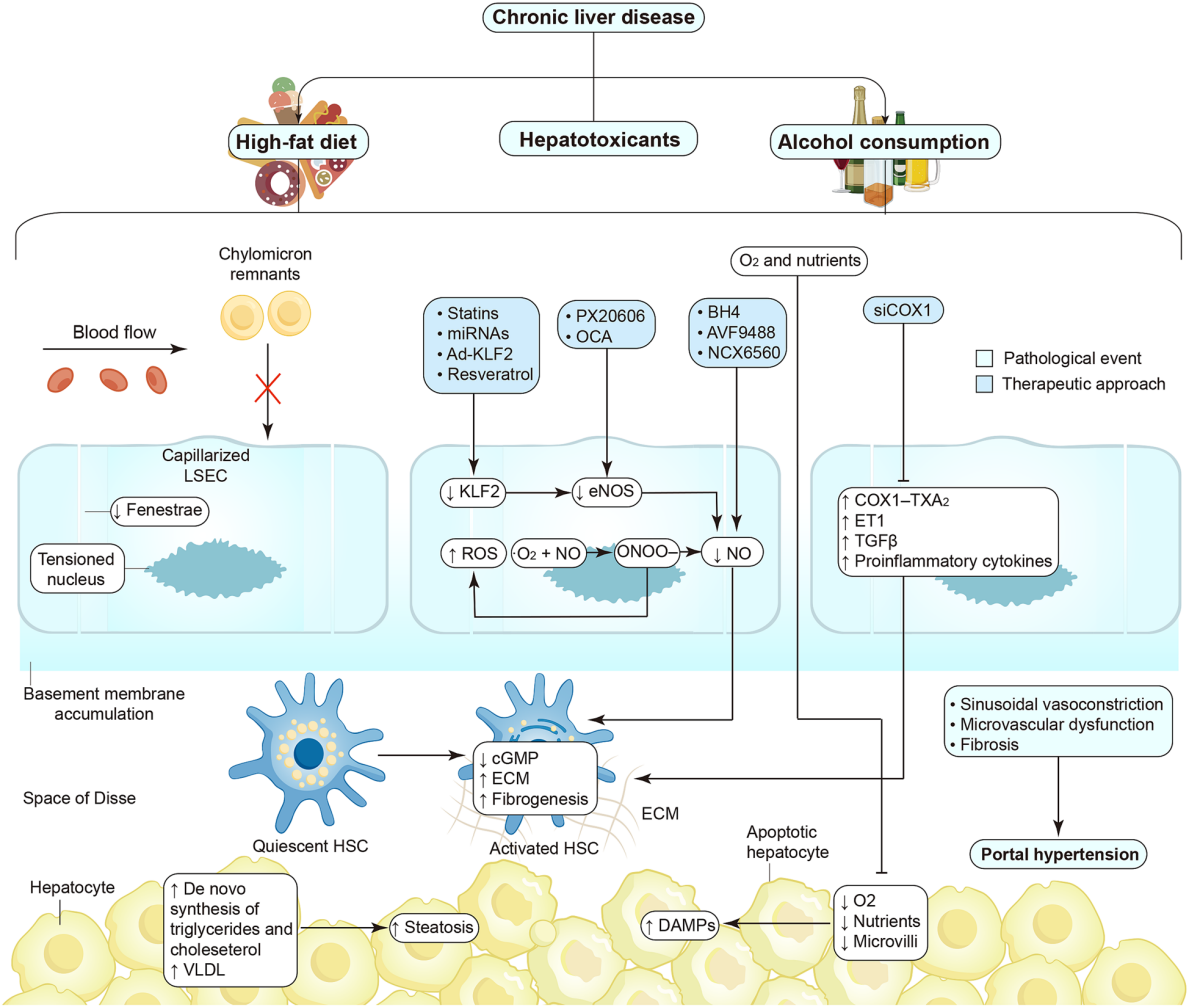
LSEC-mediated mechanisms in chronic liver injury. LSECs undergo marked dedifferentiation as a result of chronic liver injury, losing their vasoprotective properties and becoming prothrombotic, proinflammatory, and vasoconstrictive. These consequences include the formation of a basement membrane and loss of fenestrae, which impairs the exchange of oxygen, nutrients, and lipoproteins between sinusoidal blood and hepatocytes, thereby promoting steatosis and parenchymal injury. Reduced expression of KLF2 and eNOS activity decreases NO bioavailability and enhances ROS-mediated NO scavenging, thereby promoting ECM accumulation and HSC activation. Sinusoidal vasoconstriction is exacerbated by increased levels of pro-inflammatory cytokines and vasoconstrictors (including ET-1 or TXA2). Together, these changes increase intrahepatic vascular resistance and ultimately elevate portal pressure. Antioxidants, prostanoid synthesis inhibitors, and stimulants of the transcription factor KLF2 are examples of therapeutic approaches targeting these pathways. Reproduced with permission from Ref. [35]. Copyright © 2021, Springer Nature Limited

MASLD is characterized by hepatic steatosis together with at least one cardiometabolic risk factor. MASH represents the inflammatory form of the disease and is characterized by steatosis, hepatocellular injury, and lobular inflammation, with variable degrees of fibrosis [103, 104]. Even during the early stages of MASLD, before substantial inflammation or HSC activation, LSECs can undergo structural dedifferentiation characterized by capillarization and defenestration, accompanied by decreased eNOS activity and altered capillarization marker profiles [21, 28]. The loss of fenestrae and deposition of basement membrane transform LSECs from a highly permeable endothelium into a continuous, capillarized phenotype. These structural alterations precipitate cascading functional consequences. Capillarized LSECs exhibit markedly impaired vasodilatory capacity because of reduced NO bioavailability, resulting in elevated hepatic vascular resistance, portal hypertension, and hemodynamic dysfunction [105, 106]. Defenestration impairs sinusoidal permeability, hindering hepatic uptake of chylomicrons and retinol while obstructing lipid outflow. Together with decreased fatty acid oxidation, this promotes de novo lipogenesis and accelerates steatosis. Dysfunctional LSECs lose their capacity to maintain HSC quiescence and instead secrete profibrogenic mediators such as TGF-β, which, in concert with diminished NO signaling, induces HSC activation, ECM accumulation, and sinusoidal vasoconstriction (Fig. 6) [31]. Emerging preclinical evidence suggests that LSECs exhibit relatively elevated glutaminase 1 (GLS1) compared with hepatocytes, and that targeting GLS1 may alleviate MASLD by restoring VLDL export and assembly. However, whether LSEC-derived GLS1 directly drives disease progression in *vivo* requires validation with LSEC-specific models, and its therapeutic potential remains to be fully established.

Under physiological conditions, LSECs maintain a highly specialized phenotype characterized by fenestrations—transcellular pores organized in sieve plates—that facilitate the bidirectional exchange of lipoproteins and free fatty acids between the sinusoidal blood and hepatocytes. This process is essential for efficient hepatic lipid metabolism and prevents ectopic lipid accumulation. In MASLD, persistent exposure to excess FFAs, insulin resistance, and systemic inflammation leads to LSEC defenestration and capillarization. This is a structural and functional transformation marked by loss of fenestrae, deposition of subendothelial basement membrane, and increased expression of adhesion molecules. Capillarization rapidly compromises sinusoidal permeability, hindering hepatic uptake of chylomicrons and retinol while obstructing lipid outflow. Together with decreased fatty acid oxidation, this promotes de novo lipogenesis and accelerates steatosis [107]. In parallel, fenestration loss permits the accumulation of modified low-density lipoproteins, including oxidized LDL (oxLDL) and non-esterified free fatty acids in the space of Disse, where oxidized phospholipids and oxLDL directly activate HSCs [108]. Meanwhile, crosstalk with Kupffer cells amplifies lipotoxic signaling to promote macrophage polarization [31]. Importantly, these early capillarization events exhibit a striking predilection for the pericentral zone. Spatial omics analyses have confirmed that MASLD-associated molecular stress is not evenly distributed but concentrates in pericentral regions, where LSECs coexist with lipogenic hepatocytes and lipid-handling macrophages within a low-oxygen niche. Thus, pericentral LSECs endure a “triple hit” of lipotoxicity, oxidative stress, and hypoxia, which surpasses the buffering capacity of their intrinsic scavenging and antioxidant systems. This vulnerability explains why Zone 2/3 LSECs undergo early defenestration, despite the preserved structural integrity of periportal LSECs. Beyond these structural and microenvironmental changes, LSEC-intrinsic metabolic reprogramming further exacerbates disease progression. Preclinical evidence links hepatic GLS1 activity to VLDL assembly and lipid dysregulation [109, 110]. However, direct therapeutic evidence derives mainly from the experimental NASH model, whereas LSEC-specific causality remains unproven.

Recent spatially resolved multi-omics atlases of human MASLD have provided critical spatial context to these single-cell observations, revealing that LSEC dysfunction is not uniformly distributed but concentrated in specific lobular niches [111]. Unbiased deconvolution of spatial transcriptomics has delineated a fibrosis-associated gene program enriched in advanced MASH, demonstrating profibrotic crosstalk between central-vein endothelial cells and HSCs within fibrotic regions. Spatial metabolomics further identifies pericentral phospholipid buildup unique to MASLD, which likely arises from lipoprotein-associated phospholipase A2-driven metabolism within lipid-laden macrophages. These spatial data establish that LSEC capillarization in MASLD is a zonation-disrupting event: the loss of fenestration in pericentral LSECs physically obstructs lipid exchange, while the same cells undergo metabolic reprogramming that epigenetically amplifies pro-fibrogenic angiocrine signaling. Integrating single-cell and spatial omics thus reveals LSECs as both early sensors and spatial orchestrators of the MASLD pathological cascade [112]. Therefore, future therapeutic paradigms for MASLD should prioritize LSEC reprogramming over simple metabolic correction, aiming to restore fenestration, scavenger capacity, and KLF2-driven NO bioavailability as a primary disease-modifying strategy. Re-establishing the endothelial metabolic gatekeeper function through phenotypic reprogramming may halt disease progression prior to the occurrence of irreversible hepatocyte injury.

In conclusion, LSEC capillarization is increasingly recognized as a potential early pathogenic event and pathological amplifier in MASLD, rather than merely a secondary consequence of advanced disease. By physically blocking lipid exchange, generating oxidative stress, and directly activating HSCs through paracrine signaling, capillarized LSECs initiate and sustain the fibro-inflammatory cascade. Evidence that LSEC capillarization can precede overt steatosis and fibrosis supports further evaluation of endothelial phenotype normalization as an early disease-modifying strategy.

#### LSECs in MetALD

MetALD refers to individuals with elevated weekly alcohol intake and metabolic dysfunction-associated steatotic liver disease. This condition has been linked to dysregulation of LSEC differentiation and function [113]. LSEC capillarization and dysfunction are increased by impaired NO generation (Fig. 6). Thioredoxin-interacting protein (TXNIP), a key regulator of redox homeostasis, has been implicated in the regulation of endothelial NO production in LSECs. Experimental evidence suggests that increased TXNIP expression may preserve LSEC function and protect against MetALD progression, indicating that restoration of TXNIP signaling could represent a potential therapeutic strategy [114]. In addition, restoration of LSEC function requires control of alcohol-induced hepatic injury. For instance, MetALD therapy benefits from adeno-associated virus (AAV)-mediated gene transfer that blocks acetylation of Hsp90 selectively in LSECs [68]. Precise molecular mechanisms underlying LSEC dysfunction in MetALD remain incompletely defined.

Emerging evidence highlights that dysfunctional adipose tissue establishes a critical paracrine axis with LSECs in MetALD. In visceral obesity, hypertrophic adipocytes secrete elevated leptin, resistin, IL-6, and TNF-α levels, while adiponectin production is markedly reduced [115]. Leptin can activate JAK/STAT and MAPK signaling in hepatic and vascular cells; direct evidence that the complete LepRb–JAK2/STAT3–MAPK cascade drives ICAM1, VCAM1, E-selectin, and ET-1 expression specifically in LSECs remains limited [116]. In contrast, adiponectin exerts vasoprotective effects on LSECs via AdipoR1/R2-mediated activation of the AMPK-eNOS axis, which preserves fenestration and NO bioavailability. Therefore, hypoadiponectinemia in MetALD may remove a vasoprotective influence, although the cited evidence directly supports TXNIP-dependent NO regulation rather than an adiponectin-specific mechanism [114]. Furthermore, adipocyte-derived extracellular vesicles carry inflammatory miRNAs and proteins and are associated with metabolic stress; whether they are preferentially taken up by LSECs and directly trigger endoplasmic reticulum stress and NF-κB activation in *vivo* remains to be established [28, 117, 118]. These signals converge to repress LSEC-specific transcriptional programs (e.g., KLF2, GATA4) and promote the expression of capillarization markers (CD34, PECAM1, vWF) [10, 119]. Mechanistically, the suppression of KLF2 and GATA4 in MetALD impairs the eNOS-NO-sGC axis. KLF2 serves as a key shear-stress-responsive activator of eNOS transcription, while GATA4 preserves eNOS protein stability and coupling. Their downregulation therefore reduces NO bioavailability, diminishes cGMP-dependent inhibition of HSCs, and permits ET-1-driven sinusoidal vasoconstriction. Concurrently, adiponectin deficiency reduces AMPK-mediated eNOS phosphorylation and thereby lowers p-eNOS generation, and alcohol-induced Hsp90 acetylation further destabilizes total eNOS. Together, impaired KLF2/eNOS regulation, TXNIP-dependent NO signaling, and alcohol-induced Hsp90 acetylation provide a plausible multi-hit model of LSEC dysfunction, but the relative contribution of each component remains unresolved [54, 114, 120]. Thus, LSEC–adipocyte crosstalk may contribute to MetALD by disrupting the KLF2-eNOS-NO rheostat and establishes a self-sustaining loop of sinusoidal remodeling and HSC activation. Through restoration of NO bioavailability, reversal of leptin-driven adhesion molecule expression, or targeted epigenetic editing, therapeutic reprogramming of LSECs in MetALD may re-establish endothelial tolerance and reverse the lipotoxic-inflammatory axis. Such reprogramming approaches shift the therapeutic goal from damage control to active vascular normalization. Although early endothelial alterations may be reversible, recurrent or persistent injury can lock LSECs into a capillarized state, thereby establishing a pathological basis for chronic liver disease.

#### LSECs in chronic hepatotoxic injury

Experimental models of chronic liver injury demonstrate that LSEC capillarization and loss of endothelial homeostatic signaling accompany and can promote fibrogenesis [121]. Nevertheless, the chronic alcohol-mediated regulatory effects on LSECs remain poorly characterized. Accordingly, one study used transmission electron microscopy to evaluate LSEC fenestrae in an experimental model of long-term alcohol consumption and revealed a time-dependent reduction in sinusoidal porosity as a result of ethanol intake [122]. To understand the role of LSEC pathobiology in toxicant-induced liver damage, additional studies have employed experimental models, such as carbon tetrachloride and thioacetamide models, which mirror most of the hepatic and extrahepatic consequences associated with chronic liver disease. In these models, early LSEC defenestration is followed by progressive mechanical remodeling as fibrosis develops [123, 124]. This temporal sequence—LSEC defenestration preceding overt parenchymal damage—supports the interpretation of sinusoidal endothelial dysfunction as an early insult rather than solely a secondary consequence, challenging the traditional hepatocentric assumption. Recognizing LSECs as the “first responder” to chronic insult reframes therapeutic priorities toward early endothelial reprogramming.

Capillarized LSECs release soluble mediators that affect neighboring cells, promote pathological remodeling, and contribute to chronic liver disease complications such as portal hypertension [125]. Experimental studies have shown that LSECs produce more vasoconstrictors during cirrhosis. In rat models of chronic liver injury, ET-1 and the cyclooxygenase-1 (COX-1)–TXA_2_ pathway are activated, which leads to sinusoidal contraction and exacerbates microvascular dysfunction [126]. COX-1 inhibition corrected endothelial dysfunction in cirrhotic rat livers, supporting a role for the COX-1–TXA2 axis without excluding a contribution of COX-2 in other hepatic cell populations [127]. However, considering its action in other non-parenchymal cells, this isoform may in fact contribute to fibrogenesis. Leukotrienes and other eicosanoids generated from arachidonic acid are also produced by LSECs and contribute to portal hypertension and hepatic microcirculatory dysfunction in cirrhosis [128].

In chronic hepatotoxic injury, the pathogenesis of portal hypertension and fibrosis can be traced to the convergent failure of two interlocking endothelial axes. Oxidative stress and inflammatory cytokines suppress the mechanosensitive transcription factor KLF2, which transcriptionally drives eNOS expression. The RAP1A-RAF1 brake on Notch signaling is lost, permitting DLL4-mediated Notch activation that directly inhibits eNOS-sGC-cGMP signaling [43]. The resulting collapse in NO bioavailability causes dual consequences. On the one hand, it removes the cGMP-dependent paracrine restraint on HSCs, allowing TGF-β and ET-1 to activate Smad2/3 and RhoA/ROCK pathways and drive collagen deposition [37]. On the other hand, the sinusoidal vascular tone is also shifted toward vasoconstriction due to the loss of the counterbalancing influence of NO on the COX1-TXA2 and ET-1 pathways [129]. Consequently, capillarization, HSC activation, and portal hypertension reflect interacting changes in KLF2, Notch, and eNOS signaling; β-arrestin2-dependent GPCR–eNOS coupling is one defined component of this broader endothelial network [130]. Vasodilators and vasoconstrictors are out of balance in the liver sinusoids, This imbalance is exacerbated by crosstalk between NO signaling and the endothelial COX-1–TXA_2_ pathway in cirrhosis.

### LSECs in liver infections

**Fig. 7:**
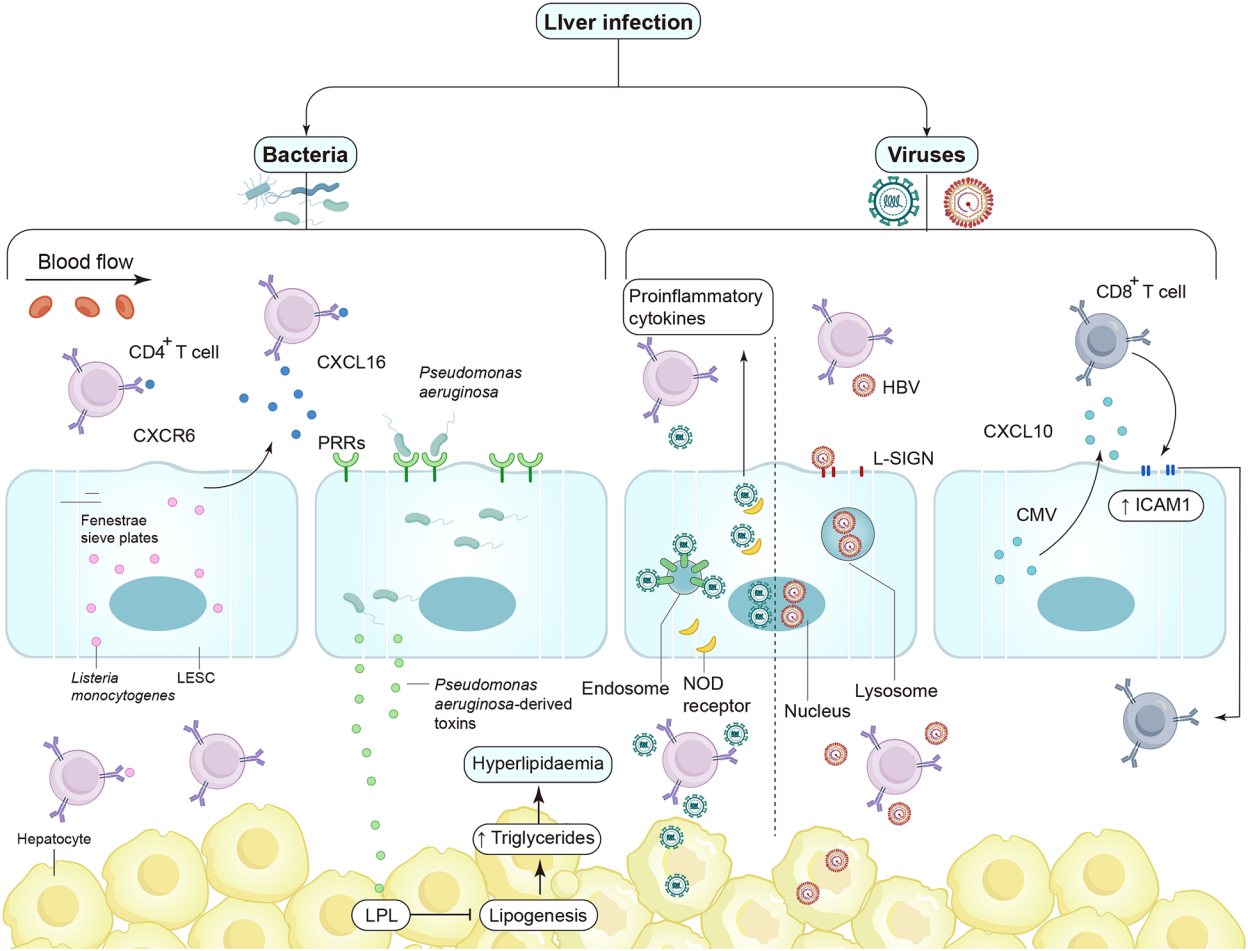
LSEC-mediated mechanisms in liver infection. *Listeria monocytogenes* infection causes LSECs to release CXCL16, which attracts cytotoxic CD8^+^ T cells that express its receptor CXCR6. Bacterial pathogen-associated molecular patterns and virulence factors activate PRR signaling and facilitate *Pseudomonas aeruginosa*’s infection of LSECs, resulting in lipoprotein retention in parenchymal cells and subsequent hyperlipidemia linked to bacterial sepsis. During viral infection, LSECs may sense HBV and HCV through distinct PRRs, contribute to viral transcytosis or trans-infection, and release proinflammatory mediators that recruit cytotoxic CD8^+^ T cells. ICAM1 expression and CXCL10 production are elevated in CMV infection to recruit T helper CD4^+^ cells to the tissue microenvironment. Reproduced with permission from Ref. [35]. Copyright © 2021, Springer Nature Limited

The chronically activated and capillarized sinusoid created by metabolic injury not only permits steatosis and fibrosis but also fundamentally alters hepatic immune surveillance. In contrast to the lipotoxic and mechanically driven capillarization observed in MASLD and cirrhosis, LSEC dysfunction in viral infections is primarily immunologically driven. In this setting, the shared feature of LSEC activation (such as the upregulation of adhesion molecules) diverges into a highly disease-specific mechanism, namely the active exploitation of endothelial immune checkpoints. Within this altered milieu, failure of host immunity to terminate acute infection can lead to chronic viral infection [131]. LSEC activation can alter antigen presentation, leukocyte trafficking, and local tolerance, but a direct endothelial checkpoint mechanism of viral persistence remains incompletely established [132]. Hepatitis A and most hepatitis E infections are acute and self-limited, whereas HBV, HCV, and HDV can establish chronic infection in susceptible hosts [133–135] (Fig. 7).

#### LSECs in HBV

Chronic HBV infection is a major global cause of cirrhosis and HCC [136, 137]. HBV clearance requires an early innate immune response followed by the adaptive immune response. Murine studies show that HBV can elicit host innate immunity via TLR cascades within hepatic non-parenchymal cells such as LSECs to suppress viral replication [138–140]. Accordingly, preclinical research on HBV infection has demonstrated that interventions stimulating immune cells and liver parenchymal and non-parenchymal cells with proinflammatory molecules such as interferon, TNF, and toll-like receptor (TLR) ligands result in the production of antiviral mediators like type I interferon, which in turn prevent viral replication [138]. The utilization of semaphorin 4D as a CD8^+^ T cell response promoter for HBV clearance in LSECs has been evaluated in a preclinical study, and the results suggest that semaphorin 4D can activate LSECs as APCs [141].

#### LSECs in HCV

Human liver endothelial cells respond to HCV with autocrine interferon signaling and release antiviral exosomes; this supports an antiviral endothelial role but does not by itself demonstrate direct recruitment of CD8^+^ T cells or eradication of infected hepatocytes [142]. Primary human LSECs and hepatocytes provide useful experimental systems for studying chronic liver disorders such as HCV infection. Paracrine crosstalk between these two cell types drives elevated BMP4 expression, suppressed VEGFR-2 activity and endothelial hyperproliferation, collectively facilitating HCV propagation [143]. Indeed, preclinical research has indicated that LSECs may restrict HCV propagation upon administration of immune response regulators like interferon and TNF [144].

While the role of LSECs in chronic viral hepatitis is still not fully understood, research on LSEC dysregulation following acute viral infection may help clarify how some infections progress to cirrhosis and inform targeted therapies. Understanding the potential impacts of direct-acting antiviral drugs on the LSEC phenotype also warrants investigation. Accordingly, clinical research has indicated that direct-acting antiviral therapy improves endothelial function in HCV patients, as evidenced by decreased expression of endothelial cell adhesion molecules such as ICAM1 and E-selectin [145, 146].

### LSECs in HCC

**Fig. 8:**
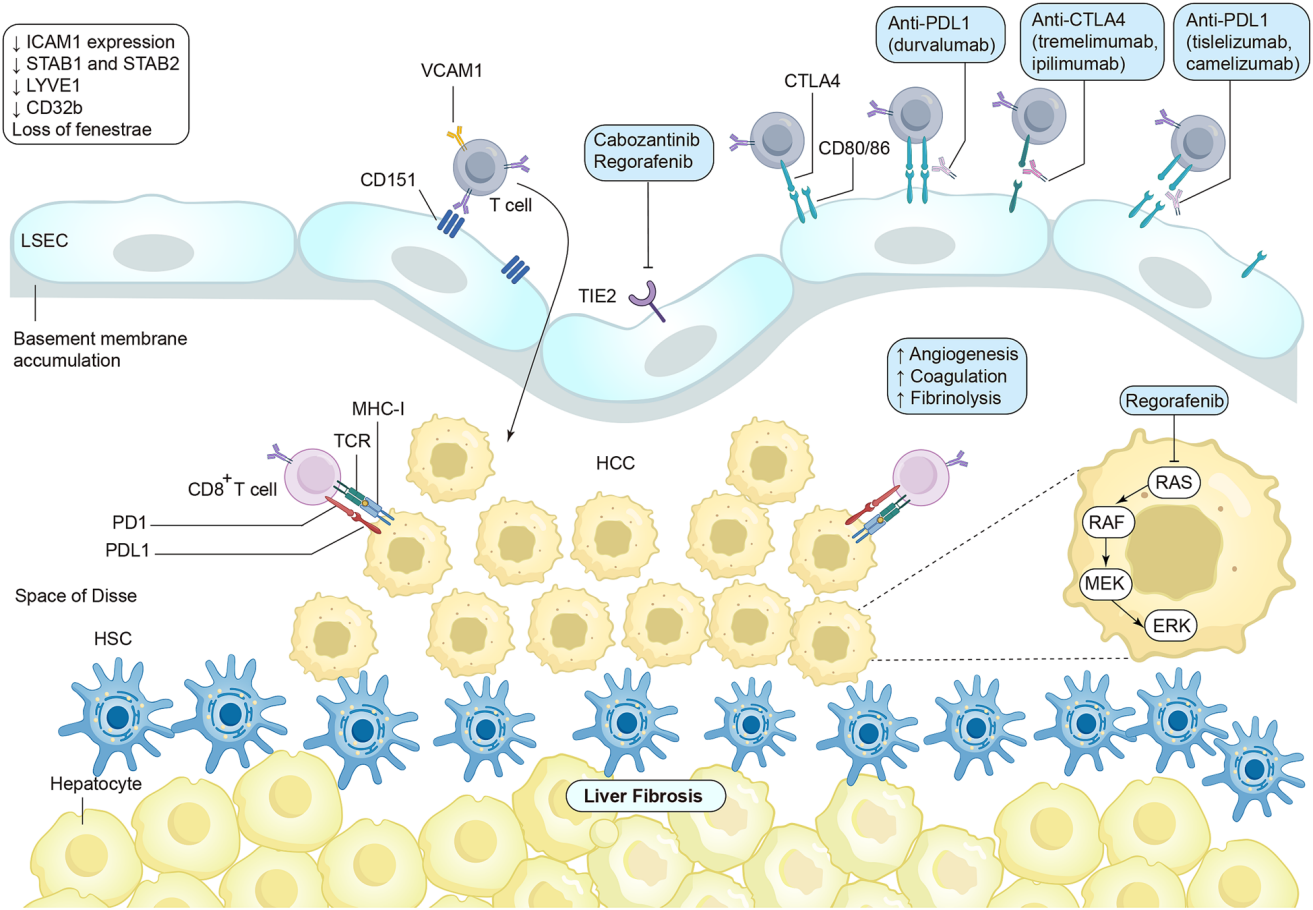
LSEC-mediated mechanisms in hepatocellular carcinoma. In the HCC tumor microenvironment, LSECs are capillarized and take part in angiogenesis, coagulation, and fibrinolysis. Tumor-associated LSECs downregulate ICAM1, STAB1, STAB2, LYVE1, and FcγRIIb/CD32b, reflecting a shift away from the canonical LSEC phenotype. They lose fenestrae and express more CD151, which promotes the recruitment of T cells through VCAM1. LSECs in HCC may also express CD80 and CD86, which interact with CD28 and CTLA4 on T cells and contribute to local immune regulation. The interaction of PD1 on antitumor CD8^+^ T cells with PD-L1 in the tumor microenvironment can suppress T-cell function. Various immune-checkpoint antibodies that broadly act within the HCC tumor microenvironment are being studied, including durvalumab for PD-L1, tremelimumab and ipilimumab for CTLA4, and tislelizumab and camrelizumab for PD1. Regorafenib and cabozantinib broadly inhibit angiogenic kinase signaling but do not selectively target TIE2 or restore canonical LSEC identity. Reproduced with permission from Ref. [35]. Copyright © 2021, Springer Nature Limited

Decades of persistent infection and chronic inflammation lock the LSEC in a pathologically activated state, creating a microenvironment in which immune surveillance is suppressed, HSCs remain activated, and angiogenic signaling is aberrant. Within this compromised sinusoidal niche, tumor-associated endothelial states can reinforce HCC immune resistance through CXCL12-dependent suppression of cytotoxic T-cell differentiation and recruitment of myeloid-derived suppressor cells [147]. Persistent inflammatory liver injury induces genetic and epigenetic aberrations, which transform premalignant lesions endowed with proliferative, invasive, and anti-apoptotic phenotypes into HCC [148] (Fig. 8). Divergent from the zonal metabolic vulnerability observed in MASH or the scar-associated reprogramming observed in cirrhosis, LSEC vulnerability in HCC is primarily imposed by tumor-derived hypoxia, sustained VEGF signaling, TGF-β-rich stromal cues, and immune checkpoint pressure, which jointly convert sinusoidal endothelial cells into tumor-conditioned endothelial subsets.

In the HCC microenvironment, endothelial remodeling and immune suppression are interconnected [21]. Tumor endothelial cells can promote IL-4-dependent M2 macrophage polarization and thereby support tumor growth [149]. At the molecular level, this coupling is driven by convergent hypoxia-, VEGF-, TGF-β-, and Notch-dependent signaling. Hypoxia/HIF-1α simultaneously promotes VEGF-mediated pathological vascular remodeling and programmed cell death 1 ligand 1 (PD-L1)-associated T-cell exhaustion, while TGF-β and Notch signals strengthen endothelial dedifferentiation, capillarization, and immune-excluding angiocrine programs. This integrated mechanism exhibits preferential vulnerability in pericentral LSECs, rooted in pre-existing zonal stress. Chronic metabolic insults that initiate in Zone 2/3—such as lipotoxicity and hypoxia—induce a senescent, dysfunctional endothelial state long before the development of gross neoplasia. In addition, recent spatial transcriptomic studies have demonstrated that these stresses establish multicellular hubs in pericentral regions where stressed LSECs, senescent hepatocytes, and activated HSCs create a tumor-permissive niche. As HCC develops within this compromised niche, LSECs lose their fenestrae, deposit a basement membrane, and downregulate scavenger receptors (STAB1, STAB2, LYVE1, CD32b) and adhesion molecules (ICAM1) [24]. Finally, a pro-angiogenic phenotype driven by VEGF signaling and fibrinolytic activity is acquired. Crucially, these structural and functional alterations simultaneously serve both vascular remodeling and immune evasion. The newly formed basement membrane and disorganized, leaky sinusoids create a physical barrier that impedes CD8^+^ T cell infiltration, while persistent hypoxia stabilizes HIF-1α to transcriptionally upregulate VEGF and PD-L1. TGF-β1 from the fibrotic stroma promotes capillarization and favors the recruitment of regulatory T cells (Treg cells) and myeloid-derived suppressor cells (MDSCs) [7]. Consequently, the zonal identity of LSECs dictates both the anatomical origin of HCC and the inherent predisposition of region-specific sinusoidal endothelium toward facilitating tumor progression: LSEC transdifferentiation simultaneously remodels the tumor vasculature and establishes an immunosuppressive niche through a shared set of HIF-1α- and TGF-β1-dependent programs.

LSECs have antigen-presenting functions and can express CD80/CD86 and PD-L1. CD80/CD86 engage CD28/cytotoxic T-lymphocyte antigen 4 (CTLA4) on T cells, whereas PD-L1 engages programmed cell death protein 1 (PD1) [150]. In the tumor microenvironment, changes in these ligand–receptor interactions can shift LSEC–T-cell communication from activation toward immune suppression [151].

At the molecular level, the immunomodulatory role of LSECs in HCC extends beyond checkpoint expression to active orchestration of the tumor immune microenvironment [2]. LSECs internalize tumor-associated antigens via scavenger receptors (e.g., STAB1/2, L-SIGN, SR-B1) and cross-present them to CD8^+^ T cells through major histocompatibility complex (MHC) class I, a process essential for initiating antitumor immunity. Recent functional evidence identifies immunosuppressive tumor-associated endothelial subsets in HCC, including CXCL12^+^ cells that impede CD8^+^ T-cell differentiation and recruit myeloid-derived suppressor cells [147]. This phenotypic switch disrupts the immunological synapse and drives CD8^+^ T-cell exhaustion. In parallel, LSECs secrete CCL22 and CXCL12, which recruit Treg cells and MDSCs into the tumor bed, further consolidating immune privilege. Interactions between LSECs and tumor-associated macrophages further potentiate this immunosuppressive microenvironment: LSEC-derived angiopoietin-2 (ANGPT2) engages TIE2 on macrophages to promote M2 polarization, while TAM-derived IL-10 and prostaglandin E2 stabilize PD-L1 expression on LSECs via STAT3 signaling. Evidence that LSECs directly regulate natural killer (NK)-cell cytotoxicity through MICA/B and ULBP ligand display in HCC remains insufficient; this mechanism should be treated as a hypothesis rather than an established pathway [147]. Collectively, these molecular networks position LSECs as central regulators of immune evasion in HCC [2]. HCC-associated PD-L1 overexpression in LSECs, paralleling expression patterns in malignant cells, dampens T-cell functionality and antitumor potency. Meanwhile, LSEC-derived CD151 upregulation modulates VCAM1 to facilitate intratumoral T-cell recruitment, creating conflicting immunomodulatory effects [152]. Beyond its role in lymphocyte trafficking, CD151 functions as a disease-classification biomarker and a therapeutic vulnerability in HCC. CD151 expression on tumor-associated LSECs is associated with advanced tumor stage, portal vein invasion, and poor overall survival, distinguishing aggressive HCC subtypes from indolent disease. Mechanistically, by delivering integrin-β1-enriched cargo to neighboring endothelial and cancer cells, CD151-enriched migrasomes derived from LSECs and tumor cells promote angiogenesis and condition the metastatic niche, thereby sustaining vascular mimicry and immune exclusion. Therapeutically, CD151-targeted siRNA delivered via exosomes or nanoparticles has been shown to restore endothelial homeostasis, reduce VCAM1-mediated immunosuppressive cell recruitment, and enhance the efficacy of checkpoint inhibitors in preclinical models. This further underscores CD151 as a rational cotarget alongside PD-L1/PD1 blockade [152, 153].

Notably, the immunosuppressive and pro-angiogenic functions of LSECs are mechanistically coupled rather than operating in isolation. Identical tumor microenvironmental cues that drive LSEC capillarization and aberrant angiogenesis, such as chronic VEGF exposure, TGF-β1 signaling, and hypoxia, simultaneously induce the expression of immune checkpoints (PD-L1, galectin-9, VISTA) and the secretion of immunosuppressive chemokines (CCL22, CXCL12) [154]. Hypoxia resulting from disorganized tumor vasculature further amplifies both programs by stabilizing HIF-1α, which transcriptionally upregulates VEGF and PD-L1 in endothelial cells, thereby establishing a self-reinforcing vascular-immune axis [155]. In this unified disease model, abnormal angiogenesis and immune evasion are not sequential or separate pathological events but are co-dependent processes rooted in LSEC dysfunction, jointly fostering a tumor-permissive microenvironment.

Integrative single-cell and spatial transcriptomic analyses of human HCC specimens have recently identified a distinct tumor endothelial cell subpopulation termed S100A16^⁺^ TECs, which is significantly enriched in human HCC tissues and coordinately drives both pathological angiogenesis and immunosuppression. Mechanistically, S100A16 overexpression in murine endothelial cells promotes proliferation, migration, and tube formation, while simultaneously secreting CCL20 and TGF-β2 to induce Treg differentiation. As confirmed by spatial transcriptomics in human samples, S100A16^⁺^ TECs co-localize with Treg infiltration and dysfunctional vasculature. Endothelial-specific S100A16 knockout in murine HCC models significantly reduces tumor angiogenesis, Treg infiltration, and HCC growth in *vivo.* These findings highlight how single-cell and spatial transcriptomic technologies extend the traditional concept of hepatic capillarization. By identifying disease-promoting endothelial subsets, including S100A16⁺ tumor endothelial cells, these approaches elucidate the linkage between vascular remodeling and immune suppression in liver malignancy. They have also revealed the role of specific LSEC subpopulations as molecular hubs that simultaneously remodel the tumor vasculature and establish an immunosuppressive niche [156]. Dysfunctional LSECs also secrete profibrotic mediators, including ET-1, PDGF, and TGF-β, that further drive HSC activation and ECM deposition. In turn, activated HSC-derived TGF-β and matrix stiffness further suppress LSEC identity and reinforce capillarization, forming a feed-forward loop between endothelial dedifferentiation and fibrogenesis. Consequently, therapeutic reprogramming in HCC should target not merely VEGF signaling but also the specific angiocrine-immune programs (e.g., S100A16-CCL20-TGF-β2 axis) executed by pathogenic endothelial subsets.

LSECs play a role in attracting additional tissue-derived tumorigenic cells to the liver. As a docking signal for adhesion molecule-driven infiltration of tumorigenic cells, β2 integrin expression promotes the metastatic progression of various solid malignancies, including colorectal cancer, to the liver [157]. Thus, in LSECs, ICAM1 inhibition decreases tumor-cell adhesion and transmigration both in *vivo* and in *vitro* [158]. Furthermore, melittin nanoparticle-induced LSEC immunological regulation promotes antitumor immune responses in a mouse model of spontaneous liver metastases [159].

The pro-fibrogenic microenvironment created by activated HSCs directly contributes to LSEC capillarization, forming a pathological feedback loop that exacerbates sinusoidal dysfunction and HCC progression. Activated HSCs are indeed a major source of VEGF. While VEGF supports physiological endothelial maintenance, HCC contains heterogeneous tumor endothelial states with distinct angiogenic and immune programs rather than a single uniform response to VEGF [160]. Importantly, this HSC-LSEC axis generates a dual-hit mechanism. Specifically, by promoting endothelial PD-L1 expression, expanding Treg cells and MDSCs, and polarizing tumor-associated macrophages toward an M2 phenotype through ANGPT2-TIE2 signaling, HSC-derived VEGF not only induces LSEC capillarization and pathological angiogenesis but also actively remodels the immune microenvironment. Thus, the vascular remodeling driven by activated HSCs and the immunosuppression mediated by tumor-conditioned LSECs represent two sides of the same pathological coin. They jointly establish a tumor-permissive microenvironment that couples abnormal vessel growth with effective immune evasion.

This dual vascular-immune effect is reinforced by other HSC-derived mediators, including PDGF, TGF-β, and CTGF. They may cooperate with VEGF to sustain abnormal tumor endothelial states, although their relative contribution varies among endothelial subsets [160]. Mechanistically, the transition from physiological to pathological VEGF signaling in HCC reflects a dose-dependent switch in downstream VEGFR2 signaling. Under homeostatic conditions, VEGF transiently activates Akt-eNOS to generate NO, which preserves fenestration and maintains Wnt2/HGF angiocrine secretion that supports hepatocyte regeneration. In the tumor microenvironment, persistent high-level VEGF, together with TGF-β and hypoxia, redirects VEGFR2 signaling from Akt-eNOS toward sustained ERK1/2 activation and eNOS uncoupling. Such endothelial dysfunction can reduce NO bioavailability and shift signaling toward vasoconstrictive and profibrogenic outputs; direct suppression of LSEC-derived Wnt2 and HGF in HCC requires further evidence [161]. Meanwhile, TGF-β secreted by activated HSCs and dysfunctional LSECs transcriptionally represses LSEC-enriched transcription factors (KLF2, GATA4) and upregulates capillarization markers (CD34, PECAM1, vWF) and immune checkpoints (PD-L1) through Smad and non-Smad pathways. Thus, VEGF, TGF-β, and hypoxic Notch ligands do not operate as independent pathogenic inputs, but converge on LSECs to co-drive a unified program of capillarization, aberrant angiogenesis, and immune evasion. This bidirectional activation further underscores the importance of targeting the HSC-LSEC axis in anti-HCC therapeutic strategies. VEGF’s dual role in HCC—initially maintaining LSEC differentiation via VEGFR2 yet ultimately driving capillarization through dose-dependent ERK persistence—exemplifies the broader context-dependency of angiocrine signaling. Consequently, future therapeutic design should distinguish physiological endothelial support from pathological angiogenic signaling and prioritize vascular normalization where feasible [161]. Beyond conventional checkpoint inhibition or anti-angiogenic blockade, reprogramming tumor-associated LSECs toward a quiescent, immunostimulatory phenotype—restoring antigen presentation, reducing PD-L1, and normalizing angiocrine secretion—offers a transformative strategy to dismantle immune privilege. Re-establishing normal LSEC identity within the tumor microenvironment can normalize vasculature, enhance drug delivery, and reinvigorate antitumor immunity.

### LSECs and therapeutic resistance in HCC

One emerging mechanism involves 14–3-3η-associated signaling in HCC cells and tumor-associated macrophages; whether an analogous feedback loop operates in LSECs remains uncertain. In tumor cells, overexpression of 14–3-3η, a member of the 14–3-3 family of scaffold proteins, stabilizes phosphorylated ERK1/2 (p-ERK1/2), preventing its dephosphorylation and nuclear export. This sustained ERK activation enhances the production of pro-angiogenic factors such as IL-8, fibroblast growth factor 2, and ANGPT2. These factors act in a paracrine manner on neighboring LSECs, promoting their proliferation, migration, and tube formation even under low-VEGF conditions [162]. Available studies support a tumor-cell and tumor-associated macrophage 14–3-3η/NF-κB circuit that promotes HCC drug resistance [163, 164]. Direct demonstration of an analogous 14–3-3η–ERK feedback loop within LSECs remains lacking.

These pathological fluctuations manifest structurally as disorganized, dilated, and leaky tumor-associated sinusoids with irregular pericyte coverage and heterogeneous blood flow. These features reflect neovascular dysfunction rather than normal sinusoidal homeostasis. Such vessels impair drug delivery, reduce oxygen perfusion (exacerbating hypoxia), and promote immune exclusion, all contributing to resistance against anti-angiogenic therapies (sorafenib and lenvatinib) [165, 166] and immunotherapies [167]. Functionally, by simultaneously starving the tumor of oxygen and drugs while excluding cytotoxic immune cells, this neovascular dysfunction constitutes a unified mechanism of therapeutic resistance. The resulting hypoxia reduces the efficacy of anti-angiogenic agents (sorafenib and lenvatinib). Meanwhile, it also drives HIF-dependent upregulation of PD-L1, promotes the recruitment of Treg cells and MDSCs, and polarizes macrophages toward an M2 phenotype, directly diminishing the efficacy of checkpoint inhibitors. Consequently, the LSEC-mediated vascular–immune axis may represent an important pathological node, and disruption of both vascular and immune components may help overcome therapeutic resistance in HCC. Moreover, advanced HCC contains activated endothelial subsets with adhesion and chemokine programs that may facilitate recruitment of immunosuppressive cells; the exact contribution differs among tumor endothelial states [160, 168]. This further insulates the tumor from immune surveillance and diminishes the efficacy of checkpoint inhibitors. Therefore, tumor-derived 14–3-3η signaling represents a potential upstream driver of tumor-endothelial crosstalk and therapy-resistant angiogenesis, but direct LSEC-autonomous evidence is lacking. Targeting these non-canonical circuits in tumor cells or macrophages, or disrupting downstream tumor-endothelial crosstalk, may offer promising strategies to normalize the tumor vasculature, improve drug delivery, and overcome treatment resistance.

Taken together, these disease settings illustrate different routes to a shared endothelial outcome: loss of the fenestrated, scavenger-rich, and tolerogenic LSEC phenotype, followed by acquisition of inflammatory, fibrogenic, angiogenic, or immunosuppressive functions. This comparison establishes the theoretical basis for the subsequent section, which assesses therapeutic strategies by their ability to preserve, restore, or bypass the diseased LSEC state, rather than merely inhibiting downstream fibrosis, inflammation, and angiogenesis.

## Therapeutic strategies affecting LSEC dysfunction and reprogramming

Given that LSEC capillarization recurs across multiple liver injuries, the sinusoidal endothelium represents an attractive but technically challenging therapeutic entry point. The diseased LSEC is not the same target as the quiescent LSEC: as capillarization progresses, fenestrae are lost, subendothelial matrix accumulates, and scavenger receptors such as STAB1, STAB2, CD32b, and LYVE1 may decline, reducing ligand-mediated uptake and receptor-based delivery. Therefore, LSEC-directed therapy should be evaluated according to disease stage, endothelial phenotype, and delivery accessibility.

A second consideration is cellular specificity. Improvement in LSEC phenotype does not necessarily mean that an intervention directly acts on LSECs. Many agents primarily act on hepatocytes, HSCs, macrophages, immune cells, tumor cells, or the broader vascular compartment, with secondary benefits on sinusoidal endothelium. Therefore, in this section, direct LSEC reprogramming is distinguished from indirect endothelial protection and non-specific vascular or tumor-directed effects.

### Therapeutic reprogramming strategies targeting dysfunctional LSECs

Capillarization alters both the biological identity and the physical accessibility of LSECs. Defenestration, basement membrane deposition, elevated CD34 expression, and reduced levels of LYVE1, STAB2 and other scavenger receptors collectively render diseased hepatic endothelium refractory to conventional LSEC-targeted drug delivery. Consequently, traditional ligands such as hyaluronan, modified albumin, or scavenger-receptor-targeted nanocarriers exhibit reduced binding affinity and sinusoidal penetration in advanced fibrosis and cirrhosis. This triggers a stage-dependent therapeutic dilemma. At early or partially reversible stages, remaining scavenger-receptor expression and incomplete barrier remodeling may still permit ligand-guided endothelial delivery. Once capillarization becomes advanced, however, the combination of receptor loss, matrix deposition, and reduced fenestral access renders receptor- or fenestrae-dependent platforms much less predictable. Therefore, therapeutic strategies should be adapted to the capillarization stage: in cases of retained endothelial plasticity, phenotypic re-normalization should be prioritized to restore fenestration, KLF2-eNOS-NO signaling, and scavenger receptor expression. Conversely, when conventional LSEC targeting becomes ineffective, capillarization-resistant delivery platforms that bypass receptor loss and physical sinusoidal barriers are required [3, 35].

Targeted delivery may become more effective after phenotypic normalization of the hepatic sinusoidal endothelium. Growing evidence indicates that sGC activators restore LSEC fenestrae and scavenger receptor expression, effectively “re-opening” the sinusoidal gate. Similarly, simvastatin-loaded polymeric nanoparticles reverse capillarization and normalize LSEC phenotype, which in turn permits subsequent nanoparticle assemblies to access hepatocytes or the endothelial surface with restored efficiency. Within this therapeutic framework, LSEC phenotypic restoration may serve as an enabling strategy rather than merely a supplementary intervention [5, 168].

Capillarization-resistant delivery platforms are a promising research direction. In cases of incomplete phenotypic reversal, engineered platforms can bypass the capillarized barrier. Sequential nano-penetrators employ ECM-degrading enzymes or size-switchable designs to cross the basement membrane and collagenous septa before releasing therapeutic payloads. More recently, spherical nucleic acids have been shown to transfect capillarized LSECs directly without relying on scavenger-receptor-mediated uptake, offering a receptor-independent delivery strategy. However, direct evidence demonstrating restoration of fenestration in capillarized LSECs by this approach remains to be established. These platforms decouple therapeutic efficacy from the preservation of canonical LSEC receptors, providing a potential alternative for advanced capillarization. Accordingly, proposed LSEC-directed strategies can be classified according to capillarization sensitivity. Scavenger receptor-, LYVE1-, or fenestrae-dependent delivery systems are likely most effective in early disease or after partial endothelial re-normalization. In contrast, provided that delivery platforms can access diseased LSECs, intracellular reprogramming targets such as KLF2, GATA4, eNOS coupling, Notch inhibition, mechanotransduction pathways, and epigenetic regulators may remain therapeutically relevant after capillarization. Ultimately, receptor-independent systems, including size-adaptive nanoparticles, ECM-penetrating carriers, and spherical nucleic acids, do not rely on preserved canonical scavenger-receptor uptake, rendering them more suitable for advanced capillarized LSECs [30, 43, 52, 59].

### Novel therapeutic strategies for acute liver diseases

Therapeutic strategies in acute liver injury can be grouped according to the dominant endothelial process targeted: oxidative stress and mitochondrial injury, vasoregulatory failure, inflammatory adhesion and leukocyte recruitment, and repair of the sinusoidal barrier. This classification helps distinguish agents that directly preserve LSEC viability from those that indirectly protect LSECs by reducing hepatocyte injury or inflammatory amplification.

#### Treatment for ischemia–reperfusion injury

Several therapeutic agents have been investigated for their ability to reduce LSEC damage caused by ischemia–reperfusion injury. Benefits of novel recombinant human manganese superoxide dismutase against cold preservation and warm reperfusion injury have been validated in primary rat and human LSEC cultures as well as transplanted liver graft specimens [169]. A study using a mouse model of warm ischemia–reperfusion injury demonstrated that SEW2871, a selective agonist of sphingosine 1-phosphate receptor 1, enhances vasorelaxation, maintains vascular integrity, and promotes LSEC survival [170]. As an antagonist of endothelin receptors, bosentan has been shown to minimize DNA damage and maintain mitochondrial viability in mouse LSECs [171]. In preclinical research in rats, inhibition of hepatic MMP9 has also been suggested as a potential therapy to improve ischemia–reperfusion damage by attracting sinusoidal progenitor cells in both steatotic and non-steatotic livers [74] (Table 1). These interventions primarily preserve LSEC viability, mitochondrial integrity, vasorelaxation, or inflammatory restraint, making them most likely to be effective before advanced capillarization has caused irreversible loss of fenestration and scavenger receptor expression.

**Table 1:** Summary of therapies targeting LSECs

Study population	Disease	Treatment	Mechanistic effects on LSECs	LSEC specificity	NCT number	Trial status	Refs
Acute liver injury	Ischemia–reperfusion injury	JWH133	Reduces LSEC oxidative stress and inflammatory stimulation; decreases adhesion-molecule expression through CB2 signaling	Direct LSEC protection	N/A	Preclinical	[83]
		Simvastatin	Activates KLF2; upregulates eNOS and increases NO bioavailability; attenuates endothelial dysfunction and liver injury	Direct endothelial protection	N/A	Preclinical	[172]
			Upregulates KLF2 expression; activates autophagy and improves LSEC viability	Direct endothelial protection	N/A	Preclinical	[75]
			Ameliorates microvascular dysfunction, preserves LSEC fenestrae, and reduces hepatic oxidative stress	Direct endothelial protection	N/A	Preclinical	[80]
		Recombinant MnSOD	Inhibits AP-1 activation, maintains NO levels, reduces oxidative stress in LSECs, and prevents endothelial dysfunction	Direct endothelial protection	N/A	Preclinical	[173]
		CGS21680	Protects the LSEC phenotype and attenuates oxidative stress in LSECs	Direct endothelial protection	N/A	Preclinical	[174]
		Atorvastatin	Promotes eNOS activation and suppresses inflammation by preventing NF-κB activation	Indirect endothelial protection	N/A	Preclinical	[175]
		SEW2871	Increases LSEC survival, improves vasorelaxation, and reduces intrahepatic inflammation through S1PR1	Direct endothelial protection	N/A	Preclinical	[170]
		Bosentan	Reducing oxidative stress and DNA damage in LSECs	Indirect endothelial protection	N/A	Preclinical	[176]
		MMP2/9 inhibitor	Preserves LSEC integrity, supports liver regeneration through progenitor-cell recruitment, and decreases profibrotic and proinflammatory cytokines	Indirect endothelial repair	N/A	Preclinical	[177]
		Telluric acid	Increases endothelial eNOS and restores LSEC fenestrae	Direct endothelial protection	N/A	Preclinical	[178]
		Apelin 13	Increases eNOS and restores LSEC fenestrae	Direct endothelial protection	N/A	Preclinical	[179]
		ALINO73	Reduces adhesion-molecule expression and restores LSEC fenestrae	Direct endothelial protection	N/A	Preclinical	[180]
	Paracetamol-induced liver injury	V-PYRRO/NO	Inhibiting toxic injury progression; restoring LSEC fenestrae	Direct LSEC reprogramming	N/A	Preclinical	[181]
		MMP2/MMP9 inhibitor	Ameliorates vascular injury, accelerates liver regeneration, and reduces red-blood-cell infiltration	Indirect endothelial repair	N/A	Preclinical	[182]
		Prazosin	Attenuating LSEC microcirculatory dysfunction	Indirect endothelial protection	N/A	Preclinical	[183]
		Lactoferrin	Ameliorates LSEC microcirculatory dysfunction through Kupffer-cell modulation	Direct endothelial protection	N/A	Preclinical	[184]
			Decreasing LSEC ferroptosis	Direct endothelial protection	N/A	Preclinical	[185]
	Sinusoidal obstruction syndrome	Defibrotide	Prevents SOS development and protects LSECs	Indirect endothelial protection	NCT02851407	Completed	[186]
		V-PYRRO/NO	Restoring NO levels; preserving LSEC integrity	Direct LSEC reprogramming	N/A	Preclinical	[187]
		Recombinant thrombomodulin	Improves the LSEC phenotype and increases eNOS expression	Direct endothelial protection	N/A	Preclinical	[188]
		U46619	Attenuates monocrotaline-induced endothelial injury and preserves LSEC integrity through TXA2-receptor signaling	Unknown	N/A	Preclinical	[189]
	Acute bacterial and viral infections	poly(I:C)	Modulates interferon, cytokine, and chemokine responses; increases the leukocyte fraction; and inhibits HBV replication	Unknown	N/A	Preclinical	[190, 191]
		P3C	Activates CD8^⁺^ T cells	Unknown	N/A	Preclinical	[192]
		Simvastatin	Preventing endothelial dysfunction and increasing eNOS	Direct endothelial protection	N/A	Preclinical	[193]
		SN34960	Reduces antigen-specific CD8⁺ T-cell responses and prevents sinusoidal perfusion failure and hepatocyte death	Indirect vascular protection	N/A	Preclinical	[194]
Chronic liver injury	Cirrhosis	Simvastatin	Decreasing HVPG; improving liver microcirculation and hepatocyte perfusion	Indirect vascular protection	N/A	Preclinical	[195]
		Simvastatin	Upregulates the KLF2–Nrf2 pathway, deactivates HSCs, and reduces liver fibrosis	Indirect vascular protection	N/A	Preclinical	[196]
		Simvastatin	Improves the HSC phenotype, hepatic endothelial dysfunction, and portal hypertension	Indirect vascular protection	N/A	Preclinical	[197]
		Simvastatin-loaded nanoparticles	Activates the KLF2–NO pathway and reduces oxidative stress and proinflammatory cytokines	Indirect vascular protection	N/A	Preclinical	[198]
		SC-560	Downregulates COX-, NF-κB-, and VEGF-mediated pathways	Indirect vascular protection	N/A	Preclinical	[199]
		Tetrahydrobiopterin	Ameliorating endothelial dysfunction and increasing NO bioavailability	Direct endothelial protection	N/A	Preclinical	[200]
		Tetrahydrobiopterin	Increasing eNOS activity and NO bioavailability	Direct vascular tone modulation	N/A	Preclinical	[201]
		Indomethacin, SC-560, and SQ-29548	Modulates NO release and limits hyperdynamic circulation	Direct endothelial protection	N/A	Preclinical	[202]
		AVE 9488	Ameliorates portal hypertension and upregulates hepatic eNOS	Direct endothelial protection	N/A	Preclinical	[203, 204]
		Dark chocolate	Blunting the postprandial increase in HVPG; ameliorating systemic hypotension	Indirect vascular protection	NCT01408966	Completed	[205]
		Fenofibrate	Improves hepatic endothelial dysfunction and the vasodilatory response	Indirect endothelial protection	N/A	Preclinical	[206]
		Recombinant MnSOD	Reduces oxidative stress and hepatic endothelial dysfunction	Direct endothelial protection	N/A	Preclinical	[207]
		Resveratrol	Inhibiting the proangiogenic response of HSCs and LSECs	Indirect + partial endothelial protection	N/A	Preclinical	[208]
		Obeticholic acid	Inhibiting both LSEC and Kupffer cell activation; inhibiting hepatic inflammation	Indirect endothelial protection	N/A	Preclinical	[209]
		Ad-KLF2	Improves hepatic endothelial dysfunction, upregulates the KLF2–Nrf2 pathway, deactivates HSCs, and reduces liver fibrosis	Direct LSEC reprogramming	N/A	Preclinical	[196]
		Atorvastatin and NCX 6560	Activating endothelial NOS and decreasing platelet/endothelial cell adhesion molecule-1	Direct + indirect endothelial protection	N/A	Preclinical	[210]
		siCOX1	Inhibiting over-activation of the COX-1/TXA_2_ pathways in LSECs; reducing portal pressure	Direct endothelial pathway modulation	N/A	Preclinical	[211]
		PX20606	Restoring eNOS activity and DDAH expression	Indirect endothelial protection	N/A	Preclinical	[212]
		Human amnion-derived stem cells	Improves hepatic microcirculatory function, reduces LSEC capillarization, promotes HSC deactivation, and decreases hepatic inflammation and oxidative stress	Indirect endothelial repair	N/A	Preclinical	[213]
		Aleglitazar	Inhibits primary HSC contraction, VEGF-induced LSEC migration, and angiogenesis	Indirect endothelial protection	N/A	Preclinical	[214]
		Lanifibranor	Improving LSEC and HSC phenotypes; ameliorating hepatic microvascular function; reducing hepatic inflammation	Indirect endothelial protection	N/A	Preclinical	[215]
		AICAR	Increasing p-eNOS expression and NO production	Direct endothelial protection	N/A	Preclinical	[216]
		Emricasan	Improves LSEC dysfunction and the phenotypes of hepatocytes, LSECs, HSCs, and macrophages	Indirect multi-cellular protection	N/A	Preclinical	[217]
		Palosuran	Enhancing eNOS-dependent HSC vasodilatation	Indirect vascular protection	N/A	Preclinical	[218]
		Statin-loaded delivery nanoparticles	Reducing liver fibrosis; lowering collagen expression; stimulating NO synthesis; decreasing LSEC capillarization	Direct LSEC reprogramming	N/A	Preclinical	[193]
	Acute-on-chronic liver failure	Simvastatin	Decreasing microvascular dysfunction and reducing liver inflammation	Direct endothelial protection	N/A	Preclinical	[219]
			Ameliorating microvascular dysfunction; upregulating liver eNOS and reducing liver inflammation and oxidative stress	Direct endothelial protection	N/A	Preclinical	[220]
HCC	HCC	Nano delivery of simvastatin	Alleviating LSEC capillarization; recruiting NKT cells through CXCL16	Direct LSEC/tumor endothelial reprogramming	N/A	Preclinical	[221]
		Durvalumab/anti-PD-L1 antibodies	Reinvigorate antitumor T-cell responses mainly through checkpoint blockade on tumor and immune cells; they are not LSEC-specific despite possible PD-L1 expression by LSECs	Unknown	NCT01853618	Completed	[222, 223]
		Regorafenib/cabozantinib	Broadly inhibit tumor neovascular and cancer-cell signaling but do not selectively restore LSEC fenestration or canonical LSEC identity	Unknown	NCT01774344	Completed	[224, 225]
		miR-3178 restoration	Restores a miRNA reduced in tumor endothelial cells and may normalize tumor-associated endothelial phenotype	Potential direct tumor endothelial reprogramming	N/A	Preclinical	[226]

*eNOS* endothelial nitric oxide synthase, *HCC* hepatocellular carcinoma, *HSCs* hepatic stellate cells, *KLF2* Krüppel-like factor 2, *LSECs* liver sinusoidal endothelial cells, *MMP* matrix metalloproteinase, *NKT cells* natural killer T cells, *NO* nitric oxide, *PD-L1* programmed death ligand 1, *S1PR1* sphingosine-1-phosphate receptor 1, *SOS* sinusoidal obstruction syndrome, *VEGF* vascular endothelial growth factor

#### Treatment for drug-induced liver injury

##### N-acetylcysteine

N-acetylcysteine is an established antidote for paracetamol-induced liver injury (Table 1). Dosing within a 2–10-h window after overdose replenishes glutathione consumed by paracetamol toxicity [227]. N-acetylcysteine donates sulfhydryl groups and thus plays a crucial role in replenishing intracellular glutathione [228]. It has been demonstrated that N-acetylcysteine inhibits the production of αV integrin, β3 integrin, and laminin in human LSECs injured by long-term high glucose stimulation and in ROS-mediated palmitate injury in cultured human LSECs [229]. Limitations in treatment timing and clinical response have prompted investigation of additional antioxidant compounds. For example, substances such as curcumin [230, 231], silymarin [232], α-lipoic acid [233], sulforaphane [234], zerumbone [235], hibiscus [236], acrocarpus fraxinifolius [237], sesame [238], resveratrol [208], aloe vera, artichoke leaf [239] and apigenin [240] reduce paracetamol-induced increases in inflammatory cytokines, lipid peroxidation, and aminotransferase levels in animal models. These are representative compounds evaluated for paracetamol-induced hepatotoxicity. Each of them functions in concert to reduce oxidative stress by replenishing glutathione, enhancing ROS-scavenging systems, and modulating antioxidant enzymes [241].

N-acetylcysteine has been demonstrated to increase the release of cGMP in LSECs from rats with obstructive jaundice; nonetheless, these findings suggest that protecting LSECs may help limit secondary hepatocyte injury in drug-induced liver injury. Lactoferrin, for example, has been shown to protect mouse LSECs against paracetamol-induced damage. Lactoferrin may attenuate paracetamol-induced LSEC damage and hepatic microcirculatory dysfunction by promoting protective Kupffer-cell-derived mediators [184].

##### Special treatment for SOS

Obstruction of hepatic sinusoids is the defining feature of sinusoidal obstruction syndrome (SOS), a form of toxic vascular liver injury [242]. SOS can follow exposure to cytotoxic chemotherapy or conditioning regimens used for hematopoietic stem cell transplantation (HSCT), with glutathione depletion, endothelial injury, coagulation activation, and increased vWF contributing to pathogenesis [243]. Initial centrilobular LSEC damage is followed by hepatocellular injury and HSC activation. Through platelet aggregation and a decrease in NO bioavailability, LSECs have been shown to play a significant role in the pathophysiology of SOS in rats [244]. In a preclinical model of SOS, NO expression is reduced. Recombinant thrombomodulin has also been proposed as a coagulation-inhibiting therapy for SOS. According to clinical research, anticoagulant medication is beneficial for SOS following HSCT [245]. Furthermore, TXA_2_ receptor agonism may also reduce LSEC damage in a preclinical model of SOS [189] (Table 1).

##### Special treatment for herbal-induced liver injury

Liver injury has also been linked to natural therapies, namely herbal and dietary supplements [246]. The concept of herb-induced liver injury and regulatory lists of potentially hepatotoxic herbal compounds have been reviewed elsewhere [247] (Table 1). Similar to drug-induced liver injury, herbal-induced liver injury can present with a wide spectrum of clinical symptoms, from severe liver failure to asymptomatic aberrant liver biochemistry [248]. While only pyrrolizidine alkaloids have been consistently and specifically linked to damage to the liver sinusoidal endothelium, it is conceivable that these compounds may also have an impact on LSEC viability given the general mechanisms impacted by a number of herbal and dietary supplements, including oxidative stress, apoptosis, and immune function [99]. However, LSEC vulnerability to liver damage caused by herbal remedies has not been explicitly verified, warranting further investigation. Elucidating the precise functions of LSECs in the initiation and persistence of drug-induced liver injury relies on novel tools such as liver sinusoid-mimicking fluidic devices, which facilitate the toxicity assessment and therapeutic exploration of liver injury caused by drugs, herbal remedies, and dietary supplements.

##### Special treatment for acute bacterial and viral infections

LSECs’ function as APCs has already been examined. LSECs are generally regarded as non-professional APCs that can cross-present antigen and modulate cytotoxic and helper T-cell responses; however, their relatively low MHC class II and co-stimulatory molecule expression can limit conventional T-cell activation. Commensal bacteria can engage MYD88-dependent signaling that enables LSECs to regulate pericellular chemokine gradients under steady-state conditions. LSECs can cross-present exogenous antigens to CD8^+^ T cells and induce antigen-specific tolerance [249] (Table 1).

It has also been discovered that bacterial and viral infections target LSECs during acute liver failure, altering their tolerogenic steady state to increase inflammatory activity [250]. By expressing adhesion molecules such as ICAM1 and vascular adhesion protein-1 (VAP1, also known as AOC3), LSECs can recruit leukocytes [251]. Inflammation upregulates multiple adhesion molecules such as VCAM1 and PECAM1, alongside enhanced ICAM1 production in LSECs [252].

LSECs regulate signaling via TLR9 in reaction to common bacterial CpG oligonucleotides. Murine LSECs express functional TLR9 and respond to CpG oligonucleotides, providing a route for sensing microbial DNA [253]. *Pseudomonas aeruginosa* is one of the most prevalent nosocomial bacteria that cause opportunistic infections in liver transplant recipients. Structural alterations in LSECs have also been reported in response to bacillary infections, including *Bartonella speci*es. These cells are important for sepsis-associated tissue lipoprotein lipase inhibition and increased hepatic triglyceride delivery, as LSEC loss of porosity results in reduced liver absorption of lipoproteins and chylomicrons, thereby contributing to hyperlipidemia [254]. During sepsis, gram-negative bacteria produce lipopolysaccharide, which is quickly removed from the bloodstream. LSECs contribute to this clearance via their interaction with lipopolysaccharide mediated by HDL. This mechanism may be related to scavenger receptor B1, which is highly expressed by mouse LSECs [255]. LSECs express constitutive CXCR6 ligand CXCL16 in response to *Listeria monocytogenes*, which indirectly aids in the buildup of CXCR6^+^ cytotoxic T cells in mouse livers [256].

LSECs facilitate the lysosomal degradation of internalized bacteriophages, which are increasingly recognized as relevant components of hepatic antiviral immunity. Following exposure to palmitoyl-3-cysteine-serine-lysine-4, TLR1/TLR2 activation in LSECs may counteract local T-cell suppression. LSECs can also promote antiviral antigen-specific CD8^+^ T-cell responses in this setting [257]. Under in *vitro* conditions, activation of antigen-specific T-cell responses against HBV is triggered by TLR3 and nucleotide-binding oligomerization domain 1 (NOD1) receptors in mouse LSECs. The paradox between extremely effective liver targeting and ineffective viral absorption by cultured hepatocytes may be explained by virus transcytosis across LSECs, as suggested by the initial scavenging of HBV by LSECs [258].

LSECs bind HCV envelope glycoprotein E2 via the C-type lectin L-SIGN. However, it remains unclear whether this interaction drives HCV lysosomal clearance or facilitates trans-infection toward hepatocytes [259]. According to a study, capillarization is only promoted during the early phases of fibrosis, and LSECs retain aspects of their phenotype during chronic HCV infection [260].

Cytomegalovirus (CMV) can also infect LSECs. Trafficking molecules, including ICAM1 and CXCL10, are upregulated in CMV-infected LSECs. Liver inflammation results from the recruitment of effector CD4^+^ T cells and the promotion of functional activation of various T cell subsets [261]. LSECs may bind malaria sporozoites, probably through endothelial proteoglycans, and facilitate their entry into the hepatic parenchyma, thereby playing a role in parasitic *Plasmodium* infections beyond bacterial and viral infections [262].

### Treatment for chronic liver diseases

In recent years, a number of treatment strategies targeting LSECs have been explored in light of their potential role in chronic liver diseases (Table 1). Statins have been shown to exert positive effects on endothelial dysfunction, fibrogenesis, and portal hypertension in several preclinical studies [263, 264]. Through the activation of KLF2 expression in LSECs, simvastatin lowers portal hypertension and endothelial dysfunction while promoting positive paracrine effects in HSCs [196]. However, statins are pleiotropic agents that also directly modulate hepatocyte lipid metabolism and exert antifibrotic effects on HSCs through mechanisms independent of LSECs [263, 265]. Consequently, the vascular benefits observed with systemic statin therapy reflect both direct endothelial protection and indirect parenchymal metabolic improvement. While the direct molecular mechanisms remain to be fully elucidated, simvastatin treatment in *vivo* has been shown to improve endothelial porosity in aged cirrhotic mice, suggesting a restorative effect on fenestrae. Simvastatin therapy in *vitro* does not reduce fenestrae loss in aged capillarized LSECs. However, simvastatin treatment in *vivo* improves endothelial porosity in aged cirrhotic mice, which in turn can reduce portal hypertension and microvascular dysfunction [265]. In addition, statin-loaded nanoparticles alleviate hepatic fibrosis, suppress collagen expression, and stimulate NO synthesis during chronic liver disease progression [193, 266].

In individuals with cirrhosis, either short-term or long-term simvastatin medication also reduces portal pressure without affecting hepatic blood flow, which may reflect decreased intrahepatic vascular resistance associated with enhanced NO bioavailability [267]. Simvastatin inhibits the proinflammatory response and further development of microvascular dysfunction in preclinical late-stage cirrhosis, thus preventing the detrimental consequences of acute-on-chronic liver failure [268]. Further clinical studies are needed to define its efficacy and safety in advanced chronic liver disease.

Agents that modulate eNOS activity and NO generation, such as AVE 9488 and tetrahydrobiopterin, have also been investigated for reducing LSEC dysfunction in cirrhotic mice, in addition to statins [203]. Likewise, lowering vasoconstriction levels is considered another effective treatment approach for improving LSEC performance. According to the groundbreaking research on the hepatic COX1-TXA_2_ axis, carbon tetrachloride-cirrhotic animals treated with small interfering RNA against LSEC-specific COX1 and TXA_2_ exhibit decreased portal pressure and liver fibrosis [211].

In order to alleviate chronic liver impairments and their repercussions, antioxidant compounds that target LSECs have been suggested as prospective treatment approaches. Substances that have improved the LSEC phenotype and function in preclinical models or in patients include resveratrol, docosahexaenoic acid triglyceride, tofogliflozin, dark chocolate, and a recombinant form of manganese superoxide dismutase. These interventions have improved fibrosis, microvascular dysfunction, or portal hypertension in the respective experimental or clinical settings [106, 125, 269].

Endothelial vasoprotection is provided by KLF2, a nuclear transcription factor sensitive to shear stress. As an approach to induce vasoprotective target genes, KLF2 expression has been increased in the liver endothelium in preclinical models of chronic liver disease. However, KLF2 effects are context-dependent; in regenerative settings, endothelial KLF2 can restrain hepatocyte proliferation through activin A [270]. Therefore, a method to further activate the KLF2 pathway using statins, resveratrol, or microRNAs for protecting endothelium and improving chronic liver disorders has been proposed. In addition to KLF2, other transcription factors have been explored as possible treatment approaches for modifying the LSEC phenotype. In cirrhotic animals, farnesoid X receptor agonists, including obeticholic acid and PX20606, may reactivate eNOS activity, which in turn can alleviate endothelial dysfunction and portal hypertension [212, 271]. However, FXR is predominantly expressed in hepatocytes and intestinal epithelial cells, with only modest levels in LSECs [271]. FXR agonists such as obeticholic acid may improve LSEC dysfunction indirectly through bile acid homeostasis, hepatocyte metabolic regulation, and reduced inflammatory signaling, though direct LSEC reprogramming remains insufficiently established. Therefore, beyond its direct effects on LSECs, obeticholic acid may indirectly alleviate endothelial dysfunction by attenuating hepatocellular oxidative stress and stabilizing bile acid homeostasis. Obeticholic acid has also been investigated for MASH. Finally, endothelial dysfunction in cirrhotic mouse livers is attenuated by fenofibrate [206] or aleglitazar, a dual peroxisome proliferator-activated receptor (PPAR)α/PPARγ agonist. Additionally, there is a decrease in proangiogenic markers in primary isolated cirrhotic rat LSECs treated with aleglitazar [214]. Nevertheless, PPARα is highly enriched in hepatocytes, where it regulates mitochondrial fatty acid oxidation, whereas PPARγ is predominantly expressed in HSCs and macrophages [206, 214]. Thus, PPAR agonists may improve sinusoidal endothelial dysfunction through combined actions on hepatocytes, HSCs, macrophages, and metabolic inflammation. Therefore, their beneficial effects on LSECs should be interpreted as indirect or multi-cellular endothelial protection rather than selective LSEC targeting. However, the most profound therapeutic opportunity lies in actively reprogramming the capillarized endothelium rather than protecting it transiently. Future strategies must target master transcriptional and epigenetic switches to durably reverse LSEC dedifferentiation, restoring scavenger function, immune tolerance, and vascular homeostasis as definitive therapy for chronic liver disease.

### Treatment for HCC

A common therapeutic approach for HCC is the use of agents that inhibit angiogenesis, cell proliferation, survival, or motility-related signaling pathways (Table 1). For example, durvalumab and other anti-PD-L1 antibodies have been investigated in HCC. While LSECs express PD-L1 and may contribute to local immune suppression, these antibodies primarily target PD-L1 on tumor cells and myeloid antigen-presenting cells to restore T-cell-mediated cytotoxicity. Their clinical efficacy is not predicated on selective LSEC targeting but on comprehensive checkpoint blockade within the tumor microenvironment [150, 222]. Additionally, miR-3178 expression is decreased in tumor endothelial cells compared to normal LSECs, and restoring its expression may represent a potential therapeutic strategy for HCC. This clarifies that the goal is to replenish the lost miRNA [226]. It has been shown that tyrosine kinase inhibitors such as regorafenib [224] and cabozantinib [225] broadly inhibit tumor neovascular signaling yet fail to selectively restore LSEC fenestration or canonical LSEC phenotypic identity. However, these agents broadly target pathological tumor neovasculature and cancer cells rather than specifically reprogramming LSECs, as HCC-associated endothelial cells have undergone capillarization and largely lost canonical LSEC markers and fenestration [24, 161]. Hepatocytes from patients with HBV infection and HCC present a disease-associated immunopeptidome that may shape antitumor T-cell recognition [272]. Clinical development in HCC has included CTLA4 blockade with tremelimumab, including early combination with tumor ablation, as well as PD1/PD-L1-directed antibodies such as tislelizumab and camrelizumab [223, 273]. Phase III results have been published for tislelizumab in RATIONALE-301 and for camrelizumab plus rivoceranib in CARES-310 (NCT03412773 and NCT02989922) [274–276]. Notably, these monoclonal antibodies are T-cell-directed therapies, and they do not selectively target LSECs. While LSEC-expressed checkpoint ligands may contribute to local immune regulation, the clinical evidence cited here supports systemic checkpoint blockade rather than selective LSEC targeting [223]. Nevertheless, the ultimate therapeutic goal is not sustained signaling inhibition, but recovery of the physiological LSEC phenotype. Given that angiogenesis and immune evasion are mechanistically coupled through tumor-conditioned LSECs, future therapies should move beyond parallel anti-angiogenic and checkpoint-blockade strategies toward endothelial reprogramming approaches that normalize tumor vasculature while restoring antigen presentation and T-cell permissiveness. Reprogramming tumor endothelial cells to re-express stabilins, re-establish fenestrae, and restore cross-presentation capacity may convert the immune-privileged HCC microenvironment into an immune-permissive niche.

## Conclusion and perspectives

This review’s cumulative evidence supports a fundamental paradigm shift, redefining LSECs from passive bystanders in liver disease to central regulators of hepatic homeostasis [35]. Recent advances in multi-omics technologies, particularly single-cell RNA sequencing and spatially resolved proteogenomic atlases, have facilitated the characterization of diverse LSEC subpopulations with unprecedented precision [277]. These methodologies have deciphered zone-dependent vascular signaling pathways and identified distinct molecular addresses such as Oit3 for quiescent LSECs, PLVAP/ACKR1 for scar-associated endothelial cells in fibrotic septa, as well as S100A16 for tumor-associated endothelial cells. They offer ligand-directed delivery opportunities to selectively engage pathogenic subsets while sparing homeostatic ones. Therefore, the major contribution of these technologies is not only higher-resolution cell annotation but also the ability to map pathogenic endothelial states onto spatial niches, signaling circuits, and therapeutic entry points. By anchoring therapeutic design in the subpopulation architecture revealed by multi-omics, the field can transition from phenomenological description and palliative endothelial protection to precise phenotypic reprogramming, actively restoring the quiescent, tolerogenic, and metabolically active LSEC phenotype rather than merely suppressing pathological activation.

Despite these compelling mechanistic insights, substantial research gaps remain. Further investigations are required to transition current descriptive associations toward definitive mechanistic causality and precise therapeutic application. Foremost among these is the persistent lack of truly LSEC-specific molecular identifiers and genetically tractable models. Although pan-endothelial drivers such as Cdh5-Cre cannot distinguish LSECs from other endothelial populations, Oit3-CreERT2 has emerged as an LSEC-enriched lineage-tracing tool [12]. Recent single-cell analyses of regenerating human liver have identified multiple molecularly distinct LSEC subpopulations and condition-dependent alterations in endothelial zonation [278]. Therefore, the labeling coverage and stability of Oit3-CreERT2 across lobular zones and disease states require further validation. However, these markers are not specific to LSECs: MRC1/CD206 is shared by vascular endothelial and immune-cell populations, whereas LYVE1 is also expressed by hepatic lymphatic endothelial cells [279]. The absence of exclusive surface markers increases the risk of off-target drug distribution to extrahepatic sites, particularly the pulmonary and renal vascular beds, potentially triggering pulmonary inflammation or glomerular injury. Therefore, future research must prioritize the discovery and validation of truly LSEC-restricted genetic tools and surface markers, leveraging single-cell-derived signatures and spatial transcriptomics to overcome this fundamental technical bottleneck.

A second unresolved challenge lies in elucidating context-dependent signaling paradoxes and establishing definitive causal evidence of LSEC dysfunction as a disease-initiating event. The VEGF signaling pathway exemplifies this ambiguity: physiological VEGF-A/VEGFR2 signaling supports fenestration and eNOS activity, whereas chronic injury changes ligand exposure, receptor balance, hypoxia, and inflammatory signaling; the precise contributions of VEGFR1, sustained ERK activation, and eNOS uncoupling remain unresolved [280]. It remains to be definitively verified whether hepatic capillarization arises from VEGFR1 decoy signaling, excessive VEGFR2 activation, or dose-dependent shifts in receptor subtype usage. Furthermore, although LSEC capillarization consistently precedes overt parenchymal damage in metabolic stress, toxic exposure, and viral infection, most evidence is sourced from correlative observations in pan-endothelial models rather than lineage-specific causal frameworks. Refined genetic approaches—ideally utilizing LSEC-specific drivers once available—should thus be employed to demonstrate that targeted prevention of LSEC dedifferentiation can halt or reverse downstream fibrogenesis and immune dysregulation, thereby more rigorously defining when and how LSEC dysfunction functions as an initiating driver, amplifier, or sustaining node in disease progression [35]. Compounding this challenge, primary LSECs undergo rapid dedifferentiation when cultured in static 2D systems, losing their fenestrae and scavenger functions within days [281]. This biological fragility induces a severe bottleneck for mechanism validation and high-throughput drug screening. Therefore, the widespread adoption of dynamic 3D microphysiological systems (liver-on-a-chip) that incorporate fluidic shear stress, co-culture with parenchymal cells, and tunable matrix stiffness. By accurately recapitulating mechanotransduction pathways such as the shear-stress-dependent KLF2-eNOS axis, these advanced models may help bridge the translational gap between basic discovery and clinical efficacy.

From a translational perspective, the development of precision reprogramming platforms encounters substantial biological and technical barriers, constituting a third critical research gap. Systemic drug administration often results in non-targeted delivery to endothelial cells in other organs, and the dynamic blood flow patterns in the portal venous system affect drug residence duration and sinusoidal interaction [105]. These challenges are further compounded by disease-driven alterations in LSEC phenotypes: during capillarization, downregulation of scavenger receptors (STAB1, STAB2, CD32b) reduces ligand-mediated uptake, thereby decreasing targeting efficiency at the critical therapeutic window. Additionally, the narrow fenestrae structure (approximately 100–150 nm) imposes physical limitations on carrier size, while basement membrane deposition and collagenous septa in advanced fibrosis create a formidable barrier that must be overcome for effective nanoparticle penetration [282]. Critical research questions therefore include designing phenotype-adaptive delivery platforms that bypass or enzymatically degrade the capillarized barrier, optimizing nanoparticle pharmacokinetics to achieve sufficient LSEC engagement without pulmonary or renal sequestration, and achieving functional re-education—restoring fenestration, KLF2-driven NO signaling, and tolerogenic scavenger function—through direct delivery of transcription factor activators or epigenetic editors to LSECs.

Looking forward, the ultimate therapeutic goal must transcend simple inhibition strategies, including conventional anti-angiogenic or anti-inflammatory approaches, and instead embrace therapeutic reprogramming of the angiocrine-immune-fibrotic axis as the fundamental intervention principle in liver disease. Rather than targeting hepatocytes, HSCs, Kupffer cells, or inflammation in isolation, future strategies should aim to reset the entire LSEC-centered axis by simultaneously restoring endothelial homeostasis, restraining HSC activation, and reprogramming Kupffer cell-driven inflammatory amplification. By restoring fenestral structure, rescuing antigen cross-presentation function, balancing angiocrine secretion, and reactivating key transcriptional regulators such as KLF2 and GATA4, targeted LSEC reprogramming represents a transformative strategy to dismantle immune privilege, normalize tumor vasculature, and block the initiation of liver fibrogenesis. Potential modalities include PPARγ agonists to maintain LSEC differentiation, epigenetic editing to reverse capillarization, and targeted delivery of microRNAs to fine-tune inflammatory responses without compromising host defense. Confronting the identified gaps in LSEC-specific tools, mechanistic causality, and precision delivery will elevate LSEC biology from descriptive mapping to mechanistically grounded, spatially resolved, and functionally validated intervention. This approach represents not only a potential frontier for treating liver disease but also a framework for refining vascular niche-targeted strategies against systemic metabolic, inflammatory, and neoplastic disorders.

In summary, redefining LSECs as the central regulators of hepatic health and disease marks a crucial turning point in hepatology. The successful clinical translation of this knowledge hinges on moving beyond the mere suppression of downstream symptoms to the active, precision reprogramming of the LSEC microenvironment. The integration of advanced 3D microphysiological models for mechanistic investigation and capillarization-targeted nanomedicine strategies holds considerable potential to resolve persistent translational bottlenecks. Ultimately, restoring the unique angiocrine and tolerogenic functions of LSECs offers a highly promising, unifying therapeutic strategy to halt or even reverse the progression of acute liver injuries, chronic fibrotic diseases, and HCC. Thus, the central challenge for future LSEC research lies not merely in characterizing endothelial abnormalities in diseased liver, but in defining when, where, and how LSEC rheostat failure initiates disease trajectories—and how this switch can be therapeutically reset.

## Data Availability

Not applicable.

## Abbreviations

AAV: Adeno-associated virus
ACKR1: Atypical chemokine receptor 1
ALK1: Activin receptor-like kinase 1
ANGPT2: Angiopoietin-2
AP-1: Activating protein-1
APCs: Antigen-presenting cells
ATP: Adenosine triphosphate
BMP4: Bone morphogenetic protein 4
CAR-T: Chimeric antigen receptor T cell
CB2: Cannabinoid type 2
CCL2: C-C motif chemokine ligand 2
COX: Cyclooxygenase
cGMP: Cyclic guanosine monophosphate
CMV: Cytomegalovirus
CTGF: Connective tissue growth factor
CTLA4: Cytotoxic T lymphocyte antigen 4
CXCL1: CXC motif chemokine ligand 1
CXCL16: CXC motif chemokine ligand 16
CXCR6: CXC chemokine receptor 6
DAMPs: Damage-associated molecular patterns
ECM: Extracellular matrix
eNOS: Endothelial nitric oxide synthase
ERG: ETS-related gene
ET-1: Endothelin-1
F8: Factor VIII
FcγRIIb: Fc gamma receptor IIb
GLS1: Glutaminase 1
HBV: Hepatitis B virus
HCC: Hepatocellular carcinoma
HCV: Hepatitis C virus
HIF-1α: Hypoxia-inducible factor 1-alpha
HMG-CoA: 3-Hydroxy-3-methylglutaryl-coenzyme A
HMGB1: High-mobility group box 1
HO-1: Heme oxygenase-1
HSCs: Hepatic stellate cells
HSCT: Hematopoietic stem cell transplantation
HVPG: Hepatic venous pressure gradient
ICAM1: Intercellular adhesion molecule 1
IL: Interleukin
KLF2: Krüppel-like factor 2
LSECs: Liver sinusoidal endothelial cells
L-SIGN: Liver/lymph node-specific ICAM-3-grabbing non-integrin
LYVE1: Lymphatic vessel endothelial hyaluronan receptor 1
MASH: Metabolic dysfunction-associated steatohepatitis
MASLD: Metabolic dysfunction-associated steatotic liver disease
MDSCs: Myeloid-derived suppressor cells
MetALD: Metabolic dysfunction and alcohol-related liver disease
MHC: Major histocompatibility complex
MMP: Matrix metalloproteinase
MYD88: Myeloid differentiation primary response 88
NF-κB: Nuclear factor-kappa B
NK: Natural killer
NKT: Natural killer T cell
NO: Nitric oxide
NOD1: Nucleotide-binding oligomerization domain 1
Oit3: Oncoprotein-induced transcript 3
oxLDL: Oxidized low-density lipoproteins
PAF: Platelet-activating factor
PDGF: Platelet-derived growth factor
p-eNOS: Phosphorylated eNOS
PD1: Programmed cell death protein 1
PD-L1: Programmed death ligand 1
PECAM1: Platelet endothelial cell adhesion molecule-1
p-ERK1/2: Phosphorylated ERK1/2
PLVAP: Plasmalemma vesicle-associated protein
PPAR: Peroxisome proliferator-activated receptor
PRRs: Pattern recognition receptors
ROS: Reactive oxygen species
sGC: Soluble guanylate cyclase
SOS: Sinusoidal obstruction syndrome
STAB: Stabilin
TGF-β: Transforming growth factor-beta
TLR: Toll-like receptor
TNF: Tumor necrosis factor
Treg cells: Regulatory T cells
TXA2: Thromboxane A2
TXNIP: Thioredoxin-interacting protein
VAP-1: Vascular adhesion protein-1 (AOC3)
VCAM1: Vascular cell adhesion molecule 1
VEGFR: Vascular endothelial growth factor receptor
VLDL: Very low-density lipoprotein
vWF: Von Willebrand factor
YAP/TAZ: Yes-associated protein/transcriptional coactivator with PDZ-binding motif

## References

[1] He Q, He W, Dong H, Guo Y, Yuan G, Shi X, et al. Role of liver sinusoidal endothelial cell in metabolic dysfunction-associated fatty liver disease. Cell Commun Signal. 2024;22(1):346. 10.1186/s12964-024-01720-9. 38943171 10.1186/s12964-024-01720-9 PMC11214243

[2] Qu J, Wang L, Li Y, Li X. Liver sinusoidal endothelial cell: an important yet often overlooked player in the liver fibrosis. Clin Mol Hepatol. 2024;30(3):303–25. 10.3350/cmh.2024.0022. 38414375 10.3350/cmh.2024.0022 PMC11261236

[3] Zhou J, Wang J, Zhang L, Zhang C, Tian C. Defenestration of liver sinusoidal endothelial cells: the trigger of liver fibrosis. Pharmaceuticals (Basel). 2025;18(6):893. 10.3390/ph18060893. 40573288 10.3390/ph18060893 PMC12196518

[4] Kremer KN, Khammash HA, Miranda AM, Rutt LN, Twardy SM, Anton PE, et al. Liver sinusoidal endothelial cells regulate the balance between hepatic immunosuppression and immunosurveillance. Front Immunol. 2025;15:1497788. 10.3389/fimmu.2024.1497788. 39896805 10.3389/fimmu.2024.1497788 PMC11782242

[5] Wang SH, Serr I, Digigow R, Metzler B, Surnov A, Gottwick C, et al. Nanoparticle platform preferentially targeting liver sinusoidal endothelial cells induces tolerance in CD4+ T cell-mediated disease models. Front Immunol. 2025;16:1542380. 10.3389/fimmu.2025.1542380. 40165970 10.3389/fimmu.2025.1542380 PMC11955608

[6] Gottwick C, Averhoff P, Casar C, Liebig LA, Pilz SM, Haas V, et al. Antigen-specific tolerance and control of autoimmunity effected by liver sinusoidal endothelial cells is unimpaired in liver fibrosis. Front Immunol. 2026;17:1834595. 10.3389/fimmu.2026.1834595. 42164495 10.3389/fimmu.2026.1834595 PMC13183527

[7] Minciuna I, Taru MG, Procopet B, Stefanescu H. The interplay between liver sinusoidal endothelial cells, platelets, and neutrophil extracellular traps in the development and progression of metabolic dysfunction-associated steatotic liver disease. J Clin Med. 2024;13(5):1406. 10.3390/jcm13051406. 38592258 10.3390/jcm13051406 PMC10932189

[8] Yu M, Li X, Xu L, Zheng C, Pan W, Chen H, et al. Neutrophil extracellular traps induce intrahepatic thrombotic tendency and liver damage in cholestatic liver disease. Hepatol Commun. 2024;8(8):e0513. 10.1097/HC9.0000000000000513. 39101776 10.1097/HC9.0000000000000513 PMC11299992

[9] Meyer J, Balaphas A, Fontana P, Morel P, Robson SC, Sadoul K, et al. Platelet interactions with liver sinusoidal endothelial cells and hepatic stellate cells lead to hepatocyte proliferation. Cells. 2020;9(5):1243. 10.3390/cells9051243. 32443494 10.3390/cells9051243 PMC7290338

[10] Winkler M, Staniczek T, Kurschner SW, Schmid CD, Schonhaber H, Cordero J, et al. Endothelial GATA4 controls liver fibrosis and regeneration by preventing a pathogenic switch in angiocrine signaling. J Hepatol. 2021;74(2):380–93. 10.1016/j.jhep.2020.08.033. 32916216 10.1016/j.jhep.2020.08.033

[11] Zapotoczny B, Szafranska K, Kus E, Braet F, Wisse E, Chlopicki S, et al. Tracking fenestrae dynamics in live murine liver sinusoidal endothelial cells. Hepatology. 2019;69(2):876–88. 10.1002/hep.30232. 30137644 10.1002/hep.30232

[12] Li ZW, Ruan B, Yang PJ, Liu JJ, Song P, Duan JL, et al. Oit3, a promising hallmark gene for targeting liver sinusoidal endothelial cells. Signal Transduct Target Ther. 2023;8(1):344. 10.1038/s41392-023-01621-2. 37696816 10.1038/s41392-023-01621-2 PMC10495338

[13] Yang T, Gu Z, Feng J, Shan J, Qian C, Zhuang N. Non-parenchymal cells: key targets for modulating chronic liver diseases. Front Immunol. 2025;16:1576739. 10.3389/fimmu.2025.1576739. 40568596 10.3389/fimmu.2025.1576739 PMC12187809

[14] Schurich A, Berg M, Stabenow D, Böttcher J, Kern M, Schild HJ, et al. Dynamic regulation of CD8 T cell tolerance induction by liver sinusoidal endothelial cells. J Immunol. 2010;184(8):4107–14. 10.4049/jimmunol.0902580. 20212092 10.4049/jimmunol.0902580

[15] Zurawska G, Sas Z, Jonczy A, Mahadeva R, Slusarczyk P, Chwalek M, et al. Liver sinusoidal endothelial cells constitute a major route for hemoglobin clearance. EMBO Rep. 2026;27(3):598–628. 10.1038/s44319-025-00673-5. 41495467 10.1038/s44319-025-00673-5 PMC12895045

[16] Hu J, Srivastava K, Wieland M, Runge A, Mogler C, Besemfelder E, et al. Endothelial cell-derived angiopoietin-2 controls liver regeneration as a spatiotemporal rheostat. Science. 2014;343(6169):416–9. 10.1126/science.1244880. 24458641 10.1126/science.1244880

[17] Manco R, Moliterni C, Neirynck G, Claus R, De Rudder M, Picalausa C, et al. Liver sinusoidal endothelial cells and laminin dictate cholangiocytes’ fate in chronic liver disease. J Hepatol. 2026;85(2):257–70. 10.1016/j.jhep.2026.03.022. 41905508 10.1016/j.jhep.2026.03.022

[18] Puri M, Sonawane S. Liver sinusoidal endothelial cells in the regulation of immune responses and fibrosis in metabolic dysfunction-associated fatty liver disease. Int J Mol Sci. 2025;26(9):3988. 10.3390/ijms26093988. 40362227 10.3390/ijms26093988 PMC12071881

[19] Yang Z, Li QT, Yue ZS, Li ZW, Zou P, Ling YW, et al. Liver sinusoidal endothelial TGF-beta signaling accelerates partial endothelial-mesenchymal transition and MASH through Notch. JHEP Rep. 2026;8(4):101784. 10.1016/j.jhepr.2026.101784. 41861675 10.1016/j.jhepr.2026.101784 PMC13019540

[20] Du W, Wang L. The crosstalk between liver sinusoidal endothelial cells and hepatic microenvironment in NASH related liver fibrosis. Front Immunol. 2022;13:936196. 10.3389/fimmu.2022.936196. 35837401 10.3389/fimmu.2022.936196 PMC9274003

[21] Velliou RI, Legaki AI, Nikolakopoulou P, Vlachogiannis NI, Chatzigeorgiou A. Liver endothelial cells in NAFLD and transition to NASH and HCC. Cell Mol Life Sci. 2023;80(11):314. 10.1007/s00018-023-04966-7. 37798474 10.1007/s00018-023-04966-7 PMC11072568

[22] Shetty S, Lalor PF, Adams DH. Liver sinusoidal endothelial cells - gatekeepers of hepatic immunity. Nat Rev Gastroenterol Hepatol. 2018;15(9):555–67. 10.1038/s41575-018-0020-y. 29844586 10.1038/s41575-018-0020-y PMC7096836

[23] McConnell MJ, Kostallari E, Ibrahim SH, Iwakiri Y. The evolving role of liver sinusoidal endothelial cells in liver health and disease. Hepatology. 2023;78(2):649–69. 10.1097/HEP.0000000000000207. 36626620 10.1097/HEP.0000000000000207 PMC10315420

[24] Poisson J, Lemoinne S, Boulanger C, Durand F, Moreau R, Valla D, et al. Liver sinusoidal endothelial cells: physiology and role in liver diseases. J Hepatol. 2017;66(1):212–27. 10.1016/j.jhep.2016.07.009. 27423426 10.1016/j.jhep.2016.07.009

[25] Su T, Yang Y, Lai S, Jeong J, Jung Y, McConnell M, et al. Single-cell transcriptomics reveals zone-specific alterations of liver sinusoidal endothelial cells in cirrhosis. Cell Mol Gastroenterol Hepatol. 2021;11(4):1139–61. 10.1016/j.jcmgh.2020.12.007. 33340713 10.1016/j.jcmgh.2020.12.007 PMC7903131

[26] Sørensen KK, Simon-Santamaria J, McCuskey RS, Smedsrød B. Liver sinusoidal endothelial cells. Compr Physiol. 2015;5(4):1751–74. 10.1002/cphy.c140078. 26426467 10.1002/cphy.c140078

[27] Li R, Oteiza A, Sorensen KK, McCourt P, Olsen R, Smedsrod B, et al. Role of liver sinusoidal endothelial cells and stabilins in elimination of oxidized low-density lipoproteins. Am J Physiol Gastrointest Liver Physiol. 2011;300(1):G71–81. 10.1152/ajpgi.00215.2010. 21030611 10.1152/ajpgi.00215.2010 PMC3025507

[28] Hammoutene A, Rautou PE. Role of liver sinusoidal endothelial cells in non-alcoholic fatty liver disease. J Hepatol. 2019;70(6):1278–91. 10.1016/j.jhep.2019.02.012. 30797053 10.1016/j.jhep.2019.02.012

[29] Miyao M, Kotani H, Ishida T, Kawai C, Manabe S, Abiru H, et al. Pivotal role of liver sinusoidal endothelial cells in NAFLD/NASH progression. Lab Invest. 2015;95(10):1130–44. 10.1038/labinvest.2015.95. 26214582 10.1038/labinvest.2015.95

[30] Greuter T, Yaqoob U, Gan C, Jalan-Sakrikar N, Kostallari E, Lu J, et al. Mechanotransduction-induced glycolysis epigenetically regulates a CXCL1-dominant angiocrine signaling program in liver sinusoidal endothelial cells in vitro and in vivo. J Hepatol. 2022;77(3):723–34. 10.1016/j.jhep.2022.03.029. 35421427 10.1016/j.jhep.2022.03.029 PMC9391258

[31] Wu X, Shu L, Zhang Z, Li J, Zong J, Cheong LY, et al. Adipocyte fatty acid binding protein promotes the onset and progression of liver fibrosis via mediating the crosstalk between liver sinusoidal endothelial cells and hepatic stellate cells. Adv Sci (Weinh). 2021;8(11):e2003721. 10.1002/advs.202003721. 34105268 10.1002/advs.202003721 PMC8188197

[32] Gao J, Lan T, Kostallari E, Guo Y, Lai E, Guillot A, et al. Angiocrine signaling in sinusoidal homeostasis and liver diseases. J Hepatol. 2024;81(3):543–61. 10.1016/j.jhep.2024.05.014. 38763358 10.1016/j.jhep.2024.05.014 PMC11906189

[33] Desroches-Castan A, Tillet E, Ricard N, Ouarne M, Mallet C, Belmudes L, et al. Bone morphogenetic protein 9 is a paracrine factor controlling liver sinusoidal endothelial cell fenestration and protecting against hepatic fibrosis. Hepatology. 2019;70(4):1392–408. 10.1002/hep.30655. 30964206 10.1002/hep.30655

[34] Zhao JB, Luo GQ, Wu ZH, Lin JY, Zhang CH, Wu GB, et al. Spermidine-based carbon quantum dots alleviate liver sinusoidal endothelial dysfunction by inducing LSEC-derived NO to ameliorate hepatic fibrosis and portal hypertension. Adv Healthc Mater. 2026;15(14):e05055. 10.1002/adhm.202505055. 41614599 10.1002/adhm.202505055

[35] Gracia-Sancho J, Caparros E, Fernandez-Iglesias A, Frances R. Role of liver sinusoidal endothelial cells in liver diseases. Nat Rev Gastroenterol Hepatol. 2021;18(6):411–31. 10.1038/s41575-020-00411-3. 33589830 10.1038/s41575-020-00411-3

[36] Ding BS, Cao Z, Lis R, Nolan DJ, Guo P, Simons M, et al. Divergent angiocrine signals from vascular niche balance liver regeneration and fibrosis. Nature. 2014;505(7481):97–102. 10.1038/nature12681. 24256728 10.1038/nature12681 PMC4142699

[37] Gao J, Zuo B, He Y. Liver sinusoidal endothelial cells as potential drivers of liver fibrosis (review). Mol Med Rep. 2024;29(3):40. 10.3892/mmr.2024.13164. 38240102 10.3892/mmr.2024.13164 PMC10828992

[38] Ramachandran P, Matchett KP, Dobie R, Wilson-Kanamori JR, Henderson NC. Single-cell technologies in hepatology: new insights into liver biology and disease pathogenesis. Nat Rev Gastroenterol Hepatol. 2020;17(8):457–72. 10.1038/s41575-020-0304-x. 32483353 10.1038/s41575-020-0304-x

[39] Felli E, Nulan Y, Ortega-Ribera M, Fernandez-Iglesias A, Gracia-Sancho J. The role of liver sinusoidal endothelial cells in liver diseases: key players in health and pathology. J Hepatol. 2026;84(3):655–72. 10.1016/j.jhep.2025.10.017. 42133683 10.1016/j.jhep.2025.10.017

[40] Ramachandran P, Dobie R, Wilson-Kanamori JR, Dora EF, Henderson BEP, Luu NT, et al. Resolving the fibrotic niche of human liver cirrhosis at single-cell level. Nature. 2019;575(7783):512–8. 10.1038/s41586-019-1631-3. 31597160 10.1038/s41586-019-1631-3 PMC6876711

[41] Li HY, Gao YX, Wu JC, Li JZ, Fu SW, Xu MY. Single-cell transcriptome reveals a novel mechanism of c-Kit(+)-liver sinusoidal endothelial cells in NASH. Cell Biosci. 2024;14(1):31. 10.1186/s13578-024-01215-7. 38461242 10.1186/s13578-024-01215-7 PMC10925010

[42] Paolini E, Longo M, Meroni M, Dongiovanni P. Hepatic zonation in MASLD: old question, new challenge in the era of spatial omics. Int J Mol Sci. 2025;26(21):10701. 10.3390/ijms262110701. 41226735 10.3390/ijms262110701 PMC12609896

[43] Chen G, Dai W, Wang J, Shen Z, Guo Y, Ge Q, et al. Endothelial RAP1A attenuates sinusoidal capillarisation and liver fibrosis by inhibiting RAF1-mediated Notch activation. Gut. 2026. 10.1136/gutjnl-2025-337143. 41672761 10.1136/gutjnl-2025-337143

[44] Fu SW, Gao YX, Li HY, Wu JC, Li ZH, Xu MY. The ameliorative effect of C-Kit+ hepatic endothelial cells with Mertk deficiency on nonalcoholic steatohepatitis. Biochim Biophys Acta Mol Basis Dis. 2025;1871(7):167964. 10.1016/j.bbadis.2025.167964. 40582627 10.1016/j.bbadis.2025.167964

[45] Kocabayoglu P, Lade A, Lee YA, Dragomir AC, Sun X, Fiel MI, et al. β-PDGF receptor expressed by hepatic stellate cells regulates fibrosis in murine liver injury, but not carcinogenesis. J Hepatol. 2015;63(1):141–7. 10.1016/j.jhep.2015.01.036. 25678385 10.1016/j.jhep.2015.01.036 PMC4475471

[46] Kim Y, Fiel MI, Albanis E, Chou HI, Zhang W, Khitrov G, et al. Anti-fibrotic activity and enhanced interleukin-6 production by hepatic stellate cells in response to imatinib mesylate. Liver Int. 2012;32(6):1008–17. 10.1111/j.1478-3231.2012.02806.x. 22507133 10.1111/j.1478-3231.2012.02806.x PMC3370152

[47] Li K, Kumar V, To T, Gonzalez-Dominguez A, Huria J, Yu M, et al. Scar-associated endothelial-stellate cellular crosstalk drives fibrosis resolution in MASH. Cell Rep. 2026;45(2):116915. 10.1016/j.celrep.2025.116915. 41581151 10.1016/j.celrep.2025.116915 PMC13010457

[48] Wilkinson AL, Hulme S, Kennedy JI, Mann ER, Horn P, Shepherd EL, et al. The senescent secretome drives PLVAP expression in cultured human hepatic endothelial cells to promote monocyte transmigration. iScience. 2023;26(10):107966. 10.1016/j.isci.2023.107966. 37810232 10.1016/j.isci.2023.107966 PMC10558774

[49] de Haan W, Oie C, Benkheil M, Dheedene W, Vinckier S, Coppiello G, et al. Unraveling the transcriptional determinants of liver sinusoidal endothelial cell specialization. Am J Physiol Gastrointest Liver Physiol. 2020;318(4):G803–15. 10.1152/ajpgi.00215.2019. 32116021 10.1152/ajpgi.00215.2019 PMC7191457

[50] Gracia-Sancho J, Russo L, Garcia-Caldero H, Garcia-Pagan JC, Garcia-Cardena G, Bosch J. Endothelial expression of transcription factor Kruppel-like factor 2 and its vasoprotective target genes in the normal and cirrhotic rat liver. Gut. 2011;60(4):517–24. 10.1136/gut.2010.220913. 21112949 10.1136/gut.2010.220913

[51] Gomez-Salinero JM, Izzo F, Lin Y, Houghton S, Itkin T, Geng F, et al. Specification of fetal liver endothelial progenitors to functional zonated adult sinusoids requires c-Maf induction. Cell Stem Cell. 2022;29(4):593-609.e7. 10.1016/j.stem.2022.03.002. 35364013 10.1016/j.stem.2022.03.002 PMC9290393

[52] Nagy D, Maude H, Birdsey GM, Randi AM, Cebola I. RISING STARS: liver sinusoidal endothelial transcription factors in metabolic homeostasis and disease. J Mol Endocrinol. 2023;71(2):e230026. 10.1530/JME-23-0026. 37306684 10.1530/JME-23-0026

[53] El Taghdouini A, Sorensen AL, Reiner AH, Coll M, Verhulst S, Mannaerts I, et al. Genome-wide analysis of DNA methylation and gene expression patterns in purified, uncultured human liver cells and activated hepatic stellate cells. Oncotarget. 2015;6(29):26729–45. 10.18632/oncotarget.4925. 26353929 10.18632/oncotarget.4925 PMC4694948

[54] Chen T, Shi Z, Zhao Y, Meng X, Zhao S, Zheng L, et al. LncRNA Airn maintains LSEC differentiation to alleviate liver fibrosis via the KLF2-eNOS-sGC pathway. BMC Med. 2022;20(1):335. 10.1186/s12916-022-02523-w. 36171606 10.1186/s12916-022-02523-w PMC9520944

[55] Abad-Jorda L, Martinez-Alcocer A, Guixe-Muntet S, Hunt NJ, Westwood LJ, Lozano JJ, et al. miR-27b-3p modulates liver sinusoidal endothelium dedifferentiation in chronic liver disease. Hepatol Commun. 2025;9(5):e0700. 10.1097/HC9.0000000000000700. 40304581 10.1097/HC9.0000000000000700 PMC12045533

[56] Yang L, Xu X, Chen Z, Zhang Y, Chen H, Wang X. MiR-511-3p promotes hepatic sinusoidal obstruction syndrome by activating hedgehog pathway via targeting Ptch1. Am J Physiol Gastrointest Liver Physiol. 2021;321(3):G344–54. 10.1152/ajpgi.00081.2021. 34287088 10.1152/ajpgi.00081.2021

[57] Selicean SE, Felli E, Wang C, Nulan Y, Lozano JJ, Guixe-Muntet S, et al. Stiffness-induced modulation of ERG transcription factor in chronic liver disease. NPJ Gut Liver. 2024;1(1):7. 10.1038/s44355-024-00007-7. 39381160 10.1038/s44355-024-00007-7 PMC11459910

[58] Dimmeler S, Fleming I, Fisslthaler B, Hermann C, Busse R, Zeiher AM. Activation of nitric oxide synthase in endothelial cells by Akt-dependent phosphorylation. Nature. 1999;399(6736):601–5. 10.1038/21224. 10376603 10.1038/21224

[59] Fulton D, Gratton JP, McCabe TJ, Fontana J, Fujio Y, Walsh K, et al. Regulation of endothelium-derived nitric oxide production by the protein kinase Akt. Nature. 1999;399(6736):597–601. 10.1038/21218. 10376602 10.1038/21218 PMC3637917

[60] David L, Mallet C, Keramidas M, Lamande N, Gasc JM, Dupuis-Girod S, et al. Bone morphogenetic protein-9 is a circulating vascular quiescence factor. Circ Res. 2008;102(8):914–22. 10.1161/CIRCRESAHA.107.165530. 18309101 10.1161/CIRCRESAHA.107.165530 PMC2561062

[61] Duan JL, Ruan B, Yan XC, Liang L, Song P, Yang ZY, et al. Endothelial Notch activation reshapes the angiocrine of sinusoidal endothelia to aggravate liver fibrosis and blunt regeneration in mice. Hepatology. 2018;68(2):677–90. 10.1002/hep.29834. 29420858 10.1002/hep.29834 PMC6099357

[62] Kaczara P, Czyzynska-Cichon I, Kus E, Kurpinska A, Olkowicz M, Wojnar-Lason K, et al. Liver sinusoidal endothelial cells rely on oxidative phosphorylation but avoid processing long-chain fatty acids in their mitochondria. Cell Mol Biol Lett. 2024;29(1):67. 10.1186/s11658-024-00584-8. 38724891 10.1186/s11658-024-00584-8 PMC11084093

[63] Xie G, Wang X, Wang L, Wang L, Atkinson RD, Kanel GC, et al. Role of differentiation of liver sinusoidal endothelial cells in progression and regression of hepatic fibrosis in rats. Gastroenterology. 2012;142(4):918-927.e6. 10.1053/j.gastro.2011.12.017. 22178212 10.1053/j.gastro.2011.12.017 PMC3618963

[64] Chen T, Zhang H, Shan W, Zhou J, You Y. Liver sinusoidal endothelial cells in hepatic fibrosis: opportunities for future strategies. Biochem Biophys Res Commun. 2025;766:151881. 10.1016/j.bbrc.2025.151881. 40286764 10.1016/j.bbrc.2025.151881

[65] Ito Y, Hosono K, Amano H. Responses of hepatic sinusoidal cells to liver ischemia-reperfusion injury. Front Cell Dev Biol. 2023;11:1171317. 10.3389/fcell.2023.1171317. 37082623 10.3389/fcell.2023.1171317 PMC10112669

[66] Biagioli M, Marchiano S, di Giorgio C, Roselli R, Bordoni M, Bellini R, et al. Combinatorial targeting of g-protein-coupled bile acid receptor 1 and cysteinyl leukotriene receptor 1 reveals a mechanistic role for bile acids and leukotrienes in drug-induced liver injury. Hepatology. 2023;78(1):26–44. 10.1002/hep.32787. 36107019 10.1002/hep.32787

[67] Nasiri-Ansari N, Androutsakos T, Flessa CM, Kyrou I, Siasos G, Randeva HS, et al. Endothelial cell dysfunction and nonalcoholic fatty liver disease (NAFLD): a concise review. Cells. 2022;11(16):2511. 10.3390/cells11162511. 36010588 10.3390/cells11162511 PMC9407007

[68] Yang Y, Sangwung P, Kondo R, Jung Y, McConnell MJ, Jeong J, et al. Alcohol-induced Hsp90 acetylation is a novel driver of liver sinusoidal endothelial dysfunction and alcohol-related liver disease. J Hepatol. 2021;75(2):377–86. 10.1016/j.jhep.2021.02.028. 33675874 10.1016/j.jhep.2021.02.028 PMC8292196

[69] Xie X, Luo J, Zhu D, Zhou W, Yang X, Feng X, et al. HBeAg is indispensable for inducing liver sinusoidal endothelial cell activation by hepatitis B virus. Front Cell Infect Microbiol. 2022;12:797915. 10.3389/fcimb.2022.797915. 35174107 10.3389/fcimb.2022.797915 PMC8842949

[70] Baiocchini A, Del Nonno F, Taibi C, Visco-Comandini U, D’Offizi G, Piacentini M, et al. Liver sinusoidal endothelial cells (LSECs) modifications in patients with chronic hepatitis C. Sci Rep. 2019;9(1):8760. 10.1038/s41598-019-45114-1. 31217430 10.1038/s41598-019-45114-1 PMC6584733

[71] Liu J, Man K. Mechanistic insight and clinical implications of Ischemia/Reperfusion injury post liver transplantation. Cell Mol Gastroenterol Hepatol. 2023;15(6):1463–74. 10.1016/j.jcmgh.2023.03.003. 36940849 10.1016/j.jcmgh.2023.03.003 PMC10160787

[72] Chen DQ, Guo Y, Li X, Zhang GQ, Li P. Small molecules as modulators of regulated cell death against ischemia/reperfusion injury. Med Res Rev. 2022;42(6):2067–101. 10.1002/med.21917. 35730121 10.1002/med.21917

[73] Huet PM, Nagaoka MR, Desbiens G, Tarrab E, Brault A, Bralet MP, et al. Sinusoidal endothelial cell and hepatocyte death following cold ischemia-warm reperfusion of the rat liver. Hepatology. 2004;39(4):1110–9. 10.1002/hep.20157. 15057915 10.1002/hep.20157

[74] Wang X, Walkey CJ, Maretti-Mira AC, Wang L, Johnson DL, DeLeve LD. Susceptibility of rat steatotic liver to ischemia-reperfusion is treatable with liver-selective matrix metalloproteinase inhibition. Hepatology. 2020;72(5):1771–85. 10.1002/hep.31179. 32060938 10.1002/hep.31179 PMC7523533

[75] Guixe-Muntet S, de Mesquita FC, Vila S, Hernandez-Gea V, Peralta C, Garcia-Pagan JC, et al. Cross-talk between autophagy and KLF2 determines endothelial cell phenotype and microvascular function in acute liver injury. J Hepatol. 2017;66(1):86–94. 10.1016/j.jhep.2016.07.051. 27545498 10.1016/j.jhep.2016.07.051

[76] Peralta C, Jimenez-Castro MB, Gracia-Sancho J. Hepatic ischemia and reperfusion injury: effects on the liver sinusoidal milieu. J Hepatol. 2013;59(5):1094–106. 10.1016/j.jhep.2013.06.017. 23811302 10.1016/j.jhep.2013.06.017

[77] Yoon Y, Gong SC, Kim MY, Baik SK, Hong JE, Rhee KJ, et al. Generation of fibrotic liver organoids using hepatocytes, primary liver sinusoidal endothelial cells, hepatic stellate cells, and macrophages. Cells. 2023;12(21):2514. 10.3390/cells12212514. 37947592 10.3390/cells12212514 PMC10647544

[78] Hilscher MB, Sehrawat T, Arab JP, Zeng Z, Gao J, Liu M, et al. Mechanical stretch increases expression of CXCL1 in liver sinusoidal endothelial cells to recruit neutrophils, generate sinusoidal microthombi, and promote portal hypertension. Gastroenterology. 2019;157(1):193-209.e9. 10.1053/j.gastro.2019.03.013. 30872106 10.1053/j.gastro.2019.03.013 PMC6581607

[79] Byrnes K, Blessinger S, Bailey NT, Scaife R, Liu G, Khambu B. Therapeutic regulation of autophagy in hepatic metabolism. Acta Pharm Sin B. 2022;12(1):33–49. 10.1016/j.apsb.2021.07.021. 35127371 10.1016/j.apsb.2021.07.021 PMC8799888

[80] Hide D, Warren A, Fernandez-Iglesias A, Maeso-Diaz R, Peralta C, Le Couteur DG, et al. Ischemia/Reperfusion injury in the aged liver: the importance of the sinusoidal endothelium in developing therapeutic strategies for the elderly. J Gerontol A Biol Sci Med Sci. 2020;75(2):268–77. 10.1093/gerona/glz012. 30649272 10.1093/gerona/glz012

[81] Qu S, Yuan B, Zhang H, Huang H, Zeng Z, Yang S, et al. Heme oxygenase 1 attenuates hypoxia-reoxygenation injury in mice liver sinusoidal endothelial cells. Transplantation. 2018;102(3):426–32. 10.1097/TP.0000000000002028. 29189483 10.1097/TP.0000000000002028

[82] Gage BK, Liu JC, Innes BT, MacParland SA, McGilvray ID, Bader GD, et al. Generation of functional liver sinusoidal endothelial cells from human pluripotent stem-cell-derived venous angioblasts. Cell Stem Cell. 2020;27(2):254-269.e9. 10.1016/j.stem.2020.06.007. 32640183 10.1016/j.stem.2020.06.007

[83] Batkai S, Osei-Hyiaman D, Pan H, El-Assal O, Rajesh M, Mukhopadhyay P, et al. Cannabinoid-2 receptor mediates protection against hepatic ischemia/reperfusion injury. FASEB J. 2007;21(8):1788–800. 10.1096/fj.06-7451com. 17327359 10.1096/fj.06-7451com PMC2228252

[84] Jimenez-Castro MB, Casillas-Ramirez A, Negrete-Sanchez E, Avalos-de Leon CG, Gracia-Sancho J, Peralta C. Adipocytokines in steatotic liver Surgery/Transplantation. Transplantation. 2019;103(1):71–7. 10.1097/TP.0000000000002098. 30586349 10.1097/TP.0000000000002098

[85] Hong F, Radaeva S, Pan HN, Tian Z, Veech R, Gao B. Interleukin 6 alleviates hepatic steatosis and ischemia/reperfusion injury in mice with fatty liver disease. Hepatology. 2004;40(4):933–41. 10.1002/hep.20400. 15382116 10.1002/hep.20400

[86] Guo Q, Furuta K, Lucien F, Gutierrez Sanchez LH, Hirsova P, Krishnan A, et al. Integrin beta(1)-enriched extracellular vesicles mediate monocyte adhesion and promote liver inflammation in murine NASH. J Hepatol. 2019;71(6):1193–205. 10.1016/j.jhep.2019.07.019. 31433301 10.1016/j.jhep.2019.07.019 PMC6864271

[87] Wallace SJ, Tacke F, Schwabe RF, Henderson NC. Understanding the cellular interactome of non-alcoholic fatty liver disease. JHEP Rep. 2022;4(8):100524. 10.1016/j.jhepr.2022.100524. 35845296 10.1016/j.jhepr.2022.100524 PMC9284456

[88] Ojha U, Kim S, Rhee CY, You J, Choi YH, Yoon SH, et al. Endothelial RUNX3 controls LSEC dysfunction and angiocrine LRG1 signaling to prevent liver fibrosis. Hepatology. 2025;81(4):1228–43. 10.1097/HEP.0000000000001018. 39042837 10.1097/HEP.0000000000001018 PMC11902585

[89] Cooper SA, Kostallari E, Shah VH. Angiocrine signaling in sinusoidal health and disease. Semin Liver Dis. 2023;43(3):245–57. 10.1055/a-2128-5907. 37442155 10.1055/a-2128-5907 PMC10798369

[90] Minamino T, Ito Y, Ohkubo H, Hosono K, Suzuki T, Sato T, et al. Thromboxane A(2) receptor signaling promotes liver tissue repair after toxic injury through the enhancement of macrophage recruitment. Toxicol Appl Pharmacol. 2012;259(1):104–14. 10.1016/j.taap.2011.12.013. 22206755 10.1016/j.taap.2011.12.013

[91] Mohamed YS, Abdelsalam RM, Attia AS, Abdel-Aziz MT, El-Tanbouly DM. Regulation of liver regeneration by prostaglandin E(2) and thromboxane A(2) following partial hepatectomy in rats. Naunyn Schmiedebergs Arch Pharmacol. 2020;393(8):1437–46. 10.1007/s00210-020-01848-8. 32162076 10.1007/s00210-020-01848-8

[92] Allison R, Guraka A, Shawa IT, Tripathi G, Moritz W, Kermanizadeh A. Drug induced liver injury - a 2023 update. J Toxicol Environ Health B Crit Rev. 2023;26(8):442–67. 10.1080/10937404.2023.2261848. 37786264 10.1080/10937404.2023.2261848

[93] Fontana RJ, Liou I, Reuben A, Suzuki A, Fiel MI, Lee W, et al. AASLD practice guidance on drug, herbal, and dietary supplement-induced liver injury. Hepatology. 2023;77(3):1036–65. 10.1002/hep.32689. 35899384 10.1002/hep.32689 PMC9936988

[94] Yan M, Huo Y, Yin S, Hu H. Mechanisms of acetaminophen-induced liver injury and its implications for therapeutic interventions. Redox Biol. 2018;17:274–83. 10.1016/j.redox.2018.04.019. 29753208 10.1016/j.redox.2018.04.019 PMC6006912

[95] Bessems JG, Vermeulen NP. Paracetamol (acetaminophen)-induced toxicity: molecular and biochemical mechanisms, analogues and protective approaches. Crit Rev Toxicol. 2001;31(1):55–138. 10.1080/20014091111677. 11215692 10.1080/20014091111677

[96] Luo G, Huang L, Zhang Z. The molecular mechanisms of acetaminophen-induced hepatotoxicity and its potential therapeutic targets. Exp Biol Med (Maywood). 2023;248(5):412–24. 10.1177/15353702221147563. 36670547 10.1177/15353702221147563 PMC10281617

[97] DeLeve LD. Hepatic microvasculature in liver injury. Semin Liver Dis. 2007;27(4):390–400. 10.1055/s-2007-991515. 17979075 10.1055/s-2007-991515

[98] Bjornsson ES. Drug-induced liver injury: an overview over the most critical compounds. Arch Toxicol. 2015;89(3):327–34. 10.1007/s00204-015-1456-2. 25618544 10.1007/s00204-015-1456-2

[99] Hessel-Pras S, Braeuning A, Guenther G, Adawy A, Enge AM, Ebmeyer J, et al. The pyrrolizidine alkaloid senecionine induces CYP-dependent destruction of sinusoidal endothelial cells and cholestasis in mice. Arch Toxicol. 2020;94(1):219–29. 10.1007/s00204-019-02582-8. 31606820 10.1007/s00204-019-02582-8

[100] Zuckerman M, Steenkamp V, Stewart MJ. Hepatic veno-occlusive disease as a result of a traditional remedy: confirmation of toxic pyrrolizidine alkaloids as the cause, using an in vitro technique. J Clin Pathol. 2002;55(9):676–9. 10.1136/jcp.55.9.676. 12194997 10.1136/jcp.55.9.676 PMC1769740

[101] Lawson JA, Fisher MA, Simmons CA, Farhood A, Jaeschke H. Parenchymal cell apoptosis as a signal for sinusoidal sequestration and transendothelial migration of neutrophils in murine models of endotoxin and Fas-antibody-induced liver injury. Hepatology. 1998;28(3):761–7. 10.1002/hep.510280324. 9731570 10.1002/hep.510280324

[102] Chosay JG, Essani NA, Dunn CJ, Jaeschke H. Neutrophil margination and extravasation in sinusoids and venules of liver during endotoxin-induced injury. Am J Physiol. 1997;272(5 Pt 1):G1195–200. 10.1152/ajpgi.1997.272.5.G1195. 9176230 10.1152/ajpgi.1997.272.5.G1195

[103] Yan M, Man S, Ma L, Guo L, Huang L, Gao W. Immunological mechanisms in steatotic liver diseases: an overview and clinical perspectives. Clin Mol Hepatol. 2024;30(4):620–48. 10.3350/cmh.2024.0315. 38988278 10.3350/cmh.2024.0315 PMC11540396

[104] Rinella ME, Lazarus JV, Ratziu V, Francque SM, Sanyal AJ, Kanwal F, et al. A multi-society Delphi consensus statement on new fatty liver disease nomenclature. J Hepatol. 2023;79(6):1542–56. 10.1016/j.jhep.2023.06.003. 37364790 10.1016/j.jhep.2023.06.003

[105] Iwakiri Y, Trebicka J. Portal hypertension in cirrhosis: pathophysiological mechanisms and therapy. JHEP Rep. 2021;3(4):100316. 10.1016/j.jhepr.2021.100316. 34337369 10.1016/j.jhepr.2021.100316 PMC8318926

[106] Hwang JH, Heo W, Park JI, Kim KM, Oh HT, Yoo GD, et al. Endothelial TAZ inhibits capillarization of liver sinusoidal endothelium and damage-induced liver fibrosis via nitric oxide production. Theranostics. 2023;13(12):4182–96. 10.7150/thno.83714. 37554269 10.7150/thno.83714 PMC10405847

[107] Wang J, Guo Y, Hu J, Peng J. STING activation in various cell types in metabolic dysfunction-associated steatotic liver disease. Liver Int. 2025;45(4):e70063. 10.1111/liv.70063. 40116753 10.1111/liv.70063

[108] Abumrad NA, Cabodevilla AG, Samovski D, Pietka T, Basu D, Goldberg IJ. Endothelial cell receptors in tissue lipid uptake and metabolism. Circ Res. 2021;128(3):433–50. 10.1161/CIRCRESAHA.120.318003. 33539224 10.1161/CIRCRESAHA.120.318003 PMC7959116

[109] Simon J, Nunez-Garcia M, Fernandez-Tussy P, Barbier-Torres L, Fernandez-Ramos D, Gomez-Santos B, et al. Targeting hepatic glutaminase 1 ameliorates non-alcoholic steatohepatitis by restoring very-low-density lipoprotein triglyceride assembly. Cell Metab. 2020;31(3):605-622.e10. 10.1016/j.cmet.2020.01.013. 32084378 10.1016/j.cmet.2020.01.013 PMC7259377

[110] Guo M, Li M, Chen L, Wang H, Wang J, Niu P, et al. Glutaminase 1 isoform up-regulation associated with lipid metabolism disorder induced by methyl tertiary-butyl ether in male rats. Ecotoxicol Environ Saf. 2023;255:114763. 10.1016/j.ecoenv.2023.114763. 37032576 10.1016/j.ecoenv.2023.114763

[111] Li Z, Luo G, Gan C, Zhang H, Li L, Zhang X, et al. Spatially resolved multi-omics of human metabolic dysfunction-associated steatotic liver disease. Nat Genet. 2025;57(12):3112–25. 10.1038/s41588-025-02407-8. 41286103 10.1038/s41588-025-02407-8 PMC12695644

[112] Rautou PE, Chotkoe S, Biquard L, Wettstein G, van der Graaff D, Liu Y, et al. Altered liver sinusoidal endothelial cells in MASLD and their evolution following lanifibranor treatment. JHEP Rep. 2025;7(6):101366. 10.1016/j.jhepr.2025.101366. 40486133 10.1016/j.jhepr.2025.101366 PMC12142333

[113] Israelsen M, Torp N, Johansen S, Thiele M, Krag A. MetALD: new opportunities to understand the role of alcohol in steatotic liver disease. Lancet Gastroenterol Hepatol. 2023;8(10):866–8. 10.1016/S2468-1253(23)00206-6. 37567208 10.1016/S2468-1253(23)00206-6

[114] Jung E, Baek EB, Hong EJ, Kang JH, Park S, Park S, et al. TXNIP in liver sinusoidal endothelial cells ameliorates alcohol-associated liver disease via nitric oxide production. Int J Biol Sci. 2024;20(2):606–20. 10.7150/ijbs.90781. 38169654 10.7150/ijbs.90781 PMC10758096

[115] Makki K, Froguel P, Wolowczuk I. Adipose tissue in obesity-related inflammation and insulin resistance: cells, cytokines, and chemokines. ISRN Inflamm. 2013;2013:139239. 10.1155/2013/139239. 24455420 10.1155/2013/139239 PMC3881510

[116] Jimenez-Cortegana C, Garcia-Galey A, Tami M, Del Pino P, Carmona I, Lopez S, et al. Role of leptin in non-alcoholic fatty liver disease. Biomedicines. 2021;9(7):762. 10.3390/biomedicines9070762. 34209386 10.3390/biomedicines9070762 PMC8301314

[117] Eguchi A, Lazic M, Armando AM, Phillips SA, Katebian R, Maraka S, et al. Circulating adipocyte-derived extracellular vesicles are novel markers of metabolic stress. J Mol Med (Berl). 2016;94(11):1241–53. 10.1007/s00109-016-1446-8. 27394413 10.1007/s00109-016-1446-8 PMC5071132

[118] Babuta M, Szabo G. Extracellular vesicles in inflammation: focus on the microRNA cargo of EVs in modulation of liver diseases. J Leukoc Biol. 2022;111(1):75–92. 10.1002/JLB.3MIR0321-156R. 34755380 10.1002/JLB.3MIR0321-156R PMC9235439

[119] Geraud C, Koch PS, Zierow J, Klapproth K, Busch K, Olsavszky V, et al. GATA4-dependent organ-specific endothelial differentiation controls liver development and embryonic hematopoiesis. J Clin Invest. 2017;127(3):1099–114. 10.1172/JCI90086. 28218627 10.1172/JCI90086 PMC5330741

[120] Young A, Wu W, Sun W, Benjamin Larman H, Wang N, Li YS, et al. Flow activation of AMP-activated protein kinase in vascular endothelium leads to Kruppel-like factor 2 expression. Arterioscler Thromb Vasc Biol. 2009;29(11):1902–8. 10.1161/ATVBAHA.109.193540. 19696400 10.1161/ATVBAHA.109.193540 PMC2766008

[121] DeLeve LD. Liver sinusoidal endothelial cells in hepatic fibrosis. Hepatology. 2015;61(5):1740–6. 10.1002/hep.27376. 25131509 10.1002/hep.27376 PMC4333127

[122] Clark SA, Angus HB, Cook HB, George PM, Oxner RB, Fraser R. Defenestration of hepatic sinusoids as a cause of hyperlipoproteinaemia in alcoholics. Lancet. 1988;2(8622):1225–7. 10.1016/s0140-6736(88)90813-6. 2903956 10.1016/s0140-6736(88)90813-6

[123] He S, Luo Y, Ma W, Wang X, Yan C, Hao W, et al. Endothelial POFUT1 controls injury-induced liver fibrosis by repressing fibrinogen synthesis. J Hepatol. 2024;81(1):135–48. 10.1016/j.jhep.2024.02.032. 38460791 10.1016/j.jhep.2024.02.032

[124] Guixe-Muntet S, Ortega-Ribera M, Wang C, Selicean S, Andreu I, Kechagia JZ, et al. Nuclear deformation mediates liver cell mechanosensing in cirrhosis. JHEP Rep. 2020;2(5):100145. 10.1016/j.jhepr.2020.100145. 32939447 10.1016/j.jhepr.2020.100145 PMC7479345

[125] Asada S, Kaji K, Nishimura N, Koizumi A, Matsuda T, Tanaka M, et al. Tofogliflozin delays portal hypertension and hepatic fibrosis by inhibiting sinusoidal capillarization in cirrhotic rats. Cells. 2024;13(6):538. 10.3390/cells13060538. 38534382 10.3390/cells13060538 PMC10968969

[126] Rensing H, Bauer I, Zhang JX, Paxian M, Pannen B, Yokoyama Y, et al. Endothelin-1 and heme oxygenase-1 as modulators of sinusoidal tone in the stress-exposed rat liver. Hepatology. 2002;36(6):1453–65. 10.1053/jhep.2002.36934. 12447872 10.1053/jhep.2002.36934

[127] Graupera M, Garcia-Pagan JC, Pares M, Abraldes JG, Rosello J, Bosch J, et al. Cyclooxygenase-1 inhibition corrects endothelial dysfunction in cirrhotic rat livers. J Hepatol. 2003;39(4):515–21. 10.1016/s0168-8278(03)00347-7. 12971960 10.1016/s0168-8278(03)00347-7

[128] Steib CJ, Bilzer M, op den Winkel M, Pfeiler S, Hartmann AC, Hennenberg M, et al. Treatment with the leukotriene inhibitor montelukast for 10 days attenuates portal hypertension in rat liver cirrhosis. Hepatology. 2010;51(6):2086–96. 10.1002/hep.23596. 20512996 10.1002/hep.23596

[129] Rosado E, Rodriguez-Vilarrupla A, Gracia-Sancho J, Monclus M, Bosch J, Garcia-Pagan JC. Interaction between NO and COX pathways modulating hepatic endothelial cells from control and cirrhotic rats. J Cell Mol Med. 2012;16(10):2461–70. 10.1111/j.1582-4934.2012.01563.x. 22436078 10.1111/j.1582-4934.2012.01563.x PMC3823440

[130] Liu S, Luttrell LM, Premont RT, Rockey DC. beta-Arrestin2 is a critical component of the GPCR-eNOS signalosome. Proc Natl Acad Sci U S A. 2020;117(21):11483–92. 10.1073/pnas.1922608117. 32404425 10.1073/pnas.1922608117 PMC7261012

[131] Bosch M, Kallin N, Donakonda S, Zhang JD, Wintersteller H, Hegenbarth S, et al. A liver immune rheostat regulates CD8 T cell immunity in chronic HBV infection. Nature. 2024;631(8022):867–75. 10.1038/s41586-024-07630-7. 38987588 10.1038/s41586-024-07630-7 PMC11269190

[132] Jeng WJ, Papatheodoridis GV, Lok ASF. Hepatitis B. Lancet. 2023;401(10381):1039–52. 10.1016/S0140-6736(22)01468-4. 36774930 10.1016/S0140-6736(22)01468-4

[133] Lemon SM, Ott JJ, Van Damme P, Shouval D. Type A viral hepatitis: A summary and update on the molecular virology, epidemiology, pathogenesis and prevention. J Hepatol. 2018;68(1):167–84. 10.1016/j.jhep.2017.08.034. 10.1016/j.jhep.2017.08.034 28887164

[134] Kamar N, Izopet J, Pavio N, Aggarwal R, Labrique A, Wedemeyer H, et al. Hepatitis E virus infection. Nat Rev Dis Primers. 2017;3:17086. 10.1038/nrdp.2017.86. 29154369 10.1038/nrdp.2017.86

[135] Do A, Reau NS. Chronic viral hepatitis: Current management and future directions. Hepatol Commun. 2020;4(3):329–41. 10.1002/hep4.1480. 32140652 10.1002/hep4.1480 PMC7049676

[136] Polaris Observatory Collaborators. Global prevalence, cascade of care, and prophylaxis coverage of hepatitis B in 2022: A modelling study. Lancet Gastroenterol Hepatol. 2023;8(10):879–907. 10.1016/S2468-1253(23)00197-8. 37517414 10.1016/S2468-1253(23)00197-8

[137] de Martel C, Georges D, Bray F, Ferlay J, Clifford GM. Global burden of cancer attributable to infections in 2018: A worldwide incidence analysis. Lancet Glob Health. 2020;8(2):e180–90. 10.1016/S2214-109X(19)30488-7. 31862245 10.1016/S2214-109X(19)30488-7

[138] Wu J, Meng Z, Jiang M, Pei R, Trippler M, Broering R, et al. Hepatitis B virus suppresses Toll-like receptor-mediated innate immune responses in murine parenchymal and nonparenchymal liver cells. Hepatology. 2009;49(4):1132–40. 10.1002/hep.22751. 19140219 10.1002/hep.22751

[139] Zhang E, Lu M. Toll-like receptor (TLR)-mediated innate immune responses in the control of hepatitis B virus (HBV) infection. Med Microbiol Immunol. 2015;204(1):11–20. 10.1007/s00430-014-0370-1. 25550115 10.1007/s00430-014-0370-1 PMC4305100

[140] Du Y, Wu J, Liu J, Zheng X, Yang D, Lu M. Toll-like receptor-mediated innate immunity orchestrates adaptive immune responses in HBV infection. Front Immunol. 2022;13:965018. 10.3389/fimmu.2022.965018. 35967443 10.3389/fimmu.2022.965018 PMC9372436

[141] Yang S, Wang L, Pan W, Bayer W, Thoens C, Heim K, et al. MMP2/MMP9-mediated CD100 shedding is crucial for inducing intrahepatic anti-HBV CD8 T cell responses and HBV clearance. J Hepatol. 2019;71(4):685–98. 10.1016/j.jhep.2019.05.013. 31173811 10.1016/j.jhep.2019.05.013

[142] Giugliano S, Kriss M, Golden-Mason L, Dobrinskikh E, Stone AE, Soto-Gutierrez A, et al. Hepatitis C virus infection induces autocrine interferon signaling by human liver endothelial cells and release of exosomes, which inhibits viral replication. Gastroenterology. 2015;148(2):392-402.e13. 10.1053/j.gastro.2014.10.040. 25447848 10.1053/j.gastro.2014.10.040 PMC4765499

[143] Rowe IA, Galsinh SK, Wilson GK, Parker R, Durant S, Lazar C, et al. Paracrine signals from liver sinusoidal endothelium regulate hepatitis C virus replication. Hepatology. 2014;59(2):375–84. 10.1002/hep.26571. 23775568 10.1002/hep.26571 PMC3992845

[144] Broering R, Wu J, Meng Z, Hilgard P, Lu M, Trippler M, et al. Toll-like receptor-stimulated non-parenchymal liver cells can regulate hepatitis C virus replication. J Hepatol. 2008;48(6):914–22. 10.1016/j.jhep.2008.01.028. 18362039 10.1016/j.jhep.2008.01.028

[145] Schmidt FP, Zimmermann T, Wenz T, Schnorbus B, Ostad MA, Feist C, et al. Interferon- and ribavirin-free therapy with new direct acting antivirals (DAA) for chronic hepatitis C improves vascular endothelial function. Int J Cardiol. 2018;271:296–300. 10.1016/j.ijcard.2018.04.058. 30077529 10.1016/j.ijcard.2018.04.058

[146] Caballero-Marcos A, Romero-Cristobal M, Puerto M, Fernandez-Yunquera A, Dieguez L, Navarrete C, et al. HCV eradication in recurrent hepatitis C after liver transplantation normalizes enhanced endothelial activation. Transpl Int. 2021;34(11):2214–25. 10.1111/tri.14000. 34346111 10.1111/tri.14000

[147] Lu Y, Liu Y, Zuo X, Li G, Wang J, Liu J, et al. CXCL12+ tumor-associated endothelial cells promote immune resistance in hepatocellular carcinoma. J Hepatol. 2025;82(4):634–48. 10.1016/j.jhep.2024.09.044. 39393439 10.1016/j.jhep.2024.09.044

[148] Sankar K, Gong J, Osipov A, Miles SA, Kosari K, Nissen NN, et al. Recent advances in the management of hepatocellular carcinoma. Clin Mol Hepatol. 2024;30(1):1–15. 10.3350/cmh.2023.0125. 37482076 10.3350/cmh.2023.0125 PMC10776289

[149] Matoba D, Noda T, Kobayashi S, Sakano Y, Matsumoto K, Yamanaka C, et al. Tumor endothelial cells induce M2 macrophage polarization via IL-4, enhancing tumor growth in hepatocellular carcinoma. Cancer Sci. 2025;116(11):2972–85. 10.1111/cas.70179. 40859796 10.1111/cas.70179 PMC12580878

[150] Xu F, Jin T, Zhu Y, Dai C. Immune checkpoint therapy in liver cancer. J Exp Clin Cancer Res. 2018;37(1):110. 10.1186/s13046-018-0777-4. 29843754 10.1186/s13046-018-0777-4 PMC5975687

[151] Langhans B, Nischalke HD, Kramer B, Dold L, Lutz P, Mohr R, et al. Role of regulatory T cells and checkpoint inhibition in hepatocellular carcinoma. Cancer Immunol Immunother. 2019;68(12):2055–66. 10.1007/s00262-019-02427-4. 31724091 10.1007/s00262-019-02427-4 PMC11028391

[152] Wadkin JCR, Patten DA, Kamarajah SK, Shepherd EL, Novitskaya V, Berditchevski F, et al. CD151 supports VCAM-1-mediated lymphocyte adhesion to liver endothelium and is upregulated in chronic liver disease and hepatocellular carcinoma. Am J Physiol Gastrointest Liver Physiol. 2017;313(2):G138–49. 10.1152/ajpgi.00411.2016. 28473332 10.1152/ajpgi.00411.2016 PMC5582880

[153] Zhang K, Zhu Z, Jia R, Wang NA, Shi M, Wang Y, et al. CD151-enriched migrasomes mediate hepatocellular carcinoma invasion by conditioning cancer cells and promoting angiogenesis. J Exp Clin Cancer Res. 2024;43(1):160. 10.1186/s13046-024-03082-z. 38840183 10.1186/s13046-024-03082-z PMC11155183

[154] Oura K, Morishita A, Tadokoro T, Fujita K, Tani J, Kobara H. Immune microenvironment and the effect of vascular endothelial growth factor inhibition in hepatocellular carcinoma. Int J Mol Sci. 2024;25(24):13590. 10.3390/ijms252413590. 39769351 10.3390/ijms252413590 PMC11679663

[155] Wu S, Anand N, Guo Z, Li M, Santiago Figueroa M, Jung L, et al. Bridging immune evasion and vascular dynamics for novel therapeutic frontiers in hepatocellular carcinoma. Cancers (Basel). 2025;17(11):1860. 10.3390/cancers17111860. 40507341 10.3390/cancers17111860 PMC12153674

[156] Wang XX, Jin S, Chao X, Liu Y, Cheng YH, Ji H, et al. Integrative single-cell and spatial transcriptomic reveals S100A16(+) tumor endothelial cells drive angiogenesis and immunosuppression in hepatocellular carcinoma. Cancer Lett. 2026;645:218335. 10.1016/j.canlet.2026.218335. 41722834 10.1016/j.canlet.2026.218335

[157] Benedicto A, Marquez J, Herrero A, Olaso E, Kolaczkowska E, Arteta B. Decreased expression of the beta(2) integrin on tumor cells is associated with a reduction in liver metastasis of colorectal cancer in mice. BMC Cancer. 2017;17(1):827. 10.1186/s12885-017-3823-2. 29207960 10.1186/s12885-017-3823-2 PMC5718006

[158] Benedicto A, Herrero A, Romayor I, Marquez J, Smedsrod B, Olaso E, et al. Liver sinusoidal endothelial cell ICAM-1 mediated tumor/endothelial crosstalk drives the development of liver metastasis by initiating inflammatory and angiogenic responses. Sci Rep. 2019;9(1):13111. 10.1038/s41598-019-49473-7. 31511625 10.1038/s41598-019-49473-7 PMC6739321

[159] Yu X, Chen L, Liu J, Dai B, Xu G, Shen G, et al. Immune modulation of liver sinusoidal endothelial cells by melittin nanoparticles suppresses liver metastasis. Nat Commun. 2019;10(1):574. 10.1038/s41467-019-08538-x. 30718511 10.1038/s41467-019-08538-x PMC6361944

[160] Benedicto A, Herrero A, Lopategi A, Friedman SL, Boyano MD, Arteta B. Depletion of tumor-reactive HSCs reveals their significance during different stages of liver metastasis. Hepatol Commun. 2025;9(4):e0669. 10.1097/HC9.0000000000000669. 40130990 10.1097/HC9.0000000000000669 PMC11936575

[161] Sun J, Zhang S, Liu Y, Liu K, Gu X. Exploring tumor endothelial cells heterogeneity in hepatocellular carcinoma: Insights from single-cell sequencing and pseudotime analysis. PeerJ. 2024;12:e18362. 10.7717/peerj.18362. 39484208 10.7717/peerj.18362 PMC11526786

[162] Shen J, Jiang F, Yang Y, Huang G, Pu F, Liu Q, et al. 14–3-3eta is a novel growth-promoting and angiogenic factor in hepatocellular carcinoma. J Hepatol. 2016;65(5):953–62. 10.1016/j.jhep.2016.05.017. 27210426 10.1016/j.jhep.2016.05.017

[163] Qiu Y, Dai Y, Zhang C, Yang Y, Jin M, Shan W, et al. Arsenic trioxide reverses the chemoresistance in hepatocellular carcinoma: a targeted intervention of 14–3-3eta/NF-kappaB feedback loop. J Exp Clin Cancer Res. 2018;37(1):321. 10.1186/s13046-018-1005-y. 30572915 10.1186/s13046-018-1005-y PMC6302299

[164] Jin M, Du X, Dong N, Feng Z, Sun Y, Song S, et al. A cigarette compound-induced tumor microenvironment promotes sorafenib resistance in hepatocellular carcinoma via the 14–3-3eta-modified tumor-associated proteome. Chin Med J (Engl). 2025. 10.1097/CM9.0000000000003851. 41292064 10.1097/CM9.0000000000003851 PMC13441109

[165] Zhang W, Hong X, Xiao Y, Wang H, Zeng X. Sorafenib resistance and therapeutic strategies in hepatocellular carcinoma. Biochim Biophys Acta Rev Cancer. 2025;1880(3):189310. 10.1016/j.bbcan.2025.189310. 40187502 10.1016/j.bbcan.2025.189310

[166] Qin Y, Han S, Yu Y, Qi D, Ran M, Yang M, et al. Lenvatinib in hepatocellular carcinoma: resistance mechanisms and strategies for improved efficacy. Liver Int. 2024;44(8):1808–31. 10.1111/liv.15953. 38700443 10.1111/liv.15953

[167] Ladd AD, Duarte S, Sahin I, Zarrinpar A. Mechanisms of drug resistance in HCC. Hepatology. 2024;79(4):926–40. 10.1097/HEP.0000000000000237. 36680397 10.1097/HEP.0000000000000237

[168] Carambia A, Freund B, Schwinge D, Bruns OT, Salmen SC, Ittrich H, et al. Nanoparticle-based autoantigen delivery to treg-inducing liver sinusoidal endothelial cells enables control of autoimmunity in mice. J Hepatol. 2015;62(6):1349–56. 10.1016/j.jhep.2015.01.006. 25617499 10.1016/j.jhep.2015.01.006

[169] Hide D, Ortega-Ribera M, Fernandez-Iglesias A, Fondevila C, Salvado MJ, Arola L, et al. A novel form of the human manganese superoxide dismutase protects rat and human livers undergoing ischaemia and reperfusion injury. Clin Sci (Lond). 2014;127(8):527–37. 10.1042/CS20140125. 24754522 10.1042/CS20140125

[170] Ito T, Kuriyama N, Kato H, Matsuda A, Mizuno S, Usui M, et al. Sinusoidal protection by sphingosine-1-phosphate receptor 1 agonist in liver ischemia-reperfusion injury. J Surg Res. 2018;222:139–52. 10.1016/j.jss.2017.09.048. 29273365 10.1016/j.jss.2017.09.048

[171] Yadav N, Jaber FL, Sharma Y, Gupta P, Viswanathan P, Gupta S. Efficient reconstitution of hepatic microvasculature by endothelin receptor antagonism in liver sinusoidal endothelial cells. Hum Gene Ther. 2019;30(3):365–77. 10.1089/hum.2018.166. 30266073 10.1089/hum.2018.166 PMC6437629

[172] Liu Z, Lai CH, Zhang X, Luo J, Huang X, Qi X, et al. Simvastatin ameliorates total liver ischemia/reperfusion injury via KLF2-mediated mechanism in rats. Clin Res Hepatol Gastroenterol. 2019;43(2):171–8. 10.1016/j.clinre.2018.08.014. 30274910 10.1016/j.clinre.2018.08.014

[173] Zhou W, Zhang Y, Hosch MS, Lang A, Zwacka RM, Engelhardt JF. Subcellular site of superoxide dismutase expression differentially controls AP-1 activity and injury in mouse liver following ischemia/reperfusion. Hepatology. 2001;33(4):902–14. 10.1053/jhep.2001.23073. 11283855 10.1053/jhep.2001.23073

[174] Mandili G, Alchera E, Merlin S, Imarisio C, Chandrashekar BR, Riganti C, et al. Mouse hepatocytes and LSEC proteome reveal novel mechanisms of ischemia/reperfusion damage and protection by A2aR stimulation. J Hepatol. 2015;62(3):573–80. 10.1016/j.jhep.2014.10.007. 25315650 10.1016/j.jhep.2014.10.007

[175] Ajamieh H, Farrell GC, McCuskey RS, Yu J, Chu E, Wong HJ, et al. Acute atorvastatin is hepatoprotective against ischaemia-reperfusion injury in mice by modulating eNOS and microparticle formation. Liver Int. 2015;35(9):2174–86. 10.1111/liv.12827. 25786512 10.1111/liv.12827

[176] Ricciardi R, Schaffer BK, Shah SA, Quarfordt SH, Banner BF, Wheeler SM, et al. Bosentan, an endothelin antagonist, augments hepatic graft function by reducing graft circulatory impairment following ischemia/reperfusion injury. J Gastrointest Surg. 2001;5(3):322–9. 10.1016/s1091-255x(01)80055-x. 11360057 10.1016/s1091-255x(01)80055-x

[177] Tiriveedhi V, Upadhya GA, Busch RA, Gunter KL, Dines JN, Knolhoff BL, et al. Protective role of bortezomib in steatotic liver ischemia/reperfusion injury through abrogation of MMP activation and YKL-40 expression. Transpl Immunol. 2014;30(2–3):93–8. 10.1016/j.trim.2013.12.003. 24380732 10.1016/j.trim.2013.12.003 PMC9814016

[178] Rabie MA, Zaki HF, Sayed HM. Telluric acid ameliorates hepatic ischemia reperfusion-induced injury in rats: involvement of TLR4, Nrf2, and PI3K/Akt signaling pathways. Biochem Pharmacol. 2019;168:404–11. 10.1016/j.bcp.2019.08.001. 31386827 10.1016/j.bcp.2019.08.001

[179] Sabry MM, Ramadan NM, Al Dreny BA, Rashed LA, Abo El Enein A. Protective effect of apelin preconditioning in a rat model of hepatic ischemia reperfusion injury; possible interaction between the apelin/APJ system, Ang II/AT1R system and eNOS. United Eur Gastroenterol J. 2019;7(5):689–98. 10.1177/2050640619826847. 10.1177/2050640619826847 PMC6545709 31210947

[180] Lassailly G, Bou Saleh M, Leleu-Chavain N, Ningarhari M, Gantier E, Carpentier R, et al. Nucleotide-binding oligomerization domain 1 (NOD1) modulates liver ischemia reperfusion through the expression adhesion molecules. J Hepatol. 2019;70(6):1159–69. 10.1016/j.jhep.2019.01.019. 30685324 10.1016/j.jhep.2019.01.019

[181] Liu J, Li C, Waalkes MP, Clark J, Myers P, Saavedra JE, et al. The nitric oxide donor, V-PYRRO/NO, protects against acetaminophen-induced hepatotoxicity in mice. Hepatology. 2003;37(2):324–33. 10.1053/jhep.2003.50063. 12540782 10.1053/jhep.2003.50063

[182] Wang X, Maretti-Mira AC, Wang L, DeLeve LD. Liver-selective MMP-9 inhibition in the rat eliminates ischemia-reperfusion injury and accelerates liver regeneration. Hepatology. 2019;69(1):314–28. 10.1002/hep.30169. 30019419 10.1002/hep.30169 PMC6325019

[183] Khajepour F, Mahmoodpoor F, Jafari E, Kakaei F, Bahraminia F, Aghajani S, et al. Prazosin protects the liver against renal Ischemia/Reperfusion injury in rats. Drug Res (Stuttg). 2023;73(5):289–95. 10.1055/a-2015-7976. 36940722 10.1055/a-2015-7976

[184] Yin H, Cheng L, Holt M, Hail N Jr, Maclaren R, Ju C. Lactoferrin protects against acetaminophen-induced liver injury in mice. Hepatology. 2010;51(3):1007–16. 10.1002/hep.23476. 20099297 10.1002/hep.23476 PMC2908515

[185] Guan S, Zhang S, Liu M, Guo J, Chen Y, Shen X, et al. Preventive effects of lactoferrin on acute alcohol-induced liver injury via iron chelation and regulation of iron metabolism. J Dairy Sci. 2024;107(8):5316–29. 10.3168/jds.2023-24490. 38608952 10.3168/jds.2023-24490

[186] Grupp SA, Corbacioglu S, Kang HJ, Teshima T, Khaw SL, Locatelli F, et al. Defibrotide plus best standard of care compared with best standard of care alone for the prevention of sinusoidal obstruction syndrome (HARMONY): a randomised, multicentre, phase 3 trial. Lancet Haematol. 2023;10(5):e333–45. 10.1016/S2352-3026(23)00011-X. 37001534 10.1016/S2352-3026(23)00011-X

[187] DeLeve LD, Wang X, Kanel GC, Ito Y, Bethea NW, McCuskey MK, et al. Decreased hepatic nitric oxide production contributes to the development of rat sinusoidal obstruction syndrome. Hepatology. 2003;38(4):900–8. 10.1053/jhep.2003.50383. 14512877 10.1053/jhep.2003.50383

[188] Kanou S, Miyashita T, Yamamoto Y, Takada S, Nakura M, Okazaki M, et al. Prophylactic effect of recombinant human soluble thrombomodulin for hepatic sinusoidal obstruction syndrome model mice. In Vivo. 2020;34(3):1037–45. 10.21873/invivo.11873. 32354890 10.21873/invivo.11873 PMC7279798

[189] Otaka F, Ito Y, Inoue T, Ohkubo H, Nishizawa N, Kojo K, et al. Thromboxane A(2) receptor signaling in endothelial cells attenuates monocrotaline-induced liver injury. Toxicol Appl Pharmacol. 2019;381:114733. 10.1016/j.taap.2019.114733. 31470032 10.1016/j.taap.2019.114733

[190] Schefczyk S, Luo X, Liang Y, Trippler M, Lu M, Wedemeyer H, et al. Poly(I:C) induces distinct liver cell type-specific responses in hepatitis B virus-transgenic mice in vitro, but fails to induce these signals in vivo. Viruses. 2023;15(5):1203. 10.3390/v15051203. 37243287 10.3390/v15051203 PMC10222874

[191] Zhao HJ, Han QJ, Wang G, Lin A, Xu DQ, Wang YQ, et al. Poly(I:C)-based rHBVvac therapeutic vaccine eliminates HBV via generation of HBV-specific CD8(+) effector memory T cells. Gut. 2019;68(11):2032–43. 10.1136/gutjnl-2017-315588. 30635406 10.1136/gutjnl-2017-315588

[192] Lin Y, Huang X, Wu J, Liu J, Chen M, Ma Z, et al. Pre-activation of Toll-like receptor 2 enhances CD8(+) T-cell responses and accelerates hepatitis B virus clearance in the mouse models. Front Immunol. 2018;9:1495. 10.3389/fimmu.2018.01495. 30008718 10.3389/fimmu.2018.01495 PMC6033958

[193] Gil M, Khouri L, Raurell I, Rafael D, Andrade F, Abasolo I, et al. Optimization of statin-loaded delivery nanoparticles for treating chronic liver diseases by targeting liver sinusoidal endothelial cells. Pharmaceutics. 2023;15(10):2463. 10.3390/pharmaceutics15102463. 37896223 10.3390/pharmaceutics15102463 PMC11340786

[194] Welz M, Eickhoff S, Abdullah Z, Trebicka J, Gartlan KH, Spicer JA, et al. Perforin inhibition protects from lethal endothelial damage during fulminant viral hepatitis. Nat Commun. 2018;9(1):4805. 10.1038/s41467-018-07213-x. 30442932 10.1038/s41467-018-07213-x PMC6237769

[195] Baiges A, Hernandez-Gea V, Bosch J. Pharmacologic prevention of variceal bleeding and rebleeding. Hepatol Int. 2018;12(Suppl 1):68–80. 10.1007/s12072-017-9833-y. 29210030 10.1007/s12072-017-9833-y

[196] Marrone G, Maeso-Diaz R, Garcia-Cardena G, Abraldes JG, Garcia-Pagan JC, Bosch J, et al. KLF2 exerts antifibrotic and vasoprotective effects in cirrhotic rat livers: behind the molecular mechanisms of statins. Gut. 2015;64(9):1434–43. 10.1136/gutjnl-2014-308338. 25500203 10.1136/gutjnl-2014-308338

[197] Abraldes JG, Albillos A, Banares R, Turnes J, Gonzalez R, Garcia-Pagan JC, et al. Simvastatin lowers portal pressure in patients with cirrhosis and portal hypertension: a randomized controlled trial. Gastroenterology. 2009;136(5):1651–8. 10.1053/j.gastro.2009.01.043. 19208350 10.1053/j.gastro.2009.01.043

[198] Parsa S, Dousti M, Mohammadi N, Abedanzadeh M, Dehdari Ebrahimi N, Dara M, et al. The effects of simvastatin-loaded nanoliposomes on human multilineage liver fibrosis microtissue. J Cell Mol Med. 2024;28(13):e18529. 10.1111/jcmm.18529. 38984945 10.1111/jcmm.18529 PMC11234647

[199] Chang CC, Lee WS, Hsieh HG, Chuang CL, Huang HC, Lee FY, et al. Selective cyclooxygenase inhibition by SC-560 improves hepatopulmonary syndrome in cirrhotic rats. PLoS ONE. 2017;12(6):e0179809. 10.1371/journal.pone.0179809. 28632747 10.1371/journal.pone.0179809 PMC5478154

[200] Matei V, Rodriguez-Vilarrupla A, Deulofeu R, Garcia-Caldero H, Fernandez M, Bosch J, et al. Three-day tetrahydrobiopterin therapy increases in vivo hepatic NOS activity and reduces portal pressure in CCl4 cirrhotic rats. J Hepatol. 2008;49(2):192–7. 10.1016/j.jhep.2008.04.014. 18534709 10.1016/j.jhep.2008.04.014

[201] Matei V, Rodriguez-Vilarrupla A, Deulofeu R, Colomer D, Fernandez M, Bosch J, et al. The eNOS cofactor tetrahydrobiopterin improves endothelial dysfunction in livers of rats with CCl4 cirrhosis. Hepatology. 2006;44(1):44–52. 10.1002/hep.21228. 16799985 10.1002/hep.21228

[202] Xavier FE, Blanco-Rivero J, Sastre E, Badimon L, Balfagon G. Simultaneous inhibition of TXA(2) and PGI(2) synthesis increases NO release in mesenteric resistance arteries from cirrhotic rats. Clin Sci (Lond). 2010;119(7):283–92. 10.1042/CS20090536. 20459396 10.1042/CS20090536

[203] Luna M, Karavitakis M. Treating portal hypertension in cirrhotic rats with AVE 9488. Liver Int. 2009;29(9):1448. 10.1111/j.1478-3231.2009.02093.x. 19659506 10.1111/j.1478-3231.2009.02093.x

[204] Biecker E, Trebicka J, Kang A, Hennenberg M, Sauerbruch T, Heller J. Treatment of bile duct-ligated rats with the nitric oxide synthase transcription enhancer AVE 9488 ameliorates portal hypertension. Liver Int. 2008;28(3):331–8. 10.1111/j.1478-3231.2008.01664.x. 18290775 10.1111/j.1478-3231.2008.01664.x

[205] De Gottardi A, Berzigotti A, Seijo S, D’Amico M, Thormann W, Abraldes JG, et al. Postprandial effects of dark chocolate on portal hypertension in patients with cirrhosis: results of a phase 2, double-blind, randomized controlled trial. Am J Clin Nutr. 2012;96(3):584–90. 10.3945/ajcn.112.040469. 22811444 10.3945/ajcn.112.040469

[206] Rodriguez-Vilarrupla A, Lavina B, Garcia-Caldero H, Russo L, Rosado E, Roglans N, et al. PPARalpha activation improves endothelial dysfunction and reduces fibrosis and portal pressure in cirrhotic rats. J Hepatol. 2012;56(5):1033–9. 10.1016/j.jhep.2011.12.008. 22245887 10.1016/j.jhep.2011.12.008

[207] Yang YY, Lee KC, Huang YT, Wang YW, Hou MC, Lee FY, et al. Effects of N-acetylcysteine administration in hepatic microcirculation of rats with biliary cirrhosis. J Hepatol. 2008;49(1):25–33. 10.1016/j.jhep.2008.02.012. 18490076 10.1016/j.jhep.2008.02.012

[208] Olaso E, Benedicto A, Lopategi A, Cossio FP, Arteta B. A synthetic analog of resveratrol inhibits the proangiogenic response of liver sinusoidal cells during hepatic metastasis. Biomol Ther (Seoul). 2022;30(2):162–9. 10.4062/biomolther.2021.062. 34873071 10.4062/biomolther.2021.062 PMC8902452

[209] Verbeke L, Mannaerts I, Schierwagen R, Govaere O, Klein S, Vander Elst I, et al. FXR agonist obeticholic acid reduces hepatic inflammation and fibrosis in a rat model of toxic cirrhosis. Sci Rep. 2016;6:33453. 10.1038/srep33453. 27634375 10.1038/srep33453 PMC5025787

[210] Rodriguez S, Raurell I, Torres-Arauz M, Garcia-Lezana T, Genesca J, Martell M. A nitric oxide-donating statin decreases portal pressure with a better toxicity profile than conventional statins in cirrhotic rats. Sci Rep. 2017;7:40461. 10.1038/srep40461. 28084470 10.1038/srep40461 PMC5233977

[211] Lin L, Cai M, Deng S, Huang W, Huang J, Huang X, et al. Amelioration of cirrhotic portal hypertension by targeted cyclooxygenase-1 siRNA delivery to liver sinusoidal endothelium with polyethylenimine grafted hyaluronic acid. Nanomedicine. 2017;13(7):2329–39. 10.1016/j.nano.2017.06.019. 28712920 10.1016/j.nano.2017.06.019

[212] Schwabl P, Hambruch E, Seeland BA, Hayden H, Wagner M, Garnys L, et al. The FXR agonist PX20606 ameliorates portal hypertension by targeting vascular remodelling and sinusoidal dysfunction. J Hepatol. 2017;66(4):724–33. 10.1016/j.jhep.2016.12.005. 27993716 10.1016/j.jhep.2016.12.005

[213] Pietrosi G, Fernandez-Iglesias A, Pampalone M, Ortega-Ribera M, Lozano JJ, Garcia-Caldero H, et al. Human amniotic stem cells improve hepatic microvascular dysfunction and portal hypertension in cirrhotic rats. Liver Int. 2020;40(10):2500–14. 10.1111/liv.14610. 32996708 10.1111/liv.14610

[214] Tsai HC, Li TH, Huang CC, Huang SF, Liu RS, Yang YY, et al. Beneficial effects of the peroxisome proliferator-activated receptor alpha/gamma agonist aleglitazar on progressive hepatic and splanchnic abnormalities in cirrhotic rats with portal hypertension. Am J Pathol. 2018;188(7):1608–24. 10.1016/j.ajpath.2018.03.018. 29929914 10.1016/j.ajpath.2018.03.018

[215] Boyer-Diaz Z, Aristu-Zabalza P, Andres-Rozas M, Robert C, Ortega-Ribera M, Fernandez-Iglesias A, et al. Pan-PPAR agonist lanifibranor improves portal hypertension and hepatic fibrosis in experimental advanced chronic liver disease. J Hepatol. 2021;74(5):1188–99. 10.1016/j.jhep.2020.11.045. 33278455 10.1016/j.jhep.2020.11.045

[216] Hu L, Su L, Dong Z, Wu Y, Lv Y, George J, et al. AMPK agonist AICAR ameliorates portal hypertension and liver cirrhosis via NO pathway in the BDL rat model. J Mol Med (Berl). 2019;97(3):423–34. 10.1007/s00109-019-01746-4. 30721324 10.1007/s00109-019-01746-4 PMC6394556

[217] Gracia-Sancho J, Manicardi N, Ortega-Ribera M, Maeso-Diaz R, Guixe-Muntet S, Fernandez-Iglesias A, et al. Emricasan ameliorates portal hypertension and liver fibrosis in cirrhotic rats through a hepatocyte-mediated paracrine mechanism. Hepatol Commun. 2019;3(7):987–1000. 10.1002/hep4.1360. 31304452 10.1002/hep4.1360 PMC6601324

[218] Zhang R, Chen J, Liu D, Wang Y. Urotensin II receptor antagonist reduces hepatic resistance and portal pressure through enhanced eNOS-dependent HSC vasodilatation in CCl(4)-induced cirrhotic rats. Front Med. 2019;13(3):398–408. 10.1007/s11684-019-0689-5. 30820806 10.1007/s11684-019-0689-5

[219] Tripathi DM, Vilaseca M, Lafoz E, Garcia-Caldero H, Viegas Haute G, Fernandez-Iglesias A, et al. Simvastatin prevents progression of acute on chronic liver failure in rats with cirrhosis and portal hypertension. Gastroenterology. 2018;155(5):1564–77. 10.1053/j.gastro.2018.07.022. 30055171 10.1053/j.gastro.2018.07.022

[220] Pose E, Sola E, Lozano JJ, Juanola A, Sidorova J, Zaccherini G, et al. Treatment with simvastatin and rifaximin restores the plasma metabolomic profile in patients with decompensated cirrhosis. Hepatol Commun. 2022;6(5):1100–12. 10.1002/hep4.1881. 34964311 10.1002/hep4.1881 PMC9035579

[221] Yu Z, Guo J, Liu Y, Wang M, Liu Z, Gao Y, et al. Nano delivery of simvastatin targets liver sinusoidal endothelial cells to remodel tumor microenvironment for hepatocellular carcinoma. J Nanobiotechnology. 2022;20(1):9. 10.1186/s12951-021-01205-8. 34983554 10.1186/s12951-021-01205-8 PMC8725360

[222] Tanaka T, Koga H, Suzuki H, Iwamoto H, Sakaue T, Masuda A, et al. Anti-PD-L1 antibodies promote cellular proliferation by activating the PD-L1-AXL signal relay in liver cancer cells. Hepatol Int. 2024;18(3):984–97. 10.1007/s12072-023-10572-3. 37553470 10.1007/s12072-023-10572-3

[223] Duffy AG, Ulahannan SV, Makorova-Rusher O, Rahma O, Wedemeyer H, Pratt D, et al. Tremelimumab in combination with ablation in patients with advanced hepatocellular carcinoma. J Hepatol. 2017;66(3):545–51. 10.1016/j.jhep.2016.10.029. 27816492 10.1016/j.jhep.2016.10.029 PMC5316490

[224] Bruix J, Qin S, Merle P, Granito A, Huang YH, Bodoky G, et al. Regorafenib for patients with hepatocellular carcinoma who progressed on sorafenib treatment (RESORCE): a randomised, double-blind, placebo-controlled, phase 3 trial. Lancet. 2017;389(10064):56–66. 10.1016/S0140-6736(16)32453-9. 27932229 10.1016/S0140-6736(16)32453-9

[225] Ho WJ, Zhu Q, Durham J, Popovic A, Xavier S, Leatherman J, et al. Neoadjuvant cabozantinib and nivolumab converts locally advanced HCC into resectable disease with enhanced antitumor immunity. Nat Cancer. 2021;2(9):891–903. 10.1038/s43018-021-00234-4. 34796337 10.1038/s43018-021-00234-4 PMC8594857

[226] Li W, Shen S, Wu S, Chen Z, Hu C, Yan R. Regulation of tumorigenesis and metastasis of hepatocellular carcinoma tumor endothelial cells by microRNA-3178 and underlying mechanism. Biochem Biophys Res Commun. 2015;464(3):881–7. 10.1016/j.bbrc.2015.07.057. 26182877 10.1016/j.bbrc.2015.07.057

[227] Jaeschke H, Ramachandran A. Acetaminophen hepatotoxicity: paradigm for understanding mechanisms of drug-induced liver injury. Annu Rev Pathol. 2024;19:453–78. 10.1146/annurev-pathmechdis-051122-094016. 38265880 10.1146/annurev-pathmechdis-051122-094016 PMC11131139

[228] Licata A, Minissale MG, Stankeviciute S, Sanabria-Cabrera J, Lucena MI, Andrade RJ, et al. N-Acetylcysteine for preventing acetaminophen-induced liver injury: a comprehensive review. Front Pharmacol. 2022;13:828565. 10.3389/fphar.2022.828565. 36034775 10.3389/fphar.2022.828565 PMC9399785

[229] Zhang Q, Jiang L, Yu J, Tian L, Guo T, Di B, et al. Palmitate up-regulates laminin expression via ROS/integrin alphavbeta3 pathway in hlsecs. Oncotarget. 2019;10(41):4083–90. 10.18632/oncotarget.26937. 31289608 10.18632/oncotarget.26937 PMC6609245

[230] Granados-Castro LF, Rodriguez-Rangel DS, Fernandez-Rojas B, Leon-Contreras JC, Hernandez-Pando R, Medina-Campos ON, et al. Curcumin prevents paracetamol-induced liver mitochondrial alterations. J Pharm Pharmacol. 2016;68(2):245–56. 10.1111/jphp.12501. 26773315 10.1111/jphp.12501

[231] Dogaru G, Bulboaca AE, Gheban D, Boarescu PM, Rus V, Festila D, et al. Effect of liposomal curcumin on acetaminophen hepatotoxicity by down-regulation of oxidative stress and matrix metalloproteinases. In Vivo. 2020;34(2):569–82. 10.21873/invivo.11809. 32111755 10.21873/invivo.11809 PMC7157894

[232] Ahmad MM, Rezk NA, Fawzy A, Sabry M. Protective effects of curcumin and silymarin against paracetamol induced hepatotoxicity in adult male albino rats. Gene. 2019;712:143966. 10.1016/j.gene.2019.143966. 31279711 10.1016/j.gene.2019.143966

[233] Abdulrazzaq AM, Badr M, Gammoh O, Abu Khalil AA, Ghanim BY, Alhussainy TM, et al. Hepatoprotective actions of ascorbic acid, alpha lipoic acid and silymarin or their combination against acetaminophen-induced hepatotoxicity in rats. Medicina (Kaunas). 2019;55(5):181. 10.3390/medicina55050181. 31117289 10.3390/medicina55050181 PMC6571961

[234] Noh JR, Kim YH, Hwang JH, Choi DH, Kim KS, Oh WK, et al. Sulforaphane protects against acetaminophen-induced hepatotoxicity. Food Chem Toxicol. 2015;80:193–200. 10.1016/j.fct.2015.03.020. 25818464 10.1016/j.fct.2015.03.020

[235] Hamid A, Lee LS, Karim SR, Jufri NF. Hepatoprotective effects of zerumbone against paracetamol-induced acute hepatotoxicity in rats. Malays J Med Sci. 2018;25(2):64–71. 10.21315/mjms2018.25.2.7. 30918456 10.21315/mjms2018.25.2.7 PMC6422587

[236] Ali BH, Mousa HM, El-Mougy S. The effect of a water extract and anthocyanins of *Hibiscus sabdariffa* L. on paracetamol-induced hepatoxicity in rats. Phytother Res. 2003;17(1):56–9. 10.1002/ptr.1084. 12557248 10.1002/ptr.1084

[237] Abd El-Ghffar EA, El-Nashar HA, Eldahshan OA, Singab AN. GC-MS analysis and hepatoprotective activity of the n-hexane extract of Acrocarpus fraxinifolius leaves against paracetamol-induced hepatotoxicity in male albino rats. Pharm Biol. 2017;55(1):441–9. 10.1080/13880209.2016.1246575. 27937037 10.1080/13880209.2016.1246575 PMC6130506

[238] Chandrasekaran VR, Chien SP, Hsu DZ, Chang YC, Liu MY. Effects of sesame oil against after the onset of acetaminophen-induced acute hepatic injury in rats. JPEN J Parenter Enteral Nutr. 2010;34(5):567–73. 10.1177/0148607110362584. 20852187 10.1177/0148607110362584

[239] El Morsy EM, Kamel R. Protective effect of artichoke leaf extract against paracetamol-induced hepatotoxicity in rats. Pharm Biol. 2015;53(2):167–73. 10.3109/13880209.2014.913066. 25243875 10.3109/13880209.2014.913066

[240] Liao J, Lu Q, Li Z, Li J, Zhao Q, Li J. Acetaminophen-induced liver injury: molecular mechanism and treatments from natural products. Front Pharmacol. 2023;14:1122632. 10.3389/fphar.2023.1122632. 37050900 10.3389/fphar.2023.1122632 PMC10083499

[241] Li X, Lao R, Lei J, Chen Y, Zhou Q, Wang T, et al. Natural products for acetaminophen-induced acute liver injury: a review. Molecules. 2023;28(23):7901. 10.3390/molecules28237901. 38067630 10.3390/molecules28237901 PMC10708418

[242] Mavrikou I, Chatzidimitriou D, Skoura L, Nikolousis E, Sakellari I, Gavriilaki E. Molecular advances in sinusoidal obstruction Syndrome/Veno-Occlusive disease. Int J Mol Sci. 2023;24(6):5620. 10.3390/ijms24065620. 36982695 10.3390/ijms24065620 PMC10051970

[243] Bonifazi F, Barbato F, Ravaioli F, Sessa M, Defrancesco I, Arpinati M, et al. Diagnosis and treatment of VOD/SOS after allogeneic hematopoietic stem cell transplantation. Front Immunol. 2020;11:489. 10.3389/fimmu.2020.00489. 32318059 10.3389/fimmu.2020.00489 PMC7147118

[244] Oda S, Takeuchi M, Akai S, Shirai Y, Tsuneyama K, Yokoi T. miRNA in rat liver sinusoidal endothelial cells and hepatocytes and application to circulating biomarkers that discern pathogenesis of liver injuries. Am J Pathol. 2018;188(4):916–28. 10.1016/j.ajpath.2017.12.007. 29353062 10.1016/j.ajpath.2017.12.007

[245] Yakushijin K, Ikezoe T, Ohwada C, Kudo K, Okamura H, Goto H, et al. Clinical effects of recombinant thrombomodulin and defibrotide on sinusoidal obstruction syndrome after allogeneic hematopoietic stem cell transplantation. Bone Marrow Transplant. 2019;54(5):674–80. 10.1038/s41409-018-0304-4. 30127466 10.1038/s41409-018-0304-4

[246] Navarro VJ, Khan I, Bjornsson E, Seeff LB, Serrano J, Hoofnagle JH. Liver injury from herbal and dietary supplements. Hepatology. 2017;65(1):363–73. 10.1002/hep.28813. 27677775 10.1002/hep.28813 PMC5502701

[247] Zhu J, Seo JE, Wang S, Ashby K, Ballard R, Yu D, et al. The development of a database for herbal and dietary supplement induced liver toxicity. Int J Mol Sci. 2018;19(10):2955. 10.3390/ijms19102955. 30274144 10.3390/ijms19102955 PMC6213387

[248] Halegoua-DeMarzio D, Navarro V. Challenges in herbal-induced liver injury identification and prevention. Liver Int. 2025;45(3):e16071. 10.1111/liv.16071. 39136211 10.1111/liv.16071 PMC11815602

[249] Limmer A, Ohl J, Kurts C, Ljunggren HG, Reiss Y, Groettrup M, et al. Efficient presentation of exogenous antigen by liver endothelial cells to CD8+ T cells results in antigen-specific T-cell tolerance. Nat Med. 2000;6(12):1348–54. 10.1038/82161. 11100119 10.1038/82161

[250] Huang S, Wu J, Gao X, Zou S, Chen L, Yang X, et al. LSECs express functional NOD1 receptors: a role for NOD1 in LSEC maturation-induced T cell immunity in vitro. Mol Immunol. 2018;101:167–75. 10.1016/j.molimm.2018.06.002. 29944986 10.1016/j.molimm.2018.06.002

[251] Tuncer C, Oo YH, Murphy N, Adams DH, Lalor PF. The regulation of T-cell recruitment to the human liver during acute liver failure. Liver Int. 2013;33(6):852–63. 10.1111/liv.12182. 23617240 10.1111/liv.12182

[252] Falero-Perez J, Song YS, Zhao Y, Teixeira L, Sorenson CM, Sheibani N. Cyp1b1 expression impacts the angiogenic and inflammatory properties of liver sinusoidal endothelial cells. PLoS ONE. 2018;13(10):e0206756. 10.1371/journal.pone.0206756. 30372497 10.1371/journal.pone.0206756 PMC6205649

[253] Martin-Armas M, Simon-Santamaria J, Pettersen I, Moens U, Smedsrod B, Sveinbjornsson B. Toll-like receptor 9 (TLR9) is present in murine liver sinusoidal endothelial cells (LSECs) and mediates the effect of CpG-oligonucleotides. J Hepatol. 2006;44(5):939–46. 10.1016/j.jhep.2005.09.020. 16458386 10.1016/j.jhep.2005.09.020

[254] Cheluvappa R, Denning GM, Lau GW, Grimm MC, Hilmer SN, Le Couteur DG. Pseudomonas aeruginosa and the hyperlipidaemia of sepsis. Pathology. 2009;41(7):615–21. 10.3109/00313020903257764. 20001339 10.3109/00313020903257764

[255] Cheluvappa R, Denning GM, Lau GW, Grimm MC, Hilmer SN, Le Couteur DG. Pathogenesis of the hyperlipidemia of gram-negative bacterial sepsis may involve pathomorphological changes in liver sinusoidal endothelial cells. Int J Infect Dis. 2010;14(10):e857–67. 10.1016/j.ijid.2010.02.2263. 20609608 10.1016/j.ijid.2010.02.2263

[256] Heesch K, Raczkowski F, Schumacher V, Hunemorder S, Panzer U, Mittrucker HW. The function of the chemokine receptor CXCR6 in the T cell response of mice against Listeria monocytogenes. PLoS ONE. 2014;9(5):e97701. 10.1371/journal.pone.0097701. 24832098 10.1371/journal.pone.0097701 PMC4022635

[257] Liu J, Jiang M, Ma Z, Dietze KK, Zelinskyy G, Yang D, et al. TLR1/2 ligand-stimulated mouse liver endothelial cells secrete IL-12 and trigger CD8+ T cell immunity in vitro. J Immunol. 2013;191(12):6178–90. 10.4049/jimmunol.1301262. 24227786 10.4049/jimmunol.1301262

[258] Wu J, Huang S, Zhao X, Chen M, Lin Y, Xia Y, et al. Poly(I:C) treatment leads to interferon-dependent clearance of hepatitis B virus in a hydrodynamic injection mouse model. J Virol. 2014;88(18):10421–31. 10.1128/JVI.00996-14. 24920792 10.1128/JVI.00996-14 PMC4178898

[259] Lozach PY, Amara A, Bartosch B, Virelizier JL, Arenzana-Seisdedos F, Cosset FL, et al. C-type lectins L-SIGN and DC-SIGN capture and transmit infectious hepatitis C virus pseudotype particles. J Biol Chem. 2004;279(31):32035–45. 10.1074/jbc.M402296200. 15166245 10.1074/jbc.M402296200

[260] Sun LJ, Yu JW, Shi YG, Zhang XY, Shu MN, Chen MY. Hepatitis C virus core protein induces dysfunction of liver sinusoidal endothelial cell by down-regulation of silent information regulator 1. J Med Virol. 2018;90(5):926–35. 10.1002/jmv.25034. 29350417 10.1002/jmv.25034

[261] Seckert CK, Renzaho A, Tervo HM, Krause C, Deegen P, Kuhnapfel B, et al. Liver sinusoidal endothelial cells are a site of murine cytomegalovirus latency and reactivation. J Virol. 2009;83(17):8869–84. 10.1128/JVI.00870-09. 19535440 10.1128/JVI.00870-09 PMC2738169

[262] Coppi A, Tewari R, Bishop JR, Bennett BL, Lawrence R, Esko JD, et al. Heparan sulfate proteoglycans provide a signal to Plasmodium sporozoites to stop migrating and productively invade host cells. Cell Host Microbe. 2007;2(5):316–27. 10.1016/j.chom.2007.10.002. 18005753 10.1016/j.chom.2007.10.002 PMC2117360

[263] Bosch J, Gracia-Sancho J, Abraldes JG. Cirrhosis as new indication for statins. Gut. 2020;69(5):953–62. 10.1136/gutjnl-2019-318237. 32139553 10.1136/gutjnl-2019-318237

[264] Niaz Q, Tavangar SM, Mehreen S, Ghazi-Khansari M, Jazaeri F. Evaluation of statins as a new therapy to alleviate chronotropic dysfunction in cirrhotic rats. Life Sci. 2022;308:120966. 10.1016/j.lfs.2022.120966. 36150464 10.1016/j.lfs.2022.120966

[265] Maeso-Diaz R, Ortega-Ribera M, Lafoz E, Lozano JJ, Baiges A, Frances R, et al. Aging influences hepatic microvascular biology and liver fibrosis in advanced chronic liver disease. Aging Dis. 2019;10(4):684–98. 10.14336/AD.2019.0127. 31440376 10.14336/AD.2019.0127 PMC6675529

[266] Hide D, Gil M, Andrade F, Rafael D, Raurell I, Bravo M, et al. Simvastatin-loaded polymeric micelles are more effective and less toxic than conventional statins in a pre-clinical model of advanced chronic liver disease. Nanomedicine. 2020;29:102267. 10.1016/j.nano.2020.102267. 32681987 10.1016/j.nano.2020.102267

[267] Zafra C, Abraldes JG, Turnes J, Berzigotti A, Fernandez M, Garcia-Pagan JC, et al. Simvastatin enhances hepatic nitric oxide production and decreases the hepatic vascular tone in patients with cirrhosis. Gastroenterology. 2004;126(3):749–55. 10.1053/j.gastro.2003.12.007. 14988829 10.1053/j.gastro.2003.12.007

[268] Chapin S, Kaplan DE, Taddei T, Mahmud N. Association between statin exposure and short-term mortality in patients with high-grade acute-on-chronic liver failure. JHEP Rep. 2023;5(6):100740. 10.1016/j.jhepr.2023.100740. 37215188 10.1016/j.jhepr.2023.100740 PMC10193237

[269] Gracia-Sancho J, Maeso-Diaz R, Fernandez-Iglesias A, Navarro-Zornoza M, Bosch J. New cellular and molecular targets for the treatment of portal hypertension. Hepatol Int. 2015;9(2):183–91. 10.1007/s12072-015-9613-5. 25788198 10.1007/s12072-015-9613-5

[270] Manavski Y, Abel T, Hu J, Kleinlutzum D, Buchholz CJ, Belz C, et al. Endothelial transcription factor KLF2 negatively regulates liver regeneration via induction of activin a. Proc Natl Acad Sci U S A. 2017;114(15):3993–8. 10.1073/pnas.1613392114. 28348240 10.1073/pnas.1613392114 PMC5393189

[271] Verbeke L, Farre R, Trebicka J, Komuta M, Roskams T, Klein S, et al. Obeticholic acid, a farnesoid x receptor agonist, improves portal hypertension by two distinct pathways in cirrhotic rats. Hepatology. 2014;59(6):2286–98. 10.1002/hep.26939. 24259407 10.1002/hep.26939

[272] de Beijer MTA, Bezstarosti K, Luijten R, Doff WAS, Boor PPC, Pieterman RFA, et al. Immunopeptidome of hepatocytes isolated from patients with HBV infection and hepatocellular carcinoma. JHEP Rep. 2022;4(11):100576. 10.1016/j.jhepr.2022.100576. 36185575 10.1016/j.jhepr.2022.100576 PMC9523389

[273] Jin H, Qin S, He J, Xiao J, Li Q, Mao Y, et al. New insights into checkpoint inhibitor immunotherapy and its combined therapies in hepatocellular carcinoma: from mechanisms to clinical trials. Int J Biol Sci. 2022;18(7):2775–94. 10.7150/ijbs.70691. 35541908 10.7150/ijbs.70691 PMC9066095

[274] Qin S, Kudo M, Meyer T, Bai Y, Guo Y, Meng Z, et al. Tislelizumab vs sorafenib as first-line treatment for unresectable hepatocellular carcinoma: a phase 3 randomized clinical trial. JAMA Oncol. 2023;9(12):1651–9. 10.1001/jamaoncol.2023.4003. 37796513 10.1001/jamaoncol.2023.4003 PMC10557031

[275] Finn RS, Kudo M, Barnes G, Meyer T, Boisserie F, Abdrashitov R, et al. Tislelizumab versus sorafenib in first-line treatment of unresectable hepatocellular carcinoma: impact on health-related quality of life in RATIONALE-301 study. Liver Cancer. 2024;13(5):548–60. 10.1159/000537966. 39435268 10.1159/000537966 PMC11493386

[276] Qin S, Chan SL, Gu S, Bai Y, Ren Z, Lin X, et al. Camrelizumab plus rivoceranib versus sorafenib as first-line therapy for unresectable hepatocellular carcinoma (CARES-310): a randomised, open-label, international phase 3 study. Lancet. 2023;402(10408):1133–46. 10.1016/S0140-6736(23)00961-3. 37499670 10.1016/S0140-6736(23)00961-3

[277] Andrews TS, Atif J, Liu JC, Perciani CT, Ma XZ, Thoeni C, et al. Single-cell, single-nucleus, and spatial RNA sequencing of the human liver identifies cholangiocyte and mesenchymal heterogeneity. Hepatol Commun. 2022;6(4):821–40. 10.1002/hep4.1854. 34792289 10.1002/hep4.1854 PMC8948611

[278] Brazovskaja A, Gomes T, Holtackers R, Wahle P, Körner C, He Z, et al. Cell atlas of the regenerating human liver after portal vein embolization. Nat Commun. 2024;15(1):5827. 10.1038/s41467-024-49236-7. 38992008 10.1038/s41467-024-49236-7 PMC11239663

[279] Restagno D, Saraswat M, Aziz PV, Smith K, Roberts AJ, Hintze J, et al. Mrc1 (MMR, CD206) controls the blood proteome in reducing inflammation, age-associated organ dysfunction and mortality in sepsis. Nat Commun. 2025;16:6267. 10.1038/s41467-025-61346-4. 40624023 10.1038/s41467-025-61346-4 PMC12234678

[280] Al-Mughalles AS, Ma J, Ma H, Zhang C, Xiao H, Ma X, et al. Liver sinusoidal endothelial cell fenestrations in metabolic liver disease: from molecular mechanisms to therapeutic perspectives. Cell Commun Signal. 2026;24:351. 10.1186/s12964-026-02997-8. 42286714 10.1186/s12964-026-02997-8 PMC13262446

[281] DeLeve LD, Wang X, Hu L, McCuskey MK, McCuskey RS. Rat liver sinusoidal endothelial cell phenotype is maintained by paracrine and autocrine regulation. Am J Physiol Gastrointest Liver Physiol. 2004;287(4):G757–63. 10.1152/ajpgi.00017.2004. 15191879 10.1152/ajpgi.00017.2004

[282] Zhang LF, Ru JW, Liang SQ, Zhang MM, Zhang CY, Liu JQ, et al. Nanotherapy targeting liver sinusoidal endothelial cells in liver fibrosis. J Control Release. 2026;394:114864. 10.1016/j.jconrel.2026.114864. 41933801 10.1016/j.jconrel.2026.114864

